# Evaluation of the tuberculosis notification impact from an intermediary-facilitated public–private mix intervention in Viet Nam: A population-level quasi-experimental study

**DOI:** 10.1371/journal.pmed.1005144

**Published:** 2026-09-23

**Authors:** Luan Nguyen Quang Vo, Thuy Thi Thu Dong, Huy Ba Huynh, Binh Anh Luong, Huong Thi Lan Mo, Andrew James Codlin, Rachel Forse, Thuy Doan To Mai, Lan Phuong Nguyen, Jacob Creswell, Luong Van Dinh, Hoa Binh Nguyen, Phu Xuan Vu, Tuan Dinh Nguyen, Lan Huu Nguyen, Thi Minh Ha Dang, Son Van Luu, Nam Hoang Do, Huyen Thanh Truong, Phan Do Nguyen, Tushar Garg, Jad Shedrawy, Kristi Sidney Annerstedt, Minh Huy Pham, Jamie Tonsing, Tom Wingfield, Mohammed Ahmed Yassin, Nhung Viet Nguyen, Knut Lönnroth

**Affiliations:** 1 Friends for International TB Relief, Ha Noi, Viet Nam; 2 Department of Global Public Health, Karolinska Institutet, Stockholm, Sweden; 3 University of Sydney Vietnam Institute, Ho Chi Minh City, Viet Nam; 4 Stop TB Partnership, Geneva, Switzerland; 5 National Lung Hospital, Ha Noi, Viet Nam; 6 Pham Ngoc Thach Hospital, Ho Chi Minh City, Viet Nam; 7 The Swedish Institute for Health Economics, Stockholm, Sweden; 8 United States Agency for International Development, Ha Noi, Viet Nam; 9 Global Fund to Fight AIDS, Tuberculosis and Malaria, Geneva, Switzerland; 10 Centre for Tuberculosis Research, Department of Clinical Sciences, Liverpool School of Tropical Medicine, Liverpool, United Kingdom; 11 Tropical and Infectious Diseases Unit, Liverpool University Hospital NHS Foundation Trust, Liverpool, United Kingdom; 12 VNU University of Medicine and Pharmacy, Ha Noi, Viet Nam; Duke University, UNITED STATES OF AMERICA

## Abstract

**Background:**

Treatment coverage gaps remain a major barrier to ending tuberculosis (TB). Public–private mix (PPM) entails the engagement of healthcare providers outside of national TB programs (NTP). PPM strategies are promoted to improve TB notification, particularly in high-burden settings with fragmented care systems, and intermediary agencies are recognized as effective mechanisms to operationalize PPM by bridging NTPs and out-of-network healthcare providers. However, population-level evidence is rare, as most evaluations have been limited to pilot studies, small geographic areas, or uncontrolled designs. This study documents the population-level association of a PPM intervention with TB notifications through the use of unbiased analytical methods to account for heterogeneity from the intervention’s staggered deployment and dynamic intensity.

**Methods and findings:**

We present a non-randomized, quasi-experimental evaluation of a large-scale intermediary-facilitated PPM intervention conducted between 2020 and 2023 in 15 provinces of Viet Nam, covering 40.6 million people equivalent to 40% of the population. The intervention engaged 3,154 non-NTP providers in TB care, linking them to the NTP through systematic screening, referral, and reporting mechanisms. The model emphasized active case finding, provider incentives, patient support and integrated data systems to raise notifications as the primary outcome. We analyzed quarterly province-level TB notification data from 2016 to 2025 using a heterogeneity-robust extended two-way fixed-effects framework to estimate the average treatment effect on the treated. The analysis adjusted for province and calendar-quarter fixed effects, and for COVID-19 disruptions. Using Poisson pseudo-maximum-likelihood fixed-effects models, we estimated dose–response effects and post-intervention patterns and triangulated effects with an interrupted time-series analysis of national notifications and repeated all analyses using the intermediate outcome of PPM notifications. Lastly, we calculated marginal cost per additional notification from a funder perspective. The intervention was associated with 316.8 additional notifications per province-quarter (95% confidence interval [CI] [23.4, 610.2]; *p* = 0.034). Regarding dose–response effects, we observed a 1.4% increase in notifications per 100 verbal assessments per 100,000 population (incidence rate ratio [IRR]=1.00014; 95% CI [1.00002, 1.00025]; *p* = 0.018). National models suggested 3,206 (95% CI [1,658, 4,763]; *p* < 0.001) additional notifications annually with a 3.5% intensity-associated notification increase (IRR = 1.00035; 95% CI [1.00018, 1.00052]; *p* < 0.001). Using PPM notifications produced concordant results. The costs per additional notification were US$ 166-US$ 458. The main limitation was the non-randomized design, with high-burden provinces purposively selected as intervention areas. This was mitigated through conservative control selection and triangulation across methods.

**Conclusions:**

This study furnishes robust, policy-relevant evidence that intermediary-facilitated PPM implemented at scale in Viet Nam was associated with significant, dose–responsive increases in PPM and TB notifications at provincial and national levels. These findings promote intermediary agency models as an effective mechanism to operationalize PPM at scale and to generate public health benefits in closing TB treatment coverage gaps. In an era of constrained global health financing, leveraging existing provider networks through intermediary-facilitated PPM offers an affordable and scalable strategy to sustain momentum towards ending TB.

## Author summary

**Why was this study done?:**

- Millions of people who develop tuberculosis each year are never reported to national health programs, in part because they seek care from private practices, pharmacies, clinics and hospitals that operate outside the public system.
- Bringing these providers into the national response is widely recommended, but there has been little strong evidence on whether it actually works when applied at a population level.

**What did the researchers do and find?:**

- We studied an intervention in Viet Nam that used an intermediary agency to connect private and other non-program providers to Viet Nam’s National Tuberculosis Control Programme across 15 provinces over four years.
- Using national records and methods designed to separate the program’s effect from other changes over time, we found that it was associated with a clear and sustained increase in the number of people reported with tuberculosis, even during the COVID-19 pandemic, at a reasonable cost.

**What do these findings mean?:**

- Connecting private providers to national programs through an intermediary agency is an affordable, practical way to find more people with tuberculosis, especially as global health funding becomes more constrained.
- The main limitation was the non-randomized selection of the intervention provinces, which may have overstated the number of additional notifications from the intervention. This was mitigated through conservative selection of the control area and use of multiple methods and outcomes to triangulate the results.

## Abstract 3

Using a population-level quasi-experimental study design, Luan Nguyen Quang Vo and colleagues assess the effects of a public-private mix intervention implemented in Viet Nam and designed to increase tuberculosis notifications, with the aim that such approaches could ultimately increase treatment coverage.

## Background

Tuberculosis (TB) is among the top 10 causes of death worldwide. In 2024, an estimated 10.7 million people developed TB disease. A key challenge is the treatment coverage gap of 2.4 million people with TB (22%) unreached by national TB programs (NTP), which fuels TB mortality, while sustaining community transmission and incidence [1]. Unreached persons with TB can be bifurcated into undiagnosed and unreported groups, with the latter consisting of privately treated but not reported persons with TB [2], highlighting the critical role of unengaged healthcare providers [3,4]. Recognizing their importance in achieving universal quality-assured TB care, the World Health Organization (WHO) has issued guidance on engaging all healthcare providers [5]. This ignited public–private mix (PPM) initiatives in many high-burden settings with fragmented health systems [6].

A consistent enabler of successful PPM implementation has been the engagement of an intermediary to bridge trust divides, foster dialogue and catalyze partnerships [7]. Studies described the involvement of such an intermediary for brokering tenuous relationships between public and private sectors as “indispensable” [8], and found that intermediary-facilitated PPM outperformed NTP-managed models [9]. The intermediary model’s prominence reached critical mass with the Private Provider Interface Agencies (PPIA) in India [10]. These PPIAs were responsible for engaging the health system’s large diversity of private providers with minimal disruption of existing market dynamics to improve TB notifications, while outsourcing services and costs [11]. However, robust population-level impact evaluations of these intermediary models remain rare.

Viet Nam is among the 30 high TB burden countries with 184,000 people with new TB and 12,000 TB-related deaths yearly. With a treatment coverage of 58%, it places among the top 10 countries with the largest gap [1]. As 45%−75% of initial health-seeking occurs at non-NTP providers [12,13], half of the gap is estimated to result from underreporting of private treatment [14], where quality can deviate markedly from national and international standards [15,16]. The country passed legislation on involving non-NTP providers in TB control in 2013 (Circular 02). It encompassed four collaboration models: (1) referral; (2) diagnosis and referral; (3) treatment supervision; and (4) full-service TB unit. In 2014, the four traditional PPM models yielded 9% of national notifications, but had not risen beyond that [17].

To enhance the impact of PPM, a PPIA was launched in Viet Nam in 2017, named PPM Model 5 to distinguish it from the four established models. This model offered a package of technical, financial and administrative support to providers and their clients. In return, providers facilitated symptom and Chest X-ray (CXR) screening, and referred persons with radiographic abnormalities for molecular diagnosis and treatment, or reported persons privately treated to the NTP. After confirmatory pilots [18,19], the model was expanded from 3 to 15 provinces in 2020. Here, we report its outputs and outcomes, impact on TB notifications, and associated costs.

## Methods

### Study aims and design

We conducted a non-randomized, quasi-experimental evaluation to assess the effect on TB notifications of a population-level PPM intervention implemented through an intermediary agency in Viet Nam. The specific study objectives were to: (1) characterize provider engagement and contributions to TB care; (2) describe diagnostic and treatment yields; (3) estimate average treatment and dose–response effects on TB notifications at subnational and national levels; and (4) estimate marginal costs per additional TB notification.

**Fig 1:**
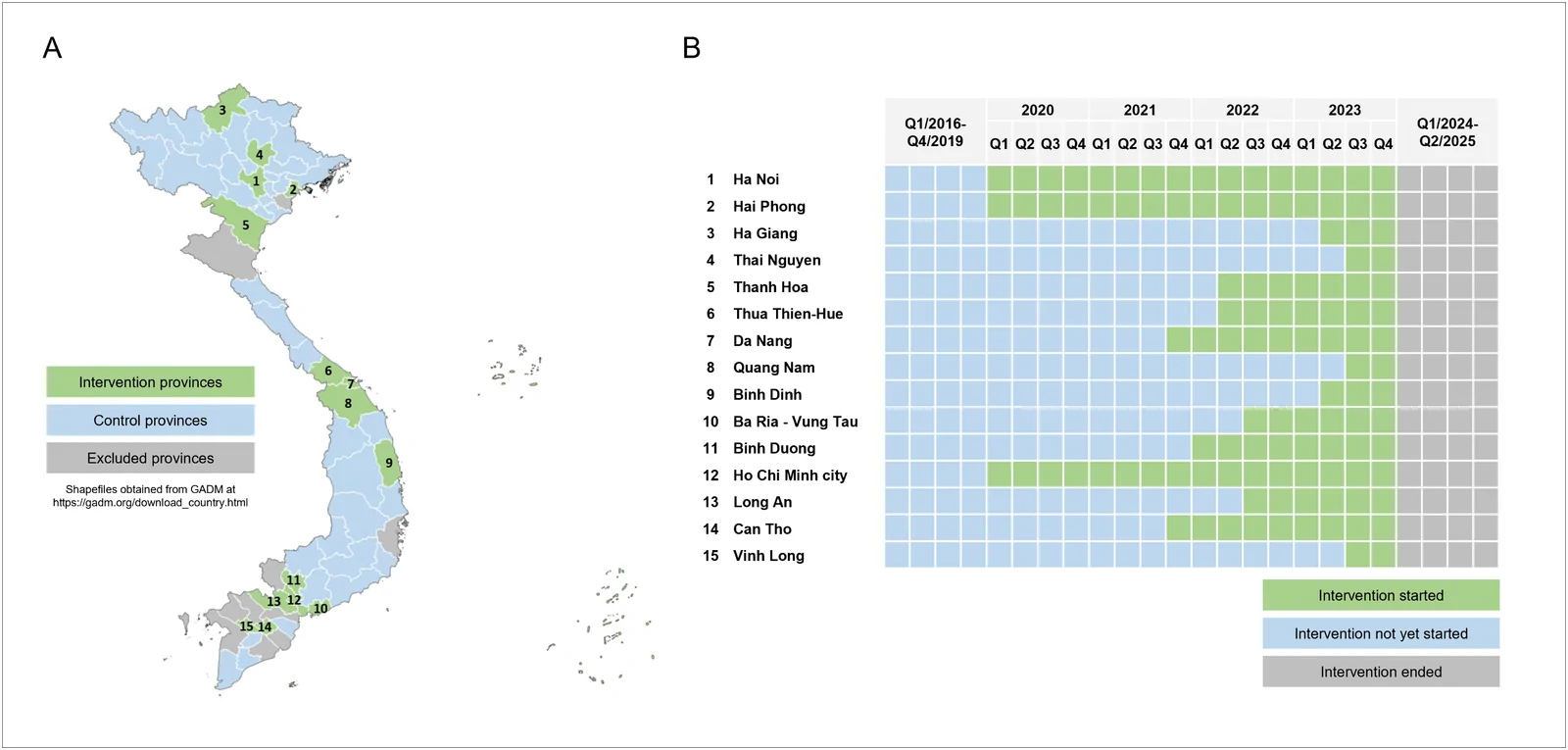
Study setting and staggered implementation of the intervention. **(A)** Map of Vietnam showing intervention provinces (green) and control provinces (blue). Ten provinces (gray) were excluded because of confounding active case finding and public–private mix activities, for which there was insufficient information to isolate the public–private mix impact. **(B)** Timeline of staggered deployment across intervention provinces, with quarters before intervention deployment shown in blue, quarters with intervention activities shown in green, and quarters after intervention cessation shown in gray. Of 240 possible province-quarters during the Q1/2020 to Q4/2023 period, 112 had non-zero intervention intensity, defined as at least one person screened; Q, Quarters. The map was created using QGIS software, with source shape files coming from GADM. The relevant link is here: https://gadm.org/download_country.html.

In 2025, Viet Nam had a population of 102.5 million in 63 provinces. Intervention provinces accounted for 40.6 million people (39.6% of the national population), while control provinces covered 45.1 million people (44.0%, Fig 1A). Ten provinces with 16.8 million people (16.4%) were excluded due to confounding interventions. Intervention provinces notified 44,096 (43.9%) persons with TB in 2019 with higher baseline TB notification rates (108.6 per 100,000) and estimated treatment coverage (60%) than control provinces (34,405 [34.3%]; 76.3 per 100,000; 46%), reflecting their selection as high-burden settings (Table 1). At baseline, PPM contributed 9.4% of 2019 notifications in the intervention provinces, 6.6% in the control provinces, and 22.2% in the excluded provinces. Disaggregated provincial characteristics are in Table F in S1 Appendix.

**Table 1:** Baseline characteristics of intervention, control, and excluded provinces, 2019.

	Intervention (*n* = 15)	Control (*n* = 38)	Excluded (*n* = 10)	Total (*n* = 63)
Total population, millions (2025)	40.6	45.1	16.8	102.5
TB prevalence rate, per 100,000 population^a^	327	302	348	319
Estimated TB incidence	73,034	74,604	32,065	179,703
TB notifications, baseline (2019)	44,096	34,405	21,855	100,356
Notification rate, per 100,000 population	108.6	76.3	130.3	98.0
Estimated treatment coverage^b^	60	46	68	56
PPM notifications, baseline (2019)	4,160	2,258	4,843	11,261
PPM share of notifications, % of total	9.4	6.6	22.2	11.2

^a^Population-weighted average of regional TB prevalence rates from the 2017 to 2018 Viet Nam national TB prevalence survey, allocated by province per the survey’s regional strata.

^b^Estimated treatment coverage was calculated as the ratio of 2019 TB notifications to estimated TB incidence. Excluded provinces were those operating confounding community screening and PPM activities for which the impact could not be isolated. Full disaggregated provincial baseline characteristics are provided in Table F in S1 Appendix; PPM, public–private mix; TB, Tuberculosis.

The evaluation period spanned 38 quarters from Q1/2016 to Q2/2025, including 16 pre-intervention, 16 intervention and six post-intervention quarters. Launching in 2020, intervention deployment across the 15 provinces was staggered resulting in 112 province-quarters of intervention. There were dynamic changes in intervention intensity throughout implementation. Intensity was modeled as a continuous exposure of the population-adjusted rate of persons undergoing verbal symptom assessment per 100,000 population per quarter. The intervention lasted throughout the peak of the COVID-19 pandemic in Viet Nam (Q1-Q3/2021) until all activities ceased by year-end 2023 (Fig 1B) [20].

The contrast in baseline characteristics and dynamic intervention timing and intensity motivated the use of an analytic framework able to absorb province-level time-invariant differences via fixed effects, while robust to heterogeneity in intervention deployment.

### Intervention

**Fig 2:**
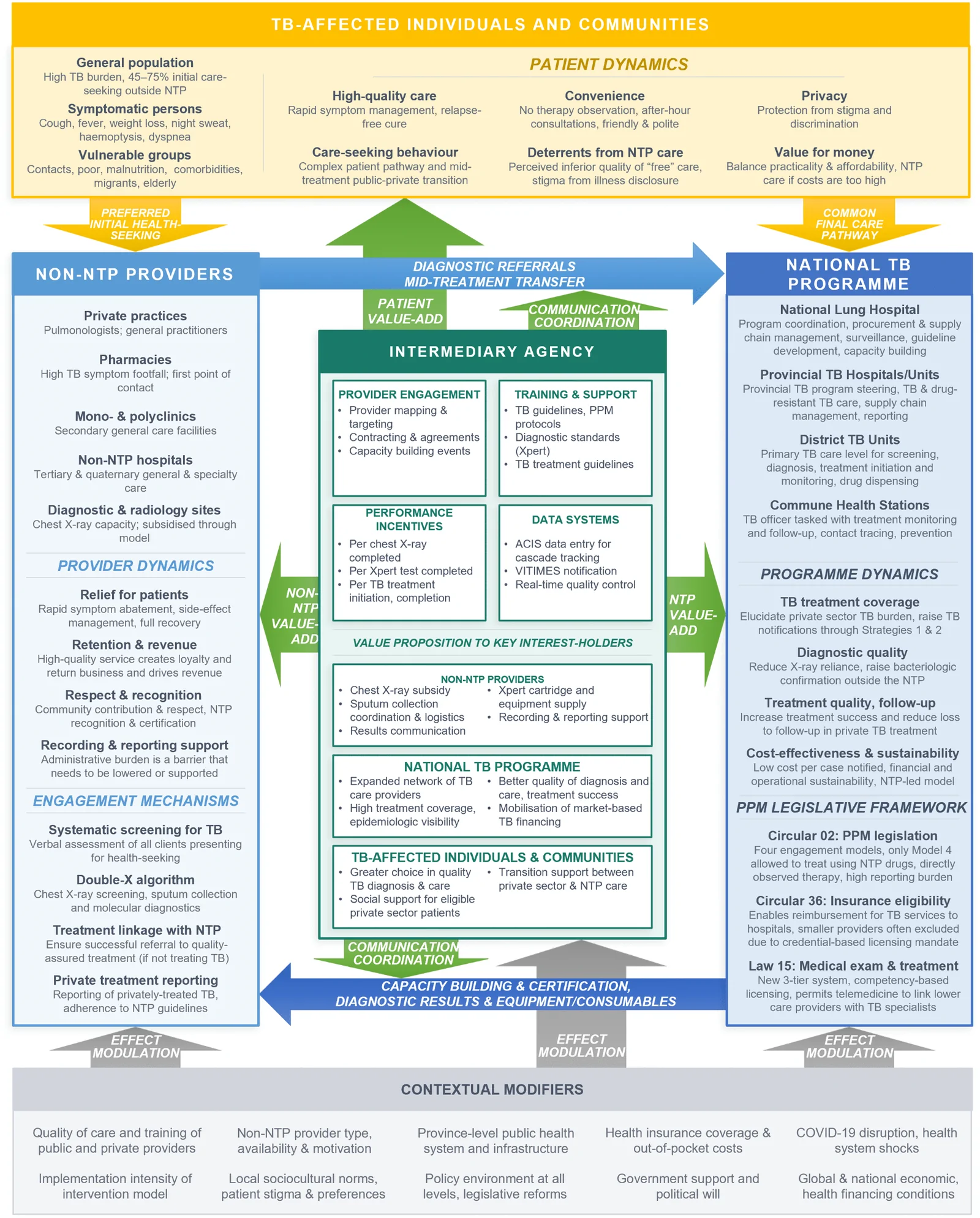
Conceptual framework for intermediary-facilitated public–private mix in Viet Nam. The framework maps the operational logic of the intervention across three interest-holder domains positioned around the intermediary agency. The domain of TB-affected individuals and communities captures the demand-side context. The non-NTP provider domain describes the provider groups and dynamics motivating participation. The NTP domain captures the program network, key priorities, and current legislative framework for PPM. The operational core comprises four interconnected functions and the value propositions for each interest-holder domain. Cross-cutting contextual modifiers are also shown; NTP, National TB Program; PPM, public–private mix; TB, tuberculosis.

The intervention was implemented through an intermediary agency that functioned as an operational bridge between TB-affected individuals and communities, non-NTP providers and the NTP (Fig 2). The intermediary identified, contracted, and supported private providers through training, supervision, and access to diagnostic services, while facilitating systematic screening, referral, and reporting of persons with presumptive TB. Providers participated through two strategies: referral-based diagnosis and linkage to treatment and notification at NTP facilities (Strategy 1), or diagnosis and treatment in the private sector with structured reporting to the NTP via the intermediary agency (Strategy 2).

Under Strategy 1, persons seeking care at participating healthcare providers were clinically assessed using WHO 4-symptom screen and referred for CXR screening on-site or at another radiography site. Persons with TB-related abnormalities on CXR received confirmatory molecular testing on Xpert MTB/RIF (Ultra) at an NTP-supported laboratory; those diagnosed with TB were linked to the NTP for any necessary reflex diagnostics and treatment initiation. Symptomatic persons without CXR abnormalities were eligible for conventional diagnostic tests such as smear microscopy and culture. Under Strategy 2, providers submitted quarterly demographic and clinical data of treatment initiations and outcomes to the intermediary agency. Providers collected sputum from persons with bacteriologically unconfirmed TB for molecular testing, if within two weeks of treatment initiation, and referred persons with rifampicin-resistant TB to the NTP. Agency staff managed quality control and entry of validated records into the NTP’s digital TB register, collected and transported samples for testing, communicated Xpert result to providers and supported treatment follow up. The latter included tracing treatment interruptions and facilitating transfer to the NTP, if cost was the reason for discontinuation. The intervention provided financial support to recommended disadvantaged patients and paid stipends to providers for each reported treatment initiation and outcome.

These activities were designed to result in improved detection and diagnostic quality; stronger engagement and active participation; higher quality of treatment and care; and greater transparency into private sector practices and notifications. Detailed procedures are in Section A.2 and C in S1 Appendix.

Data were obtained from digital systems managed by the intermediary agency and NTP. Study-specific systems included a bespoke customer relationship management tool that contained the master provider registries and into which agency staff recorded provider engagement activities and contracting data. NTP sources included the digital systematic screening tool, the TB-ACIS365 system (ACIS; acis365.vn), and the routine surveillance system, Vietnam Tuberculosis Information Management Electronic System (VITIMES) described elsewhere [21]. Demographic, symptom assessment, X-ray screening and diagnostic testing data were obtained from ACIS, while subnational and national TB notifications were exported from VITIMES. Cost data were obtained from study accounts.

### Outcomes

The primary outcome was the number of all forms of TB notifications, i.e., including bacteriologically confirmed and clinically diagnosed TB, per province per quarter recorded on VITIMES. Secondary outcomes included the number of PPM notifications per province per quarter recorded on VITIMES as an intermediate outcome, as well as provider engagement, recruitment, and participation across five provider types, and detection yield among health-seeking persons. Outcomes for the interrupted time-series analysis were quarterly national TB notifications, modeled as incidence rates with population offsets. Micro-costing outcomes were total program costs and marginal costs per additional TB notification from a funder perspective.

### Statistical analyses

No *a priori* sample size was calculated for the intervention or prospective analysis plan developed for the study. Based on the study objectives, initial analysis strategies included traditional two-way fixed-effect modeling and pragmatic approaches to measuring the effects of interventions in quasi-experimental designs [22–24]. However, the staggered deployment and variable intensity of the intervention necessitated the application of recently established, unbiased analytical methods for policy evaluations. While these novel methods enhance internal validity of the causal relationships, they rely on the validity of key assumptions. Study procedures were expanded to include testing of these assumptions as further described below.

We described characteristics of providers and health-seeking individuals in Tables G and H in S1 Appendix. We constructed a provider engagement cascade from enumeration to participation (completing ≥1 CXR referral) and contribution (detecting ≥1 person with TB), and two TB care cascades for each strategy. TB care cascades ranged from symptom assessment to treatment initiation for Strategy 1, and from private TB treatment initiation to successful completion for Strategy 2. All characteristic descriptions and cascades were stratified by five provider types: pharmacies, private practices, clinics, hospitals, and other.

We analyzed quarterly notification data of all forms of TB for intervention and control areas. To estimate the model’s causal effect, i.e., the Average Treatment effect on the Treated (ATT) on the primary outcome, we used an Extended Two-Way Fixed Effects (ETWFE) difference-in-differences framework designed to mitigate bias from staggered intervention rollout and effect heterogeneity. The framework specified province and calendar-quarter fixed effects to absorb time-invariant provincial differences and COVID-19 disruptions, and modeled the primary outcome using a Poisson distribution and population offset [25]. The ETWFE framework excluded the post-intervention period to avoid contamination by persistence effects. The staggered intervention deployment required a formal assessment of parallel pre-intervention trends rather than simple baseline equivalence. Event-study coefficients in the six quarters preceding implementation were jointly indistinguishable from zero (*p* = 0.619), satisfying the parallel-trend assumption, and showed no systematic upward trajectory approaching intervention launch. A wider pre-period test rejected equality to zero, reflecting modest deviations further from the intervention date (Fig B and Table A in S1 Appendix).

We fitted Poisson pseudo-maximum likelihood regression models with high-dimensional fixed effects, a population-offset, and standard errors clustered around province to quantify the dose–response relationship between intervention intensity and TB notifications, estimated as the incidence rate ratio, and to test robustness of the ETWFE estimates [26]. We evaluated post-intervention patterns for persistence/fade-out after Q4/2023 once all activities ceased and intensity returned to zero. Given heterogeneity in implementation timing, we assessed pooled and cohort-specific dose–response effects. Results were insensitive to alternative COVID-19 specifications, and cohort-specific models showed similar effects across rollout waves (Section A.6 in S1 Appendix).

We triangulated subnational estimates against national trends by fitting a complementary single-group national interrupted time-series Poisson model with population offset, linear and quadratic secular trend terms, seasonality, and a COVID-19 indicator (Section A.7 in S1 Appendix).

To assess whether the intervention’s effect on total TB notifications was driven by PPM notifications or via broader system effects, e.g., community-based active case finding, we repeated all aforementioned analyses using PPM notifications as the outcome of interest. The comparison group for these analyses was restricted to provinces operating traditional PPM models (models 1–4) to ensure comparability of reporting pathways. This analysis is reported as a sensitivity check on the attribution of the primary effect.

Lastly, we conducted a micro-costing analysis of intervention costs from a funder perspective. These costs capture the investments made through development assistance to generate project outputs and assume health system and patient expenditures as sunk costs. We described these project costs by major categories (Section A.8 in S1 Appendix) and calculated marginal costs per additional notification at provincial and national levels. At the provincial level, total project costs were divided by the product of the ATT times 112 province-quarters of intervention. At the national level, total project costs were divided by the product of additional TB notifications per year times four years of intervention. Costs were incurred in Viet Nam Dong (VNĐ) and translated to US Dollars (US$) at the average exchange rate during the treatment period (US$ 1 = VNĐ 23,716; Xe.com).

Hypothesis tests were two-tailed. A *p* < 0.05 was considered significant. Analyses were performed using Stata v17 (StataCorp; TX, USA). This study is reported as per the Strengthening the Reporting of Observational Studies in Epidemiology (STROBE) guideline (S1 Checklist). Anthropic’s Claude (version 4; Opus) and OpenAI’s ChatGPT (GPT-4) were used to improve the readability and language of the manuscript and to assist with debugging and annotating analysis code, which was verified by the authors. The tools were not used to design the study, analyze or interpret data, or draw scientific conclusions. We reviewed all output and take full responsibility for the content of this manuscript.

### Ethics

The study received ethical approval from the Hanoi University of Public Health (374/2023/YTCC-HD3, 31 July 2023) and WHO Western Pacific Region Ethics Review Committee (2019.22.VTN.5.STB, 16 May 2019). All individual-level data were de-identified prior to analysis.

## Results

### Provider recruitment and participation

Among 19,520 healthcare providers listed in the master registries, 91.8% (17,923/19,520) were eligible for engagement and recruitment, and 47.9% (8,587/17,923) were recruited to participate, of whom 36.7% (3,154/8,587) signed a contract. Among these, 26.0% (819/3,154) successfully referred or completed at least one CXR, which resulted in at least one person with TB detected for 65.0% (532/819) of providers, corresponding to 2.7% (532/19,520) of the total master registry (Fig D and Table I in S1 Appendix).

### TB care cascade by strategy

Under Strategy 1 (Table J in S1 Appendix), providers recorded 1,719,382 symptomatically assessed individuals, of whom 98.2% (1,688,471/1,719,382) received a CXR screen. About 10.6% (178,339/1,688,471) presented abnormalities suggestive of TB, of whom 81.2% (144,792/178,339) were tested using molecular diagnostics with a positivity of 17.8% (25,808/144,792). Among the 1,541,043 persons without a CXR or TB-related abnormalities, 1.0% (16,076/1,541,043) were tested due to other risk factors such as HIV co-infection, of whom 7.6% (1,221/16,076) were bacteriologically confirmed with TB. An additional 4,593 persons were clinically diagnosed for a total of 31,622 persons diagnosed with all forms of TB. Approximately 90.1% (28,502/31,622) had treatment initiation documented, of whom 79.6% (22,678/28,502) were at NTP-linked facilities and the remainder with a private provider.

Under Strategy 2 (Table K in S1 Appendix), 134 providers reported 12,839 private TB treatments, of whom 95.3% (12,231/12,839) passed the quality control process for official notification by the NTP, representing 30.0% (12,231/40,733) of total treatment initiations across both strategies. About 73.3% (9,414/12,231) of privately treated persons were reported to have successfully completed treatment.

### Effect on TB notifications

**Fig 3:**
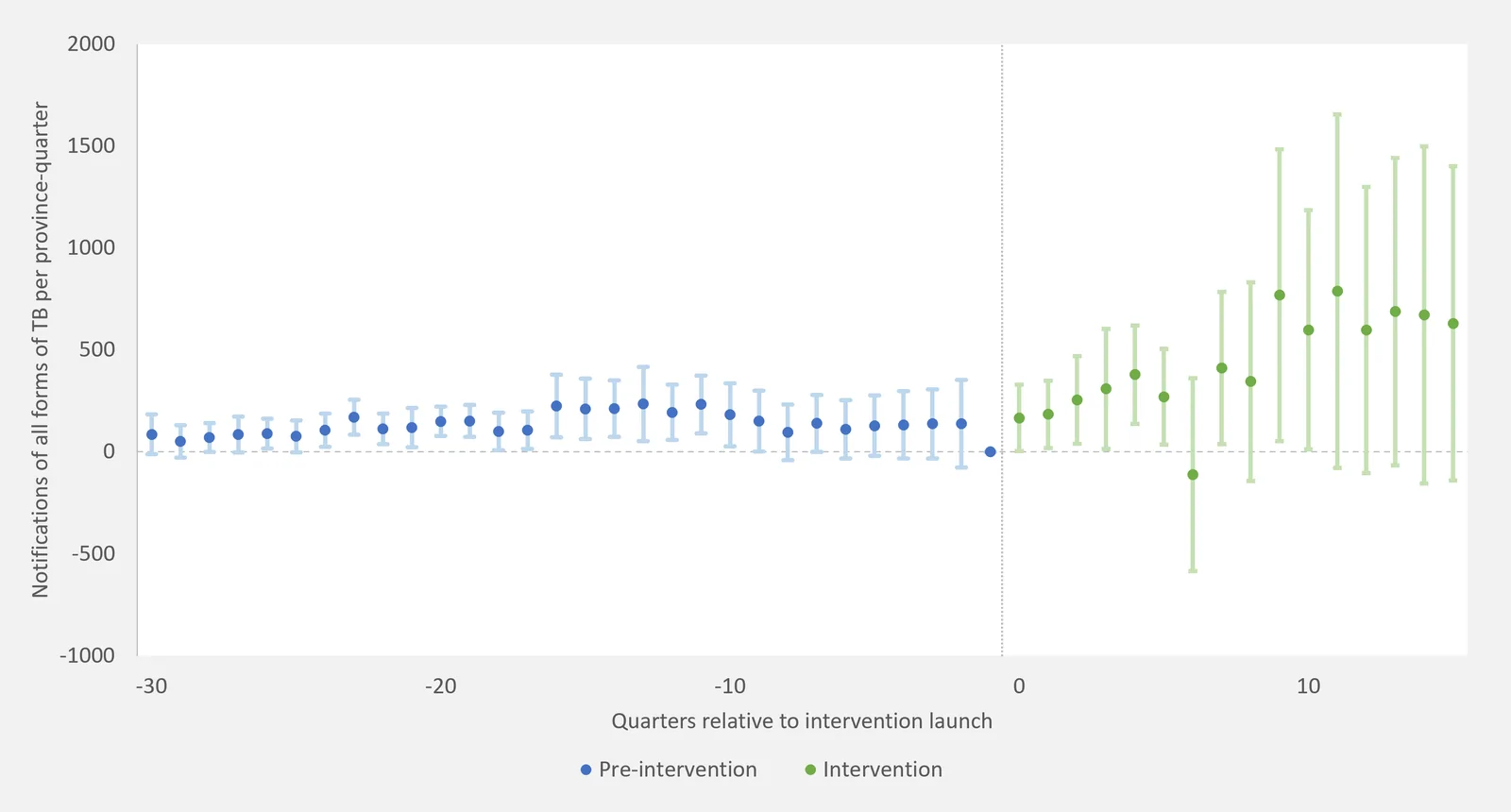
Event-study estimates of the effect of the intervention on quarterly tuberculosis notifications. Coefficients are from a group-time average treatment effects model comparing treated provinces with the full control area, restricted to treatment-period observations. Estimates are expressed as the change in TB notifications per province-quarter relative to the quarter immediately preceding intervention launch. Bars denote 95% confidence intervals. The joint pre-trend test for the six quarters preceding implementation was not significant (*p* = 0.62), supporting the parallel-trends assumption. A marked but transient decline is observed around period 6, coinciding with the peak of COVID-19-related service disruptions. TB, tuberculosis.

Aggregate mean quarterly notifications in the intervention and control areas during the pre-intervention period were 10,639 and 8,377, respectively. The intervention was associated with an average increase of 316.8 TB notifications per treated province-quarter (95% confidence interval [CI] [23.4, 610.2]; *p* = 0.034; Table 2). Event-study estimates (Fig 3) showed an immediate and sustained upward shift following implementation, with a transient decline during peak COVID-19 disruption in Q3/2021. Estimates were consistent across alternative specifications (Section A.5 in S1 Appendix).

**Table 2:** Estimates of intervention effects and dose–response association on tuberculosis and public–private mix notifications.

	TB notifications (95% CI)	*p*- value	PPM notifications^a^ (95% CI)	*p*- value
**Intervention effects** ^b^				
Average treatment effect on the treated (ATT), per province-quarter	316.8 [23.4, 610.2]	0.034	332.1 [137.5, 526.7]	0.001
Average 6-quarter pre-intervention baseline in intervention areas, per province-quarter	649.7 [644.0, 655.5]	n/a	83.0 [81.0, 85.1]	n/a
**Dose–response association** ^c^				
Intensity association (IRR), per 100,000 population	1.00014 [1.00002, 1.00025]	0.018	1.00070 [1.00034, 1.00105]	<0.001
Post-implementation residual indicator (IRR)	1.04818 [0.97463, 1.12729]	0.205	0.64657 [0.35024, 1.19360]	0.163

^a^The comparison group was restricted to provinces operating traditional PPM models (models 1–4); full diagnostics are in Section B.6 in S1 Appendix.

^b^Average treatment effect estimates were obtained from group-time average treatment effects models with province and quarter fixed effects, expressed as the change in TB notifications per treated province-quarter relative to the quarter immediately preceding intervention launch. Standard errors are clustered at the province level.

^c^Estimates were obtained from Poisson pseudo-maximum-likelihood regressions with province and quarter fixed effects and a population offset. Intensity is defined as the number of persons verbally assessed per 100,000 population per quarter. Standard errors were clustered at the province level. The post-implementation indicator equals 1 in quarters after screening intensity returned to zero, and tests for residual effects not explained by screening activity; ATT, Average Treatment effect on the Treated; CI, Confidence Interval; IRR, Incidence Rate Ratio; PPM, public–private mix; TB, Tuberculosis.

### Dose–response between intensity and TB notifications

In dose–response analyses (Table 2), each additional 100 verbal assessments per 100,000 population was associated with a 1.4% increase in notifications (incidence rate ratio [IRR] = 1.00014; 95% CI [1.00002, 1.00025]; *p* = 0.018). At the mean intensity of 513.6 per 100,000 population per quarter, this corresponds to a 6.7% marginal increase in notifications. No sustained residual effect was observed once implementation ceased (*p* = 0.205).

### Trend in national TB notifications

National interrupted time-series analyses provided convergent population-level evidence (Table L and Fig E in S1 Appendix). Trend-, seasonality-, and shock-adjusted increases in intensity were associated with a 3.5% increase in TB notifications per 100 verbal assessments per 100,000 population (IRR = 1.00035 per verbal assessment per 100,000 population; 95% CI [1.00018, 1.00052]; *p* < 0.001). Translating observed changes in intensity during implementation, model-based predictions indicate approximately 3,206 (95% CI [1,658, 4,763]) additional TB notifications per year nationally between 2020 and 2023.

### Intermediate outcome: PPM notifications

Examining PPM notifications as an intermediate outcome, the intervention was associated with an additional 332.1 PPM notifications per treated province-quarter (95% CI [137.5, 526.7]; *p* = 0.001; Table 2), comparable in magnitude to the effect on total TB notifications. The national interrupted time-series analysis showed a 24.2% increase in PPM notifications per 100 verbal assessments per 100,000 population (IRR = 1.00242; 95% CI [1.00190, 1.00294]; *p* < 0.001). Further detail, including event-study estimates, is provided in Section B.6 in S1 Appendix.

### Marginal costs per additional TB notification

Total funder costs were US$ 5,877,868 (Fig F in S1 Appendix). These consisted of 27.7% (US$ 1,044,220) in personnel costs, 38.8% (US$ 2,281,294) in one-time costs for meetings and trainings and procurement of diagnostic equipment and consumables, IT and communications, and 32.8% (US$ 1,930,743) in activities costs including incentives for providers and staff related to symptom assessment and screening, testing, treatment initiation and follow-up. Supervision costs, as well as administrative and one-time meeting costs, entailed 7.4% (US$ 434,548) and 3.2% (US$ 187,063) of total program costs, respectively. Marginal costs per additional notification in intervention provinces were US$ 165.66 (95% CI [US$ 86.01, US$ 2,242.78]) and at the national level US$ 458.21 (95% CI [US$ 308.52, US$ 886.29]).

## Discussion

Our study found that the systematic involvement of unengaged healthcare providers under the intermediary-facilitated PPM Model 5 strategy resulted in significant increases in TB notifications, which translated to a measurable association between the intervention and population-level TB notifications. The close correspondence between intermediate and primary effects, i.e., PPM and TB notifications, respectively, indicates that the intervention’s association with higher total TB notifications was driven by direct contributions through PPM rather than spillover from non-PPM pathways such as community-based active case finding. We further showed that these effects were linked to the intervention deployment and intensity and withstood peak COVID-19 disruptions.

Since the latest national prevalence survey in 2017–2018, Viet Nam’s NTP has been unable to reach >40% of persons with incident TB each year. Implemented in about 40% of the population, the intervention was associated with a sustained upward shift in TB notifications during the implementation window, which was reflected in national TB notification trends. Although the confidence interval was wide, the magnitude is programmatically meaningful, suggesting that a nationwide deployment of the intervention in Viet Nam could further expand national treatment coverage. Our results, based on modern causal inference methods reinforce past estimates based on traditional modeling, and strengthen the case for replication and scale-up of effective PPM models to combat the TB epidemic [27,28].

Implemented at scale, the intermediary agency engaged and maintained a large network of general and specialty care providers in our setting, who traditionally do not offer TB-related services. Through a small proportion of the master registry, this network substantially expanded national capacity for TB care and prevention, which detected and linked thousands of previously unknown people with TB to NTP care. Remarkably, less than 1% of providers in the master registry contributed about three-tenths of the total TB notification yield, suggesting that a refined intervention and targeted provider engagement could generate a large impact with modest efforts through the intermediary.

Going forward, the intervention could be further optimized to improve the model’s epidemiologic impact by focusing on systematic contact investigation of privately treated individuals, which was absent in the present iteration. Given the availability of effective TB preventive therapy, treatment guidelines of virtually all high burden countries include contact investigation for TB. However, this activity is often poorly implemented programmatically and virtually non-existent among non-NTP providers in Viet Nam. The feasibility of contact tracing and preventive therapy in the private sector remains an important knowledge gap.

Similar to many other high TB-burden countries, the COVID-19 pandemic severely affected health system capacity, curtailing treatment coverage and fueling excess mortality in Viet Nam [29]. National TB notifications declined by 22% year-on-year in 2021, which may result in an additional 22,000 TB episodes and 5,900 deaths by 2035 [30]. Our study’s scenario analyses of COVID-19 disruptions showed resilience of the intervention to such shocks and secular shifts, potentially as a result of sustained access to the Double-X strategy. This finding agrees with studies that have highlighted greater resilience in health systems with stronger public-private partnerships for health service delivery, particularly through the involvement of intermediary agencies, and continuity of care for TB [31].

Another theoretical aim of the model was to reduce morbidity and mortality through improved quality of diagnosis, treatment and follow-up. Previous studies have documented high rates of initial loss to follow-up, high reliance on clinical diagnosis, heterogeneity in treatment regimen and poor treatment outcomes in the private sector in Viet Nam [7,18]. In response, the intervention was designed to increase bacteriologic confirmation instead of reliance on radiographic evidence and promote adherence to national treatment guidelines with effective regimen construction and regular follow-up. In combination, the intervention achieved equivalent rates of bacteriologic confirmation, detection of drug resistance, and linkage to treatment to those reported by the NTP [32]. Privately treated clients under Strategy 2 were also able to receive treatment adherence and socioeconomic support, but still paid out-of-pocket for their medication and consultations. This may have contributed to a treatment success rate that was higher than previously measured in Viet Nam’s private sector [7,15], but still well below the national average (89%) [33]. Further research is needed to evaluate the mechanisms by which the intermediary agency model can contribute to greater treatment success.

Suboptimal private sector treatment outcomes represented a key challenge in engaging the NTP. Specifically, the inclusion of private TB treatment reports in national notifications from PPM Model 5 was a major implementation barrier. For the NTP, it posed a threat to deleteriously affect program performance. On the provider side, it was necessary to strike the right balance in reporting burden that meets government reporting requirements, while remaining within the administrative tolerance thresholds for participation. This included the need to report treatment outcomes linked with the expectation of upholding public sector treatment success among private clientele. Reconciling these tensions required substantial negotiation by the intermediary with the NTP, technical agencies and non-program providers. In this regard, the NTP’s compromise to accept lower success rates in the near-term in exchange for higher notifications and the opportunity to build capacity for improved TB care in the long-term, and provider willingness to accept greater administrative burdens were critical enablers for the success of the model.

An intervention’s scalability depends heavily on its affordability within existing health system structures. In Viet Nam, many formal healthcare providers are registered for health insurance reimbursement, so that the required investments to scale the model are reflected by the study’s reported funder-related costs. These costs were consistent with costs reported for PPM interventions from other high-burden settings (mean: US$ 221; median: US$ 481; *n* = 35 projects) [34]. Nevertheless, routine program scale-up processes as well as costs additionally incurred and potentially saved to programs and patients through a full health economic analysis comprise areas for further research to strengthen the current evidence of engaging the private sector. In 2023, the NTP began to institutionalize the intermediary model and key functions such as provider engagement, referral coordination, and reporting in five pilot provinces, leveraging the integration of TB financing into Viet Nam’s health insurance mechanism. However, the scale-up faced early implementation barriers. First, instrumental funder-related costs such as provider incentives and patient cash transfers were not included in statutory reimbursement packages. Second, provincial TB programs lacked the necessary human resources to sustain the model’s intensive provider engagement model. Critically, this effort was disrupted by the country’s 2025 administrative reform. Thus, critical evidence gaps on the model’s replicability, scalability and sustainability under routine program operations remain.

Carried by national and international momentum to end TB, more ambitious approaches such as community-wide screening (CWS) have attracted much recent attention [35]. A recent modeling study estimated that reaching a prevalence of 50 per 100,000 in Viet Nam through CWS would require front-loaded investments of US$ 1 billion, or US$ 161 million annually for eight years [36]. Given disruptions caused by the rationalization in global health financing and rising economic pressures from geopolitical tensions, however, the urgency to adopt such ambitious public health endeavors may have lessened in many resource-limited settings [37]. The intermediary-facilitated PPM model could serve as a viable interim solution to ensure the momentum towards achieving end TB ambitions is sustained [38].

Our results are in close alignment with calls for integration of TB and primary healthcare as a pathway to achieving universal health coverage, while offering a model for operationalizing these headline strategies [39,40]. Greater health system integration constitutes a critical component of the emergent whole-of-society approach [41], which proved effective in controlling the COVID-19 pandemic [42]. Particularly the effective repurposing of existing infrastructure for TB and other vertical programs for COVID-19 control demonstrated the need for greater intersectoral collaboration and financing [43]. Beyond TB and COVID-19, however, this approach also represents a key opportunity for improved epidemic control, population health and health equity overall [44].

Our study has several limitations. By its quasi-experimental nature and the purposive selection of high-burden provinces for the intervention, the study is subject to residual confounding from unobserved political and administrative factors for which the intervention area was selected. For example, the project specifically targeted provinces with progressive health authorities keen to reduce their TB burden, who leveraged their political influence to promote non-NTP provider engagement under Viet Nam’s unitary political environment. Moreover, urban areas tend to offer better healthcare infrastructure, which attracts greater health-seeking compared to rural and remote provinces. These structural differences likely led to an overestimation of the intervention’s treatment effect and notification trends. Similarly, while the intermediate analysis using PPM notifications strengthens the causal interpretation, it also means that scaling the model to settings with structurally different private-sector notification pathways may not produce equivalent effects on total TB notifications. We attempted to mitigate this bias analytically by modeling province and quarter fixed effects, assessing the parallel-trend assumption and conducting multi-factorial diagnostic and robustness tests. Further, the provider engagement cascade exhibited steeps drops between the number of providers in the master registries and those actively participating and positively contributing to TB notifications. Similarly, we observed substantial deviations in the distribution of provider types in the master registries, signing participation contracts, actively participating and contributing to case detections by typology. However, given the large volume of providers and scale of the intervention, provider engagement barriers and root causes were unavailable and represent key areas for future implementation research.

The intermediary agency model was effective in engaging a large number of healthcare providers outside of the NTP network. The engagement of these providers generated high rates of screening and TB diagnoses with signs of improvement in private sector quality of diagnosis and treatment. The intervention was associated with a measurable positive increase in subnational and national TB notifications. Given its resilience to shocks, such as the COVID-19 pandemic, and relative affordability in times of contracting global health financing, intermediary-facilitated PPM models may serve as a viable strategy to sustain momentum in global efforts to end TB, while also serving as a blueprint to operationalize primary healthcare integration.

### Disclosure on AI use

Anthropic’s Claude (version 4; Opus) and OpenAI’s ChatGPT (GPT-4) were used to improve the readability and language of the manuscript and to assist with debugging and annotating analysis code, which was verified by the authors. The tool was not used to design the study, analyze or interpret data, or draw scientific conclusions. We reviewed all output and take full responsibility for the content of this manuscript.

## Supporting information

S1 Appendix Supplementary Information with additional text, tables, and figures. (DOCX)

S1 Checklist STROBE checklist. The STROBE checklist is reproduced from von Elm E, Altman DG, Egger M, Pocock SJ, Gøtzsche PC, Vandenbroucke JP; STROBE Initiative. The Strengthening the Reporting of Observational Studies in Epidemiology (STROBE) statement: guidelines for reporting observational studies. PLoS Med. 2007;4(10):e296. https://doi.org/10.1371/journal.pmed.0040296. Licensed under CC BY 4.0 (https://creativecommons.org/licenses/by/4.0/). Study-specific reporting locations have been added to the original checklist.
(DOCX)

S1 Code Stata code used for the analysis. (DOCX)

## Abbreviations

ATT: Average Treatment effect on the Treated
CI: confidence interval
CWS: community-wide screening
CXR: Chest X-ray
ETWFE: Extended Two-Way Fixed Effects
IRR: incidence rate ratio
NTP: national TB programs
PPIA: Private Provider Interface Agencies
PPM: public–private mix
VITIMES: Vietnam Tuberculosis Information Management Electronic System
WHO: World Health Organization
