# Supplementary material for: Evaluation of the tuberculosis notification impact from an intermediary-facilitated public–private mix intervention in Viet Nam: A population-level quasi-experimental study
Source: PLoS Med. 2026 Sep 23;23(9):e1005144. doi: 10.1371/journal.pmed.1005144 (PMC13600524; doi:10.1371/journal.pmed.1005144)
Supplement: Supplementary file 1 [file pmed.1005144.s001.docx]

Appendix

Table of Contents

[Section A Supplementary Methods	3](#_Toc230944402)

[A.1: Cohort definitions and eligibility	3](#_Toc230944403)

[A.2: Overview of intervention description and implementation fidelity	3](#_Toc230944404)

[A.3: Data systems and variable construction	4](#_Toc230944405)

[A.4: Causal inference framework	5](#_Toc230944406)

[A.5: Extended two-way fixed-effects modeling diagnostics	5](#_Toc230944407)

[A.6: Dose–response modeling diagnostics	7](#_Toc230944408)

[A.7: National interrupted time-series analysis	9](#_Toc230944409)

[A.8: Micro-costing methods	9](#_Toc230944410)

[A.10: Provincial baseline characteristics for intervention, control and excluded provinces.	11](#_Toc230944411)

[Section B Supplementary Results	15](#_Toc230944412)

[B.1: Descriptive statistics of healthcare providers and verbally assessed individuals.	15](#_Toc230944413)

[B.2: Provider engagement cascade.	19](#_Toc230944414)

[B.3: Tuberculosis care cascade by strategy.	21](#_Toc230944415)

[B.4: National interrupted time-series analysis model results.	23](#_Toc230944416)

[B.5: Micro-costing results by primary and sub-categories.	25](#_Toc230944417)

[B.6: Intermediate outcome analysis: PPM notifications	26](#_Toc230944418)

[Section C Supplementary Protocol	29](#_Toc230944419)

[C.0: About this protocol	29](#_Toc230944420)

[C.1: Strategy 1 – Diagnostic referral through engaged non-NTP providers	30](#_Toc230944421)

[1. Overview	30](#_Toc230944422)

[2. Provider identification and engagement	30](#_Toc230944423)

[3. Symptom assessment and referral	31](#_Toc230944424)

[4. CXR screening and bacteriological confirmation	31](#_Toc230944425)

[5. Result return and provider communication	32](#_Toc230944426)

[6. Treatment initiation, notification, and follow-up	33](#_Toc230944427)

[C.2: Screening at non-NTP hospitals	34](#_Toc230944428)

[7. Scope and entry points	34](#_Toc230944429)

[8. Screening procedures	34](#_Toc230944430)

[9. Sputum collection and transport	34](#_Toc230944431)

[10. Diagnostic interpretation and treatment linkage	34](#_Toc230944432)

[C.3: Occupational health screening	35](#_Toc230944433)

[11. Scope and engagement	35](#_Toc230944434)

[12. Screening modalities	35](#_Toc230944435)

[*12.1 Modality A: Symptom-driven referral*	35](#_Toc230944436)

[13. Sputum handling and treatment linkage	35](#_Toc230944437)

[C.4: Strategy 2 – Notification of privately treated TB	36](#_Toc230944438)

[14. Scope and rationale	36](#_Toc230944439)

[15. Reporting procedures	36](#_Toc230944440)

[16. Verification of disclosed clients	36](#_Toc230944441)

[17. Notification through VITIMES	37](#_Toc230944442)

[18. Patient monitoring and treatment support	37](#_Toc230944443)

[C.5: Data systems and quality assurance	38](#_Toc230944444)

[19. Data systems	38](#_Toc230944445)

[20. Voucher reconciliation and digitization	38](#_Toc230944446)

[21. Performance-based incentive structure	38](#_Toc230944447)

[22. Reporting cadence	38](#_Toc230944448)

[C.6: Staffing and governance	39](#_Toc230944449)

[23. Staffing structure	39](#_Toc230944450)

[24. Role of the PPM Manager	39](#_Toc230944451)

[25. Role of the PPM Officer	39](#_Toc230944452)

[26. Reporting structure	40](#_Toc230944453)

[C.7: Adaptations during the implementation period	41](#_Toc230944454)

[27. COVID-19 adaptations	41](#_Toc230944455)

[28. Expansion of provider categories and intervention components over time	41](#_Toc230944456)

[29. Operational refinements	41](#_Toc230944457)

# Section A Supplementary Methods

## A.1: Cohort definitions and eligibility

**Primary and secondary study cohorts, inclusion and exclusion criteria, provider typology, reasons for non-participation, and handling of unreachable or non-consenting providers.**

The study included a primary and a secondary study cohort. With respect to the primary cohort, the sampling frame encompassed all formal healthcare providers located in the intervention provinces. We included licensed providers from master registries shared by provincial health authorities. Eligible providers included pharmacies, private solo practices, mono- and polyclinics, public and private hospitals, radiography centers, laboratories, and occupational health screening providers. Providers were required to hold a valid operating license and to offer direct healthcare services. Providers were excluded if they could not be contacted after repeated attempts, declined to meet with agency staff, or declined to sign a collaboration agreement. Providers participating exclusively in unrelated TB case-finding initiatives were excluded to avoid contamination. Participating providers were categorized into five major types for stratified analyses of engagement, contribution, and diagnostic yield.

The secondary cohort included all health-seeking persons reported by participating private providers. All persons with a verbal or chest x-ray (CXR) screening result were included. These persons belonged to one of two possible cohorts. The first cohort (Diagnostic Referral cohort) entailed those screened and referred from a PPM provider to receive diagnosis and treatment from the National TB Control Programme (NTP). This cohort included individuals who may have interacted with multiple PPM Model 5 providers to complete their screening and diagnostic algorithm. The second cohort consisted of persons reported as receiving private treatment from a non-NTP provider (Private Treatment cohort). We excluded persons in the Diagnostic Referral cohort who transitioned to a Circular 02 PPM facility throughout their care pathway. We also excluded persons in the Private Treatment cohort who had already been notified by the NTP prior to shifting to private treatment for the current episode of TB.

## A.2: Overview of intervention description and implementation fidelity

**Detailed description of intermediary agency procedures, provider recruitment, training, incentive structures, diagnostic algorithms, data flows, and quality assurance for Strategies 1 and 2.**

The PPM intervention was implemented through an intermediary agency that functioned as an operational bridge between the NTP and non-program healthcare providers. The agency maintained master registries of healthcare provider obtained from provincial health authorities and systematically contacted eligible providers to present the intervention and assess willingness to participate. Providers who agreed to collaborate signed standardized agreements defining roles, responsibilities, reporting requirements, and incentive structures. All providers agreeing to participate signed collaboration agreements tailored to an agreed set of responsibilities. Participating providers received a one-day training covering national TB diagnostic and treatment guidelines, referral pathways, data reporting procedures, and quality assurance requirements. Providers elected to participate via one of two strategies: referral-based diagnosis and linkage to the NTP (Strategy 1), or private TB treatment with structured reporting to the NTP (Strategy 2). Intervention procedures, incentive structures, and reporting workflows were standardized across provinces but allowed flexibility in provider participation intensity.

Specifically, providers unable or unwilling to privately treat TB participated via Strategy 1, whereby health-seeking persons were clinically evaluated and referred for CXR screening on-site or at another radiography site. Persons with TB-related abnormalities on CXR received confirmatory molecular testing on Xpert MTB/RIF (Ultra) at an NTP-supported laboratory. All persons diagnosed with TB were linked to the NTP for any necessary reflex diagnostics and treatment initiation. Agency staff managed specimen collection and transport, if needed, and telephonically tracked individuals diagnosed with TB to minimize pre-treatment loss to follow-up. The intervention paid financial incentives to providers for screening, testing and successful treatment linkage, and per-test stipends to Xpert laboratories. The NTP provided diagnostic consumables and placed equipment at high-volume facilities.

Providers offering private TB treatment were engaged to participate via Strategy 2, whereby providers submitted quarterly demographic and clinical data of treatment initiations and outcomes to the intermediary agency. Providers collected sputum from persons with bacteriologically-unconfirmed TB for molecular testing, if within two weeks of treatment initiation. Persons with rifampicin-resistant TB were transferred to the NTP. Providers managed follow-up until treatment completion. Providers could also recommend economically disadvantaged persons with TB to the intermediary agency for provision of social support. Agency staff managed quality control and entry of validated records into the NTP’s digital TB register, collected and transported samples for testing, communicated Xpert result to providers and supported treatment follow up. The latter included tracing treatment interruptions and facilitating transfer to the NTP, if cost was the reason for discontinuation. The intervention provided financial support to recommended disadvantaged patients and paid stipends to providers for each reported treatment initiation and outcome.

In terms of mechanisms of action, the intervention included a number of design features that served as key enablers under the real-world constraints of our setting, including free access to molecular diagnostics as part of the Double-X algorithm; financial and non-financial incentives; patient follow-up and social support; and data management. These mechanisms were designed to results in the following public health outcomes: improved detection and diagnostic quality; stronger engagement and active participation; higher quality of treatment and care; greater transparency into private sector practices and notifications.

Access to molecular diagnostics was restricted to the NTP at the launch of the intervention. While Xpert became commercially available throughout the doctoral project, market prices were a multitude of public health pricing. As such, throughout the project the NTP provided diagnostic consumables for molecular testing and placed equipment with key providers. For PPM providers with smaller volumes, sample collection and transport were facilitated by the intermediary and external shipping providers.

Providers and laboratories also received process- and outcomes-based financial incentives as well as non-financial incentives. Non-financial incentives included capacity building and technical assistance from the NTP as well as operational support through continuous technical and occasional hands-on support by agency staff. Additionally, providers engaged under Strategy 2 were able to notify the intermediary agency of particularly destitute clients in need of social support. Following a formalized validation procedure, eligible clients received differentiated financial support throughout treatment.

Financial incentives included payments for accurate reporting of CXR referrals and results; diagnostic sputum testing on Xpert; successful treatment linkage; notification of private TB treatment, and treatment outcome reporting. In the Vietnamese setting, formal contracting was mandatory with bespoke templates for different stakeholders, task mixes and associated incentives, which provided legal reassurance to participating providers. Payments were made monthly with statutory income tax withholding and reporting managed by the intermediary agency. Payments were calculated based on officially recorded activities and outputs.

Operationally, the intermediary agency undertook a number of process optimization activities. Providers were followed up periodically, including for all providers with expiring collaboration contracts, which were renewed, if so desired by the provider. Unresponsive providers and those declining to participate were recorded as such in the customer relationship management system. Health-seeking individuals not completing the next step in the TB care cascade were contacted by the provider and agency staff.

The intermediary agency was held accountable by the NTP to ensure reliable surveillance was in place that enabled accurate recording and reporting of official notification and treatment outcomes with well-document paper-based and electronic audit trails. As such, implementation fidelity was monitored through weekly reporting dashboards, automated data validation checks, and routine supervision by agency field staff. Deviations from protocol, including delayed reporting or incomplete records, were flagged for follow-up. Unsuccessful follow-up efforts were marked and reported as loss to follow-up in the care cascade.

## A.3: Data systems and variable construction

**Description of study-specific digital systems, NTP surveillance platforms, data linkage procedures, construction of screening intensity variables, COVID-period indicators, and population offsets.**

Study-specific data were captured using a bespoke digital customer relationship management system, SPECTRUM, in which agency staff recorded provider enumeration, engagement attempts, contracting status, and activity metrics.

For Strategy 1, screening, referral, diagnostic, and treatment data were recorded either directly by providers or entered by agency staff from standardized digital submissions. For Strategy 2, providers shared patient diagnosis and treatment information adherent to NTP reporting standards for entry into VITIMES. Agency staff verified data quality using automated software procedures and manual spot checks prior to entry, and generated weekly progress reports. VITIMES data were used to construct aggregate quarterly notification counts in the intervention and control areas as shown in Fig A below.

Intervention intensity was defined as the number of verbally screened individuals per quarter, normalized to population size (screenings per 100,000 population). COVID-19 disruption periods were identified using calendar-quarter indicators corresponding to national public health restrictions. Population offsets were derived from official provincial and national population estimates.

**Fig A: Quarterly aggregate tuberculosis notifications in intervention and control areas, 2016–2025.**

The figure shows mean quarterly notifications of all forms of tuberculosis in the PPM Model 5 intervention (PPM5; green) and control (blue) areas during the (Q1/2016 to Q4/2019), intervention (Q1/2020 to Q4/2023) and post-intervention (Q1/2024 to Q2/2025) periods. Vertical dashed lines denote program phase boundaries; a pronounced decline in notifications is observed in both groups during 2021, coinciding with peak COVID-19-related disruption to TB services.

Quarterly notificaitons of all forms of TB ('000s)

|  | PPM5 | Control |
| --- | --- | --- |
| 2016 1 | 10651 | 9052 |
| 2016 2 | 11190 | 10117 |
| 2016 3 | 10654 | 10228 |
| 2016 4 | 11257 | 10212 |
| 2017 1 | 9974 | 9197 |
| 2017 2 | 10670 | 10875 |
| 2017 3 | 10670 | 10875 |
| 2017 4 | 10041 | 10182 |
| 2018 1 | 9524 | 8825 |
| 2018 2 | 10405 | 10061 |
| 2018 3 | 10451 | 10251 |
| 2018 4 | 10642 | 9606 |
| 2019 1 | 10354 | 8409 |
| 2019 2 | 11006 | 9846 |
| 2019 3 | 11682 | 10188 |
| 2019 4 | 11054 | 9220 |
| 2020 1 | 10395 | 8384 |
| 2020 2 | 9967 | 7978 |
| 2020 3 | 10826 | 10198 |
| 2020 4 | 11460 | 10153 |
| 2021 1 | 11612 | 7903 |
| 2021 2 | 10244 | 7510 |
| 2021 3 | 4575 | 4990 |
| 2021 4 | 9173 | 6737 |
| 2022 1 | 9537 | 6661 |
| 2022 2 | 11958 | 9880 |
| 2022 3 | 11467 | 9765 |
| 2022 4 | 11968 | 9091 |
| 2023 1 | 11124 | 8226 |
| 2023 2 | 11587 | 8627 |
| 2023 3 | 11779 | 9250 |
| 2023 4 | 11726 | 9279 |
| 2024 1 | 10091 | 8330 |
| 2024 2 | 10767 | 8865 |
| 2024 3 | 10767 | 8865 |
| 2024 4 | 11027 | 10060 |
| 2025 1 | 9774 | 8803 |
| 2025 2 | 10387 | 9533 |

## A.4: Causal inference framework

**Formal specification of the extended two-way fixed-effects difference-in-differences estimator, identifying assumptions, handling of staggered adoption, and rationale for excluding post-treatment periods.**

To estimate the average treatment effect on the treated (ATT), we used an extended two-way fixed-effects difference-in-differences framework adapted for staggered intervention rollout and heterogeneous treatment effects. The model included province fixed effects to absorb time-invariant provincial characteristics and calendar-quarter fixed effects to capture common shocks, including the COVID-19 pandemic.

The estimand of interest was the average number of additional TB notifications per treated province-quarter attributable to the intervention. A Poisson mean structure with a population offset was used to facilitate interpretation on the count scale. Post-treatment periods were excluded from the ATT estimation to avoid contamination by dynamic or persistence effects.

Identification relied on the assumption of parallel trends in the absence of intervention, conditional on fixed effects. This assumption was assessed using event-study diagnostics described below.

## A.5: Extended two-way fixed-effects modeling diagnostics

**Formal specification of the extended two-way fixed-effects difference-in-differences estimator, identifying assumptions, handling of staggered adoption, and rationale for excluding post-treatment periods.**

Identification relied on the assumption of parallel trends in the absence of intervention, conditional on fixed effects. To assess the parallel trends assumption, we estimated event-study models and conducted joint Wald tests of pre-treatment coefficients. Because the dataset spans a long pre-intervention period, we pre-specified a local diagnostic window focusing on the six quarters immediately preceding implementation (t−6 to t−2), corresponding to the final 18 months before exposure. This approach prioritizes identification of differential trends immediately prior to treatment, rather than distant baseline differences. Joint tests were performed on these near-term leads using cluster-robust standard errors at the province level. These joint tests of event-time coefficients in the six quarters preceding implementation showed no statistically significant deviations from zero and no systematic upward trend approaching treatment (joint p=0.62).

When extending the pre-treatment window further from the intervention date, joint tests rejected equality to zero, reflecting modest deviations at distant leads. Given the long pre-period and potential structural changes unrelated to the intervention, the primary analysis focused on a trimmed pre-treatment window proximate to implementation. Sensitivity analyses using alternative lead windows for the main model specification produced similar post-treatment estimates.

**Table A: Results of parallel-trend assumption joint tests by pre-treatment time window.**

|  | **df** | **χ²** | ***p*-value** |
| --- | --- | --- | --- |
| 8 quarters (t−8 to t−2) | 7 | 15.56 | **0.0295** |
| 6 quarters (t−6 to t−2) | 5 | 3.53 | **0.6188** |
| 4 quarters (t−4 to t−2) | 3 | 3.10 | **0.3763** |

Each row reports the χ² test of the null hypothesis that all included pre-intervention coefficients are jointly equal to zero for the indicated pre-intervention window, with degrees of freedom corresponding to the number of leads tested. Results illustrate sensitivity of the joint test to the length of the pre-intervention window.

Nevertheless, to assess the impact of distant lead violations in the parallel-trends assumption underlying the difference-in-differences design, we conducted pre-intervention slope comparisons between treated provinces and each potential control province individually using pre-2020 data only. For each control province, we estimated a Poisson regression of quarterly TB notifications including a linear time trend and an interaction between time and a province-specific control indicator, adjusting for calendar-quarter fixed effects and population exposure. A non-significant interaction term (p>0.10) was interpreted as evidence that pre-intervention trends did not differ materially from those in treated provinces.

**Fig B: Distribution of parallel trend test *p*-values.**

The figure shows *p*-values from the pre-intervention slope comparisons between treated provinces and each potential control province individually using pre-2020 data only using a Poisson regression of quarterly TB notifications.

Therein were eight provinces that exhibited parallel pre-intervention trends and comprised the restricted control area (Population: 7.2 million; TB: 5,424; PPM: 549 [10.1%]). In sensitivity analyses, we restricted the control group to provinces meeting this criterion and compared results with the full-sample estimates.

**Fig C: Scenario analyses of full versus restricted control & including versus excluding the post-intervention period.**
The figures show event-study plots for A) full control excluding post-treatment period; B) Full control including post-treatment period; C) Restricted control excluding post-treatment period; D) Restricted control including post-treatment period. All four scenarios showed consistent patterns of pre-intervention trends centered near zero, with a substantial increase immediately after the launch of the intervention, a transient decline in period 6 at peak COVID-19 disruptions and large standard errors in distant lags due to the smaller sample size in those later periods.

| **A**  | **B**   |
| --- | --- |
| **C** | **D**   |

We retained the full control group as the primary comparison because it maximized statistical precision and improved efficiency without materially altering the magnitude or direction of the estimated treatment effect. Results using a restricted control set were directionally consistent but less precise, and did not substantively change interpretation. Given the absence of differential trends in the six quarters immediately preceding implementation, the broader comparison group was considered appropriate for the main analysis.

## A.6: Dose–response modeling diagnostics

**Poisson pseudo–maximum likelihood model specification, justification for functional form, cohort-specific intensity models, and robustness to alternative intensity scaling and COVID-period treatment.**

Dose–response relationships between intervention intensity and TB notifications were estimated using Poisson pseudo–maximum likelihood regression with high-dimensional fixed effects (PPMLHDFE). Models included province and calendar-quarter fixed effects, a population offset, and standard errors clustered at the province level.

Intervention intensity was modeled as a continuous exposure variable defined as screenings per 100,000 population per quarter.

To evaluate the main specification (1) for post-intervention persistence or fade-out (2), we included indicators for post-implementation periods after intervention cessation while intensity returned to zero.

Robustness comparison between examined sensitivity to a restricted control sample (3) as defined in Section A-E.

Cohort-specific intensity models (4) were estimated and demonstrated heterogeneity in the marginal association between screening intensity and TB notifications across rollout waves. Early and mid-stage cohorts showed positive and statistically significant associations, whereas later cohorts exhibited attenuated and less precise estimates, consistent with declining marginal returns and post-scale-up dynamics. Importantly, no single cohort accounted for the pooled dose–response effect, supporting the robustness of the main estimates.

Results were robust to alternative variance estimators, including heteroskedasticity-robust standard errors.

Estimates were stable across all specifications as shown below.

**Table B: Robustness analyses to alternative intensity scaling.**

|  | **(1) Full controls  with fade-out  (main specification)** | **(2) Full controls, no post-treatment** | **(3) Restricted controls, no post-treatment** | **(4) Cohort-specific model with fade-out** |
| --- | --- | --- | --- | --- |
| Treatment intensity (pooled)  (95% CI)  *p*-value | 1.00014  [1.00002, 1.00024]  0.018 | 1.00013  [1.00002, 1.00024]  0.021 | 1.00014  [1.00002, 1.00026]  0.028 | -- |
| Q1/2020 cohort  (95% CI)  *p*-value | -- | -- | -- | 1.00021  [1.00010, 1.00030]  <0.001 |
| Q4/2021 cohort  (95% CI)  *p*-value | -- | -- | -- | 1.00008  [1.00000, 1.00015]  0.048 |
| Q1/2022 cohort  (95% CI)  *p*-value | -- | -- | -- | 0.99987  [0.99975, 0.99998]  0.024 |
| Q2/2022 cohort  (95% CI)  *p*-value | -- | -- | -- | 1.00008  [1.00004, 1.00011]  <0.001 |
| Q3/2022 cohort  (95% CI)  *p*-value | -- | -- | -- | 1.00010  [1.00000, 1.00019]  0.045 |
| Q2/2023 cohort  (95% CI)  *p*-value | -- | -- | -- | 1.00003  [0.99985, 1.00021]  0.727 |
| Q3/2023 cohort  (95% CI)  *p*-value | -- | -- | -- | 1.00018  [0.99995, 1.00041]  0.122 |

Poisson pseudo–maximum likelihood regression models with province and quarter fixed effects and population offsets estimating pooled and cohort-specific associations between screening intensity and TB notifications: (1) with fade-out specification includes a term for the post-intervention period; (2) no post-treatment regresses solely against the COVID-19-adjusted treatment intensity; (3) restricted controls include only provinces meeting pre-specified parallel-trend criteria as described in Section A-E as the counterfactual; and (4) the cohort-specific model includes interaction of intensity with seven treatment cohort indicators indicating intervention launch quarters. Estimates show heterogeneity in magnitude across rollout waves, but no evidence that pooled results are driven by a single cohort.

**Table C: Robustness analyses to alternative variance estimators for PPMLHDFE estimates.**

|  | **(1) Province-clustered standard errors (main specification)** | **(2) Robust standard errors** |
| --- | --- | --- |
| Treatment intensity (pooled)  (95% CI)  *p*-value | 1.00013*  [1.00002, 1.00024]  0.021 | 1.00013*  [1.00008, 1.00018]  <0.001 |

Notes: Estimates from Poisson fixed-effects models using province-clustered standard errors (1, main specification) and heteroskedasticity-robust standard errors (2). Point estimates are identical across specifications; clustered standard errors are larger, indicating conservative inference under the preferred clustering approach.

**Table D: Robustness analyses to alternative treatments of COVID-19 disruptions.**

|  | **(1) COVID-19-adjusted intensity with fade-out**  **(main specification)** | **(2) COVID-19 quarters (Q1-Q3/ 2021) excluded with fade-out** | **(3) Unadjusted intensity with COVID-19 indicator with fade-out** | **(4) Unadjusted intensity with COVID-19 interaction term with fade-out** |
| --- | --- | --- | --- | --- |
| Treatment intensity (pooled)  (95% CI)  *p*-value | 1.00014  [1.00002, 1.00025]  0.018 | 1.00015  [1.00003, 1.00026]  0.014 | 1.00016  [1.00004, 1.00028]  0.007 | 1.00015  [1.00003, 1.00027]  0.013 |

Estimates from Poisson fixed-effects models using treatment intensity set to zero during the period of peak COVID-19 disruptions from Q1-Q3/2021 (1, main specification); treatment intensity excluded as missing values during the COVID-19 period (2); unadjusted treatment intensity with a COVID-19 dummy variable set to 1 from Q1-Q3/2021 (3); and unadjusted treatment intensity with a separate COVID-19 interaction term from Q1-Q3/2021.

## A.7: National interrupted time-series analysis

**Specification of national interrupted time-series models, rationale for linear and quadratic trend terms, seasonality controls, and comparison of delta-method and simulation-based uncertainty estimation.**

To triangulate subnational estimates, we fitted a complementary national interrupted time-series model using quarterly national TB notifications. The model employed Poisson regression with a population offset and included linear and quadratic secular trend terms, calendar-quarter indicators to capture seasonality, and a COVID-19 indicator.

National screening intensity, defined as aggregated screenings per 100,000 population, was included as the primary exposure of interest. Model-based predictions were used to estimate the difference in expected notifications under observed versus counterfactual intensity levels during the implementation period.

## A.8: Micro-costing methods

**Cost categories, allocation rules, assumptions underlying marginal cost per notification, and sensitivity analyses.**

The micro-costing analysis included only project costs covered by investments from the Stop TB Partnership, USAID and the Global Fund and excluded general health system and patient out-of-pocket costs. Project costs were estimated from a third-party sponsor perspective using detailed accounting records. Costs were categorized into personnel, activity-related costs (including consumables and incentives), capital and start-up costs, monitoring, and administration. Shared costs were allocated proportionally to intervention activities.

Marginal cost per additional TB notification was calculated by dividing total program costs by the estimated number of additional notifications attributable to the intervention over 112 treated province-quarters. National-level costs per additional notification were calculated using model-based estimates from the national interrupted time-series analysis.

All costs were converted from Vietnamese Dong (VNĐ) to US dollars (US$) using the average exchange rate during the implementation period (US$ 1 = VNĐ 23,716). Sensitivity analyses explored alternative denominators and uncertainty bounds.

**Table E: Chart of cost categorization by major type and subtypes.**

| **Type** | **Sub-type 1** | **Sub-type 2** |
| --- | --- | --- |
| HR | Director-Expat & Director | Director-Expat & Director |
| HR | Manager | Manager |
| HR | Officer | Officer |
| Activity | Screening | CXR fee |
| Activity | Screening | Counselling fee |
| Activity | Screening | Incentives for health staff |
| Activity | Screening | Incentives for agency staff |
| Activity | Screening | Travel support for patient |
| Activity | Testing | Other testing |
| Activity | Testing | Consumables |
| Activity | Testing | Support for lab technician |
| Activity | Testing | Specimen logistics stipends for agency staff |
| Activity | Testing | Specimen logistics stipends for health staff |
| Activity | Testing | Procurement of medical equipment |
| Activity | Case detect & report | Case notification & referral incentives for health staff |
| Activity | Case detect & report | Case treatment report incentives for health staff |
| Activity | Case detect & report | Data verification incentives for agency staff |
| Activity | Treatment follow-up & patient support | Treatment follow-up for health staff |
| Activity | Treatment follow-up & patient support | Incentives for agency staff (follow-up and linkage) |
| Activity | Treatment follow-up & patient support | Patient support |
| Activity | Treatment follow-up & patient support | Follow-up testing |
| Supervision | Hospital engagement | Hospital engagement |
| Supervision | Travel | Travel |
| One-time & Administrative | Training and Meeting | Training and Meeting |
| Procurement & CapEx | IT/Communications | Molecular testing consumables |
| Procurement & CapEx | IT/Communications | IT/Communications |
| One-time & Administrative | Others/Administrative | Others/Administrative |

CapEx, Capital Expenditure; Admin, Administrative.

## A.10: Provincial baseline characteristics for intervention, control and excluded provinces.

**Table F. Provincial demographics, TB burden, TB notifications and PPM notifications at baseline.**

|  |  | **Demographics** | | | **TB burden - Prevalence survey** | | | | | | **TB notification - VITIMES** | | **TB notification - PPM** | |
| --- | --- | --- | --- | --- | --- | --- | --- | --- | --- | --- | --- | --- | --- | --- |
| **Province** | **Region** | **Population** | **Area** | **Density** | **Prevalence rate** | **Prevalence** | **Unreached persons with TB** | **Estimated incidence rate** | **Estimated incidence** | **Estimated treatment coverage /saturation** | **2019** | **TB notification  rate, 2019** | **2019** | **Estimated PPM contribution** |
| Hà Nội | Red river delta | 8,866,078 | 3,359 | 2,640 | 262 | 23,229 | 19,107 | 147 | 13,033 | 32% | 4,242 | 48 | 1,383 | 33% |
| Hải Phòng | Red river delta | 2,150,337 | 1,523 | 1,412 | 262 | 5,634 | 4,050 | 147 | 3,161 | 50% | 1,835 | 85 | 90 | 5% |
| Hà Giang | North East | 922,013 | 7,930 | 116 | 262 | 2,416 | 1,817 | 147 | 1,355 | 44% | 568 | 62 | 119 | 21% |
| Thái Nguyên | North East | 1,377,930 | 3,536 | 390 | 262 | 3,610 | 2,954 | 147 | 2,026 | 32% | 911 | 66 | 53 | 6% |
| Thanh Hóa | North Central | 3,787,638 | 11,115 | 341 | 316 | 11,969 | 9,992 | 165 | 6,250 | 32% | 2,812 | 74 | 283 | 10% |
| Thừa Thiên Huế | North Central | 1,187,850 | 5,048 | 235 | 316 | 3,754 | 2,534 | 165 | 1,960 | 62% | 1,510 | 127 | 26 | 2% |
| Đà Nẵng | South Central | 1,292,642 | 1,285 | 1,006 | 316 | 4,085 | 2,324 | 165 | 2,133 | 83% | 1,480 | 114 | 0 | 0% |
| Quảng Nam | South Central | 1,564,036 | 10,575 | 148 | 316 | 4,942 | 3,478 | 165 | 2,581 | 57% | 1,566 | 100 | 182 | 12% |
| Bình Định | South Central | 1,533,134 | 6,066 | 253 | 316 | 4,845 | 3,691 | 165 | 2,530 | 46% | 1,420 | 93 | 204 | 14% |
| Bà Rịa - Vũng Tàu | South East | 1,214,615 | 1,981 | 613 | 380 | 4,616 | 3,294 | 212 | 2,575 | 51% | 1,307 | 108 | 240 | 18% |
| Bình Dương | South East | 2,918,695 | 2,695 | 1,083 | 380 | 11,091 | 8,153 | 212 | 6,188 | 47% | 2,941 | 101 | 0 | 0% |
| TP. Hồ Chí Minh | South East | 9,703,498 | 2,061 | 4,708 | 380 | 36,873 | 17,518 | 212 | 20,571 | 94% | 17,431 | 180 | 1,227 | 7% |
| Long An | Mekong river delta | 1,773,327 | 4,490 | 395 | 380 | 6,739 | 4,364 | 212 | 3,759 | 63% | 2,270 | 128 | 29 | 1% |
| Cần Thơ | Mekong river delta | 1,277,603 | 1,439 | 888 | 380 | 4,855 | 2,333 | 212 | 2,709 | 93% | 2,309 | 181 | 65 | 3% |
| Vĩnh Long | Mekong river delta | 1,039,243 | 1,475 | 705 | 380 | 3,949 | 2,305 | 212 | 2,203 | 75% | 1,494 | 144 | 259 | 17% |
| Đồng Nai | South East | 3,417,130 | 5,906 | 579 | 380 | 12,985 | 9,326 | 212 | 7,244 | 51% | 3,169 | 93 | 0 | 0% |
| Hải Dương | Red river delta | 2,002,258 | 1,668 | 1,200 | 262 | 5,246 | 4,127 | 147 | 2,943 | 38% | 1,361 | 68 | 53 | 4% |
| Đắk Lắk | Highland | 1,963,924 | 13,031 | 151 | 316 | 6,206 | 5,200 | 165 | 3,240 | 31% | 965 | 49 | 0 | 0% |
| Bắc Giang | North East | 1,996,534 | 3,851 | 518 | 262 | 5,231 | 3,556 | 147 | 2,935 | 57% | 1,979 | 99 | 104 | 5% |
| Nam Định | Red river delta | 1,912,591 | 1,668 | 1,147 | 262 | 5,011 | 3,707 | 147 | 2,812 | 46% | 1,730 | 90 | 175 | 10% |
| Gia Lai | Highland | 1,650,656 | 15,511 | 106 | 316 | 5,216 | 4,515 | 165 | 2,724 | 26% | 671 | 41 | 0 | 0% |
| Phú Thọ | North East | 1,557,871 | 3,535 | 441 | 262 | 4,082 | 3,510 | 147 | 2,290 | 25% | 701 | 45 | 132 | 19% |
| Bắc Ninh | Red river delta | 1,573,341 | 823 | 1,912 | 262 | 4,122 | 3,399 | 147 | 2,313 | 31% | 725 | 46 | 7 | 1% |
| Quảng Ninh | North East | 1,412,306 | 6,178 | 229 | 262 | 3,700 | 2,603 | 147 | 2,076 | 53% | 1,315 | 93 | 152 | 12% |
| Lâm Đồng | Highland | 1,378,230 | 9,783 | 141 | 316 | 4,355 | 3,785 | 165 | 2,274 | 25% | 553 | 40 | 10 | 2% |
| Hà Tĩnh | North Central | 1,335,968 | 5,991 | 223 | 316 | 4,222 | 3,504 | 165 | 2,204 | 33% | 890 | 67 | 106 | 12% |
| Bến Tre | Mekong river delta | 1,308,747 | 2,395 | 547 | 380 | 4,973 | 3,664 | 212 | 2,775 | 47% | 1,304 | 100 | 25 | 2% |
| Hưng Yên | Red river delta | 1,334,147 | 930 | 1,434 | 262 | 3,495 | 2,747 | 147 | 1,961 | 38% | 842 | 63 | 117 | 14% |
| Sơn La | North West | 1,347,289 | 14,124 | 95 | 262 | 3,530 | 3,207 | 147 | 1,981 | 16% | 387 | 29 | 0 | 0% |
| Quảng Ngãi | South Central | 1,269,648 | 5,135 | 247 | 316 | 4,012 | 3,607 | 165 | 2,095 | 19% | 1,128 | 89 | 0 | 0% |
| Bình Thuận | South East | 1,280,818 | 7,813 | 164 | 316 | 4,047 | 2,228 | 165 | 2,113 | 86% | 1,820 | 142 | 143 | 8% |
| Cà Mau | Mekong river delta | 1,214,783 | 5,295 | 229 | 380 | 4,616 | 3,113 | 212 | 2,575 | 58% | 1,417 | 117 | 181 | 13% |
| Tây Ninh | South East | 1,214,530 | 4,041 | 301 | 380 | 4,615 | 1,990 | 212 | 2,575 | 102% | 2,207 | 182 | 0 | 0% |
| Vĩnh Phúc | Red river delta | 1,236,388 | 1,235 | 1,001 | 262 | 3,239 | 3,141 | 147 | 1,817 | 5% | 996 | 81 | 0 | 0% |
| Bình Phước | South East | 1,078,629 | 6,877 | 157 | 380 | 4,099 | 3,260 | 212 | 2,287 | 37% | 899 | 83 | 12 | 1% |
| Ninh Bình | Red river delta | 1,040,303 | 1,387 | 750 | 262 | 2,726 | 2,029 | 147 | 1,529 | 46% | 802 | 77 | 194 | 24% |
| Bạc Liêu | Mekong river delta | 930,832 | 2,669 | 349 | 380 | 3,537 | 2,384 | 212 | 1,973 | 58% | 1,085 | 117 | 50 | 5% |
| Quảng Bình | North Central | 931,317 | 8,065 | 115 | 316 | 2,943 | 2,136 | 165 | 1,537 | 53% | 825 | 89 | 0 | 0% |
| Phú Yên | South Central | 893,261 | 5,023 | 178 | 316 | 2,823 | 2,282 | 165 | 1,474 | 37% | 670 | 75 | 0 | 0% |
| Hòa Bình | North West | 902,595 | 4,591 | 197 | 262 | 2,365 | 2,365 | 147 | 1,327 | 0% | 445 | 49 | 81 | 18% |
| Hà Nam | Red river delta | 902,882 | 861 | 1,049 | 262 | 2,366 | 1,624 | 147 | 1,327 | 56% | 770 | 85 | 103 | 13% |
| Yên Bái | North East | 875,944 | 6,888 | 127 | 262 | 2,295 | 2,082 | 147 | 1,288 | 17% | 274 | 31 | 29 | 11% |
| Tuyên Quang | North East | 829,852 | 5,868 | 141 | 262 | 2,174 | 1,884 | 147 | 1,220 | 24% | 322 | 39 | 23 | 7% |
| Lạng Sơn | North East | 819,265 | 8,310 | 99 | 262 | 2,146 | 1,354 | 147 | 1,204 | 66% | 955 | 117 | 205 | 21% |
| Lào Cai | North East | 802,780 | 6,364 | 126 | 262 | 2,103 | 1,834 | 147 | 1,180 | 23% | 319 | 40 | 0 | 0% |
| Quảng Trị | North Central | 663,845 | 4,740 | 140 | 316 | 2,098 | 1,262 | 165 | 1,095 | 76% | 745 | 112 | 0 | 0% |
| Đắk Nông | Highland | 703,732 | 6,509 | 108 | 316 | 2,224 | 1,914 | 165 | 1,161 | 27% | 316 | 45 | 0 | 0% |
| Điện Biên | North West | 666,351 | 9,541 | 70 | 262 | 1,746 | 1,586 | 147 | 980 | 16% | 177 | 27 | 8 | 5% |
| Ninh Thuận | South East | 617,609 | 3,355 | 184 | 316 | 1,952 | 1,175 | 165 | 1,019 | 76% | 740 | 120 | 82 | 11% |
| Kon Tum | Highland | 606,456 | 9,674 | 63 | 316 | 1,916 | 1,485 | 165 | 1,001 | 43% | 341 | 56 | 175 | 51% |
| Cao Bằng | North East | 562,531 | 6,700 | 84 | 262 | 1,474 | 1,217 | 147 | 827 | 31% | 300 | 53 | 91 | 30% |
| Lai Châu | North West | 502,873 | 9,069 | 55 | 262 | 1,318 | 1,108 | 147 | 739 | 28% | 145 | 29 | 0 | 0% |
| Bắc Kạn | North East | 332,371 | 4,860 | 68 | 262 | 871 | 750 | 147 | 489 | 25% | 115 | 35 | 0 | 0% |
| Nghệ An | North Central | 3,498,331 | 16,494 | 212 | 316 | 11,055 | 9,355 | 165 | 5,772 | 29% | 2,216 | 63 | 568 | 26% |
| An Giang | Mekong river delta | 1,922,319 | 3,537 | 544 | 380 | 7,305 | 2,060 | 212 | 4,075 | 129% | 4,590 | 239 | 999 | 22% |
| Thái Bình | Red river delta | 1,913,415 | 1,571 | 1,218 | 262 | 5,013 | 4,008 | 147 | 2,813 | 36% | 1,349 | 71 | 269 | 20% |
| Tiền Giang | Mekong river delta | 1,803,864 | 2,511 | 719 | 380 | 6,855 | 4,745 | 212 | 3,824 | 55% | 2,082 | 115 | 81 | 4% |
| Kiên Giang | Mekong river delta | 1,775,677 | 6,349 | 280 | 380 | 6,748 | 3,724 | 212 | 3,764 | 80% | 2,709 | 153 | 494 | 18% |
| Đồng Tháp | Mekong river delta | 1,608,021 | 3,384 | 475 | 380 | 6,110 | 3,431 | 212 | 3,409 | 79% | 2,757 | 171 | 535 | 19% |
| Khánh Hòa | South Central | 1,279,470 | 5,138 | 249 | 316 | 4,043 | 2,380 | 165 | 2,111 | 79% | 1,526 | 119 | 263 | 17% |
| Sóc Trăng | Mekong river delta | 1,210,895 | 3,312 | 366 | 380 | 4,601 | 2,363 | 212 | 2,567 | 87% | 2,096 | 173 | 1,567 | 75% |
| Trà Vinh | Mekong river delta | 1,025,818 | 2,358 | 435 | 380 | 3,898 | 2,710 | 212 | 2,175 | 55% | 1,214 | 118 | 33 | 3% |
| Hậu Giang | Mekong river delta | 733,389 | 1,622 | 452 | 380 | 2,787 | 1,345 | 212 | 1,555 | 93% | 1,316 | 179 | 34 | 3% |

Shaded cells denote intervention (green), control (blue) and excluded (grey) provinces.

# Section B Supplementary Results

## B.1: Descriptive statistics of healthcare providers and verbally assessed individuals.

**Table G. Healthcare provider characteristics.**

|  | **Pharmacies** | | **Private practices** | | **Clinics** | | **Hospitals** | | **Other** | | **Total** | |
| --- | --- | --- | --- | --- | --- | --- | --- | --- | --- | --- | --- | --- |
|  | **n** | **%^a^** | **n** | **%^a^** | **n** | **%^a^** | **n** | **%^a^** | **n** | **%^a^** | **n^b^** | **%^a^** |
| **Total** | **1,890** | **59.9** | **526** | **16.7** | **309** | **9.80** | **195** | **6.2** | **234** | **7.4** | **3,154** | **100.0** |
| **HEALTHCARE SERVICES** |  |  |  |  |  |  |  |  |  |  |  |  |
| Typology |  |  |  |  |  |  |  |  |  |  |  |  |
| Solo-practice | 0 | 0.0 | 526 | 100.0 | 0 | 0.0 | 0 | 0.0 | 0 | 0.0 | 526 | 16.7 |
| Multi-doctor clinic | 0 | 0.0 | 0 | 0.0 | 271 | 87.7 | 0 | 0.0 | 0 | 0.0 | 271 | 8.6 |
| Pharmacy | 1,890 | 100.0 | 0 | 0.0 | 0 | 0.0 | 0 | 0.0 | 0 | 0.0 | 1,890 | 59.9 |
| Private Hospital | 0 | 0.0 | 0 | 0.0 | 0 | 0.0 | 65 | 33.3 | 0 | 0.0 | 65 | 2.1 |
| Public Hospital | 0 | 0.0 | 0 | 0.0 | 0 | 0.0 | 130 | 66.7 | 0 | 0.0 | 130 | 4.1 |
| X-ray site/laboratory | 0 | 0.0 | 0 | 0.0 | 0 | 0.0 | 0 | 0.0 | 50 | 21.4 | 50 | 1.6 |
| Workplace healthcare provider | 0 | 0.0 | 0 | 0.0 | 0 | 0.0 | 0 | 0.0 | 21 | 9.0 | 21 | 0.7 |
| Private polyclinic | 0 | 0.0 | 0 | 0.0 | 38 | 12.3 | 0 | 0.0 | 0 | 0.0 | 38 | 1.2 |
| Traditional healers | 0 | 0.0 | 0 | 0.0 | 0 | 0.0 | 0 | 0.0 | 15 | 6.4 | 15 | 0.5 |
| Other | 0 | 0.0 | 0 | 0.0 | 0 | 0.0 | 0 | 0.0 | 148 | 63.3 | 148 | 4.7 |
| CXR on-site |  |  |  |  |  |  |  |  |  |  |  |  |
| No | 1,886 | 99.8 | 503 | 95.6 | 168 | 54.4 | 42 | 21.5 | 188 | 80.3 | 2,787 | 88.4 |
| Yes | 4 | 0.2 | 23 | 4.4 | 141 | 45.6 | 153 | 78.5 | 46 | 19.7 | 367 | 11.6 |
| Xpert on-site |  |  |  |  |  |  |  |  |  |  |  |  |
| No | 1,890 | 100.0 | 526 | 100.0 | 301 | 97.4 | 169 | 86.7 | 224 | 95.7 | 3,110 | 98.6 |
| Yes | 0 | 0.0 | 0 | 0.0 | 8 | 2.6 | 26 | 13.3 | 10 | 4.3 | 44 | 1.4 |
| TB services |  |  |  |  |  |  |  |  |  |  |  |  |
| No§ | 1,889 | 100.0 | 508 | 96.6 | 304 | 98.4 | 164 | 84.1 | 224 | 95.7 | 3,089 | 97.9 |
| Yes | 1 | 0.1 | 18 | 3.4 | 5 | 1.6 | 31 | 15.9 | 10 | 4.3 | 65 | 2.1 |
| **SERVICE COVERAGE** |  |  |  |  |  |  |  |  |  |  |  |  |
| Region |  |  |  |  |  |  |  |  |  |  |  |  |
| North | 950 | 50.3 | 162 | 30.8 | 75 | 24.3 | 73 | 37.4 | 174 | 74.4 | 1,434 | 45.5 |
| Red River Delta | 950 | 50.3 | 159 | 30.2 | 75 | 24.3 | 60 | 30.8 | 174 | 74.4 | 1,418 | 45.0 |
| Northeast | 0 | 0.0 | 3 | 0.6 | 0 | 0.0 | 13 | 6.7 | 0 | 0.0 | 16 | 0.5 |
| Central | 95 | 5.0 | 38 | 7.2 | 84 | 27.2 | 55 | 28.2 | 26 | 11.1 | 298 | 9.5 |
| North Central Coast | 11 | 0.6 | 11 | 2.1 | 20 | 6.5 | 30 | 15.4 | 7 | 3.0 | 79 | 2.5 |
| South Central Coast | 84 | 4.4 | 27 | 5.1 | 64 | 20.7 | 25 | 12.8 | 19 | 8.1 | 219 | 6.9 |
| Central Highlands | 845 | 44.7 | 326 | 62.0 | 150 | 48.5 | 67 | 34.4 | 34 | 14.5 | 1,422 | 45.1 |
| South | 812 | 43.0 | 314 | 59.7 | 142 | 46.0 | 60 | 30.8 | 31 | 13.3 | 1,359 | 43.1 |
| Southeast | 33 | 1.8 | 12 | 2.3 | 8 | 2.6 | 7 | 3.6 | 3 | 1.3 | 63 | 2.0 |
| Mekong River Delta | 950 | 50.3 | 162 | 30.8 | 75 | 24.3 | 73 | 37.4 | 174 | 74.4 | 1,434 | 45.5 |
| Urbanicity |  |  |  |  |  |  |  |  |  |  |  |  |
| Rural | 247 | 13.1 | 83 | 15.8 | 63 | 20.4 | 44 | 22.6 | 55 | 23.5 | 492 | 15.6 |
| Urban | 1,643 | 86.9 | 443 | 84.2 | 246 | 79.6 | 151 | 77.4 | 179 | 76.5 | 2,662 | 84.4 |

^a^ Percentages in the first row are the proportion of total; all other rows are the proportion of the subgroup; § For covariates with N<1,719,382, the actual N size is stated next to the covariate name in the first column. Bac(+), bacteriologically confirmed; CXR, chest x-ray.

**Table H. Characteristics of verbally assessed individuals.**

|  | **Pharmacies** | | **Private practices** | | **Clinics** | | **Hospitals** | | **Other** | | **Total** | |
| --- | --- | --- | --- | --- | --- | --- | --- | --- | --- | --- | --- | --- |
|  | **n** | **%^a^** | **n** | **%^a^** | **n** | **%^a^** | **n** | **%^a^** | **n** | **%^a^** | **n^b^** | **%^a^** |
| **Total** | **4,442** | **0.3** | **61,978** | **3.6** | **283,681** | **16.50** | **1,356,781** | **78.9** | **12,500** | **0.7** | **1,719,382** | **100.0** |
| **DEMOGRAPHICS** |  |  |  |  |  |  |  |  |  |  |  |  |
| Sex |  |  |  |  |  |  |  |  |  |  |  |  |
| Male | 2,283 | 51.4 | 30,964 | 50.0 | 143,452 | 50.6 | 692,534 | 51.0 | 6,474 | 51.8 | 875,707 | 50.9 |
| Female | 2,159 | 48.6 | 31,014 | 50.0 | 140,229 | 49.4 | 664,247 | 49.0 | 6,026 | 48.2 | 843,675 | 49.1 |
| Age (N=1,611,234)^b^ |  |  |  |  |  |  |  |  |  |  |  |  |
| <=15 years | 209 | 4.8 | 3,032 | 5.0 | 17,267 | 6.5 | 131,762 | 10.4 | 290 | 3.4 | 152,560 | 9.5 |
| 16-35 years | 887 | 20.3 | 13,883 | 22.8 | 63,043 | 23.8 | 248,050 | 19.5 | 2,739 | 32.4 | 328,602 | 20.4 |
| 36-55 years | 1,691 | 38.6 | 20,262 | 33.2 | 90,541 | 34.1 | 375,003 | 29.5 | 3,288 | 38.9 | 490,785 | 30.5 |
| >55 years | 1,589 | 36.3 | 23,805 | 39.0 | 94,488 | 35.6 | 517,266 | 40.7 | 2,139 | 25.3 | 639,287 | 39.7 |
| *Median age (IQR)* | *36* | *(16-56)* | *36* | *(16-56)* | *36* | *(16-56)* | *36* | *(16-56)* | *36* | *(16-56)* | *36* | *(16-56)* |
| Region |  |  |  |  |  |  |  |  |  |  |  |  |
| North | 392 | 8.8 | 13,923 | 22.5 | 80,822 | 28.5 | 420,516 | 31.0 | 829 | 6.6 | 516,482 | 30.0 |
| Red River Delta | 392 | 8.8 | 13,869 | 22.4 | 80,822 | 28.5 | 399,840 | 29.5 | 829 | 6.6 | 495,752 | 28.8 |
| Northeast | 0 | 0.0 | 54 | 0.1 | 0 | 0.0 | 20,676 | 1.5 | 0 | 0.0 | 20,730 | 1.2 |
| Central | 1,159 | 26.1 | 10,808 | 17.4 | 35,918 | 12.7 | 285,397 | 21.0 | 700 | 5.6 | 333,982 | 19.4 |
| North Central Coast | 17 | 0.4 | 132 | 0.2 | 33,483 | 11.8 | 152,707 | 11.3 | 0 | 0.0 | 186,339 | 10.8 |
| South Central Coast | 1,142 | 25.7 | 10,676 | 17.2 | 2,435 | 0.9 | 132,690 | 9.8 | 700 | 5.6 | 147,643 | 8.6 |
| Central Highlands | 2,724 | 61.3 | 35,577 | 57.4 | 148,056 | 52.2 | 581,277 | 42.8 | 10,971 | 87.8 | 778,605 | 45.3 |
| South | 2,891 | 65.1 | 37,247 | 60.1 | 166,941 | 58.9 | 650,868 | 48.0 | 10,971 | 87.8 | 868,918 | 50.5 |
| Southeast | 167 | 3.8 | 1,670 | 2.7 | 18,885 | 6.7 | 69,591 | 5.1 | 0 | 0.0 | 90,313 | 5.3 |
| Mekong River Delta | 4,442 | 0.3 | 61,978 | 3.6 | 283,681 | 16.5 | 1,356,781 | 78.9 | 12,500 | 0.7 | 1,719,382 | 100.0 |
| Urbanicity (N=1,719,381) |  |  |  |  |  |  |  |  |  |  |  |  |
| Rural | 637 | 14.3 | 13,268 | 21.4 | 46,449 | 16.4 | 118,024 | 8.7 | 202 | 1.6 | 178,580 | 10.4 |
| Urban | 3,805 | 85.7 | 48,710 | 78.6 | 237,232 | 83.6 | 1,238,756 | 91.3 | 12,298 | 98.4 | 1,540,801 | 89.6 |
| **CLINICAL CHARACTERISTICS** |  |  |  |  |  |  |  |  |  |  |  |  |
| Number of W4SS symptoms on W4SS |  |  |  |  |  |  |  |  |  |  |  |  |
| Asymptomatic | 669 | 15.1 | 6,291 | 10.2 | 88,682 | 31.3 | 361,721 | 26.7 | 7,182 | 57.5 | 464,545 | 27.0 |
| 1 symptom | 2,899 | 65.3 | 43,365 | 70.0 | 146,834 | 51.8 | 691,346 | 51.0 | 4,095 | 32.8 | 888,539 | 51.7 |
| 2 symptoms | 794 | 17.9 | 10,129 | 16.3 | 42,971 | 15.2 | 256,370 | 18.9 | 770 | 6.2 | 311,034 | 18.1 |
| 3 symptoms | 74 | 1.7 | 1,926 | 3.1 | 4,836 | 1.7 | 42,692 | 3.2 | 451 | 3.6 | 49,979 | 2.9 |
| 4 symptoms | 6 | 0.1 | 267 | 0.4 | 358 | 0.1 | 4,652 | 0.3 | 2 | 0.0 | 5,285 | 0.3 |
| Cough (N=1,686,966) |  |  |  |  |  |  |  |  |  |  |  |  |
| No | 1,460 | 32.9 | 14,679 | 23.7 | 130,005 | 45.8 | 500,738 | 37.8 | 7,402 | 59.2 | 654,284 | 38.8 |
| Yes | 2,982 | 67.1 | 47,299 | 76.3 | 153,676 | 54.2 | 823,627 | 62.2 | 5,098 | 40.8 | 1,032,682 | 61.2 |
| Fever (N=1,686,966) |  |  |  |  |  |  |  |  |  |  |  |  |
| No | 3,615 | 81.4 | 51,032 | 82.3 | 236,271 | 83.3 | 1,042,673 | 78.7 | 11,657 | 93.3 | 1,345,248 | 79.7 |
| Yes | 827 | 18.6 | 10,946 | 17.7 | 47,410 | 16.7 | 281,686 | 21.3 | 843 | 6.7 | 341,712 | 20.3 |
| Night sweats (N=1,686,966) |  |  |  |  |  |  |  |  |  |  |  |  |
| No | 4,171 | 93.9 | 56,384 | 91.0 | 258,411 | 91.1 | 1,214,491 | 91.7 | 12,329 | 98.6 | 1,545,786 | 91.6 |
| Yes | 271 | 6.1 | 5,594 | 9.0 | 25,270 | 8.9 | 109,873 | 8.3 | 171 | 1.4 | 141,179 | 8.4 |
| Weight loss (N=1,686,966) |  |  |  |  |  |  |  |  |  |  |  |  |
| No | 3,789 | 85.3 | 55,348 | 89.3 | 261,321 | 92.1 | 1,188,781 | 89.8 | 11,616 | 92.9 | 1,520,855 | 90.2 |
| Yes | 653 | 14.7 | 6,630 | 10.7 | 22,360 | 7.9 | 135,584 | 10.2 | 884 | 7.1 | 166,111 | 9.9 |
| History of TB (N=1,694,444) |  |  |  |  |  |  |  |  |  |  |  |  |
| No | 4,143 | 93.3 | 61,366 | 99.0 | 281,583 | 99.3 | 1,323,269 | 99.4 | 12,457 | 99.7 | 1,682,818 | 99.3 |
| Yes | 299 | 6.7 | 612 | 1.0 | 2,098 | 0.7 | 8,574 | 0.6 | 43 | 0.3 | 11,626 | 0.7 |
| **SUB-CLINICAL CHARACTERISTICS** |  |  |  |  |  |  |  |  |  |  |  |  |
| CXR screen (N=1,688,471) |  |  |  |  |  |  |  |  |  |  |  |  |
| Normal | 2,481 | 59.6 | 46,223 | 75.9 | 251,835 | 90.7 | 1,198,442 | 89.9 | 11,151 | 90.7 | 1,510,132 | 89.4 |
| Abnormal – TB | 1,681 | 40.4 | 14,700 | 24.1 | 25,984 | 9.4 | 134,829 | 10.1 | 1,145 | 9.3 | 178,339 | 10.6 |
| Bacteriologic testing |  |  |  |  |  |  |  |  |  |  |  |  |
| Tested | 1,836 | 41.4 | 13,886 | 22.4 | 19,704 | 7.0 | 124,263 | 9.2 | 1,179 | 9.4 | 160,868 | 9.4 |
| No sputum test | 2,606 | 58.7 | 48,092 | 77.6 | 263,977 | 93.1 | 1,232,518 | 90.8 | 11,321 | 90.6 | 1,558,514 | 90.6 |
| Diagnosis (N=1,718,608) |  |  |  |  |  |  |  |  |  |  |  |  |
| Bac(+) | 478 | 10.8 | 3,312 | 5.4 | 3,935 | 1.4 | 19,076 | 1.4 | 161 | 1.3 | 26,962 | 1.6 |
| Clinical diagnosis | 47 | 1.1 | 1,278 | 2.1 | 455 | 0.2 | 2,797 | 0.2 | 16 | 0.1 | 4,593 | 0.3 |
| No TB | 3,917 | 88.2 | 57,352 | 92.6 | 279,109 | 98.5 | 1,334,352 | 98.4 | 12,323 | 98.6 | 1,687,053 | 98.2 |
| **TREATMENT CHARACTERISTICS** |  |  |  |  |  |  |  |  |  |  |  |  |
| Linkage to care (N=31,548) |  |  |  |  |  |  |  |  |  |  |  |  |
| Bacteriologically-confirmed | 430 | 82 | 3,196 | 70 | 3,591 | 82 | 16,569 | 76 | 140 | 79 | 23,926 | 76 |
| Drug-susceptible TB | 403 | 76.8 | 3,029 | 66.0 | 3,454 | 78.7 | 16,135 | 73.8 | 134 | 75.7 | 23,155 | 73.4 |
| Drug-resistant TB | 27 | 5.1 | 167 | 3.6 | 137 | 3.1 | 434 | 2.0 | 6 | 3.4 | 771 | 2.4 |
| Clinical diagnosis | 47 | 9.0 | 1,276 | 27.8 | 455 | 10.4 | 2,782 | 12.7 | 16 | 9.0 | 4,576 | 14.5 |
| Pre-treatment LTFU | 48 | 9.1 | 118 | 2.5 | 344 | 7.9 | 2,515 | 11.6 | 21 | 11.9 | 3,046 | 9.8 |
| Treatment (N=31,551) |  |  |  |  |  |  |  |  |  |  |  |  |
| NTP | 472 | 89.9 | 1,294 | 28.2 | 3,174 | 72.3 | 17,651 | 80.7 | 96 | 54.2 | 22,687 | 71.9 |
| Private provider | 5 | 1.0 | 3,177 | 69.2 | 872 | 19.9 | 1,706 | 7.8 | 60 | 33.9 | 5,820 | 18.5 |
| Pre-treatment LTFU | 48 | 9.1 | 118 | 2.6 | 344 | 7.8 | 2,513 | 11.5 | 21 | 11.9 | 3,044 | 9.7 |

^a^ Percentages in the first row are the proportion of total; all other rows are the proportion of the subgroup; § For covariates with N<1,719,382, the actual N size is stated next to the covariate name in the first column. Bac(+), bacteriologically confirmed; CXR, chest x-ray; LTFU, loss to follow-up.

## B.2: Provider engagement cascade.

**Fig D: Flow of healthcare providers through engagement, recruitment, and retention.**

The flowchart shows the progression of healthcare providers from provincial master registries through eligibility assessment, engagement and recruitment, to the number retained at the end of the study. Boxes indicate the number of providers at each stage, with reasons for exclusion shown alongside.

**Table I: Provider engagement cascade by provider type.**

|  | **Pharmacies** | | **Private practices** | | **Clinics** | | **Hospitals** | | **Other** | | **Total** | |
| --- | --- | --- | --- | --- | --- | --- | --- | --- | --- | --- | --- | --- |
|  | **n** | **%^a^** | **n** | **%^a^** | **n** | **%^a^** | **n** | **%^a^** | **n** | **%^a^** | **n** | **%^a^** |
| Providers enumerated | 10,508 | 53.8 | 2,293 | 11.7 | 5,716 | 29.3 | 364 | 1.9 | 639 | 3.3 | 19,520 | 100.0 |
| Providers deemed eligible | 9,830 | 93.5 | 2,047 | 89.3 | 5,103 | 89.3 | 358 | 98.4 | 585 | 91.5 | 17,923 | 91.8 |
| Providers engaged **≥**1 times | 5,446 | 55.4 | 1,643 | 80.3 | 902 | 17.7 | 300 | 83.8 | 296 | 50.6 | 8,587 | 47.9 |
| Providers with a signed contract | 1,888 | 35.1 | 525 | 32.3 | 442 | 50.3 | 194 | 66.0 | 105 | 35.7 | 3,154 | 36.7 |
| **Providers with** **≥1 CXR completed** | **160** | **8.5** | **187** | **35.6** | **260** | **58.8** | **185** | **95.4** | **27** | **25.7** | **819** | **26.0** |
| *CXR completed per signed provider* | *2* | | *116* | | *629* | | *6,873* | | *119* | | *535* | |
| **Providers with** **≥1 TB detections** | **44** | **27.5** | **127** | **67.9** | **173** | **66.5** | **170** | **91.9** | **18** | **66.7** | **532** | **65.0** |
| *TB detected per signed provider (NNS)* | *0.3 (8)* | | *8.7 (13)* | | *9.9 (63)* | | *113.1 (61)* | | *1.6 (71)* | | *10.0 (53)* | |

^a^ Percentages are calculated relative to the total (first row) or the preceding row (subsequent rows).

CXR=chest X-ray; NNS=number needed to screen.

## B.3: Tuberculosis care cascade by strategy.

**Table J: Tuberculosis care cascade by provider type, Strategy 1 – diagnosis and referral to the National Tuberculosis Control Programme**

|  | **Pharmacies** | | **Private practices** | | **Clinics** | | **Hospitals** | | **Other** | | **Total** | |
| --- | --- | --- | --- | --- | --- | --- | --- | --- | --- | --- | --- | --- |
|  | **n** | **%^a^** | **n** | **%^a^** | **n** | **%^a^** | **n** | **%^a^** | **n** | **%^a^** | **n** | **%^a^** |
| Verbally screened | 4,442 | 0.3 | 61,978 | 3.6 | 283,681 | 16.5 | 1,356,814 | 78.9 | 12,467 | 0.7 | 1,719,382 | -- |
| Screened by CXR | 4,162 | 93.7 | 60,923 | 98.3 | 277,819 | 97.9 | 1,333,304 | 98.3 | 12,263 | 98.4 | 1,688,471 | 98.2 |
| CXR abnormal TB | 1,681 | 40.4 | 14,700 | 24.1 | 25,984 | 9.4 | 134,861 | 10.1 | 1,113 | 9.1 | 178,339 | 10.6 |
| Tested after abnormal CXR^b^ | 1,491 | 88.7 | 13,334 | 90.7 | 18,559 | 71.4 | 110,402 | 81.9 | 1,006 | 90.4 | 144,792 | 81.2 |
| Bac(+) TB – abnormal CXR | 427 | 28.6 | 3,243 | 24.3 | 3,820 | 20.6 | 18,167 | 16.5 | 151 | 15.0 | 25,808 | 17.8 |
| Rif-resistant TB – abnormal CXR | 29 | 6.8 | 187 | 5.8 | 199 | 5.2 | 570 | 3.1 | 6 | 4.0 | 991 | 3.8 |
| No CXR/CXR normal | 2,761 | 62.2 | 47,278 | 76.3 | 257,697 | 90.8 | 1,221,953 | 90.1 | 11,354 | 91.1 | 1,541,043 | 89.6 |
| Testing due to other risk factors | 345 | 12.5 | 552 | 1.2 | 1,145 | 0.4 | 13,893 | 1.1 | 141 | 1.2 | 16,076 | 1.0 |
| Bac(+) TB – other risk factors | 52 | 15.1 | 69 | 12.5 | 121 | 10.6 | 974 | 7.0 | 5 | 3.5 | 1,221 | 7.6 |
| Rif-resistant TB – other risk factors | 3 | 5.8 | 1 | 1.4 | 8 | 6.6 | 24 | 2.5 | 0 | 0.0 | 36 | 2.9 |
| Total Bac(+) TB | 479 | 10.8 | 3,312 | 5.3 | 3,941 | 1.4 | 19,141 | 1.4 | 156 | 1.3 | 27,029 | 1.6 |
| Clinically diagnosed TB | 47 | 1.1 | 1,278 | 2.1 | 455 | 0.2 | 2,797 | 0.2 | 16 | 0.1 | 4,593 | 0.3 |
| **Total all forms of TB** | **526** | **11.8** | **4,590** | **7.4** | **4,396** | **1.5** | **21,938** | **1.6** | **172** | **1.4** | **31,622** | **1.8** |
| All forms of TB started on treatment | 477 | 90.7 | 4,472 | 97.4 | 4,046 | 92.0 | 19,356 | 88.2 | 151 | 87.8 | 28,502 | 90.1 |
| NTP treatment | 472 | 99.0 | 1,294 | 28.9 | 3,174 | 78.4 | 17,646 | 91.2 | 92 | 60.9 | 22,678 | 79.6 |
| Private treatment | 5 | 1.0 | 3,178 | 71.1 | 872 | 21.6 | 1,710 | 8.8 | 59 | 39.1 | 5,824 | 20.4 |
| Within PPM Model 5 – Strategy 2 | 1 | 20.0 | 2,208 | 69.5 | 583 | 66.9 | 655 | 38.3 | 18 | 30.5 | 3,465 | 59.5 |
| Outside PPM Model 5 | 4 | 80.0 | 970 | 30.5 | 289 | 33.1 | 1,055 | 61.7 | 41 | 69.5 | 2,359 | 40.5 |

**^a^** Percentages are calculated using the nearest preceding indented row as the denominator (for example, the percentage of “CXR normal” is calculated using “Screened by CXR” as the denominator). ^b^ Tested using smear microscopy (AFB) or Xpert MTB/RIF, depending on availability at the treatment site

Bac(+)=bacteriologically confirmed; CXR=chest X-ray; NTP=National Tuberculosis Control Programme.

**Table K: Tuberculosis care cascade, Strategy 2 – private treatment reporting.**

|  | **Total** | |
| --- | --- | --- |
|  | **n** | **%** |
| Providers eligible to treat^a^ | 8,093 | -- |
| Providers reporting private treatment | 134 | 1.7 |
| **All forms of TB on private treatment** | **12,839** | **--** |
| Tested for Bac(+) confirmation & DST^b^ | 10,502 | 81.8 |
| Bac(+) TB detected | 7,379 | 70.3 |
| RIF Resistant TB detected | 128 | 1.7 |
| Bac(+) TB patients successfully treated | 5,770 | 78.2 |
| All forms TB patients successfully treated | 9,414 | 73.3 |
| **All forms of TB notified in VITIMES** | **12,231** | **95.3** |
| Successful treatment outcome in VITIMES | 9,414 | 73.3 |

^a^ All types of eligible providers, excluding pharmacies; ^b^ Rifampicin resistance assessed using Xpert MTB/RIF or Xpert MTB/RIF Ultra.

Bac(+)=bacteriologically confirmed; DST=drug-susceptibility testing; VITIMES=National Tuberculosis Control Programme routine electronic surveillance system.

## B.4: National interrupted time-series analysis model results.

**Table L: National interrupted time-series analysis of tuberculosis notifications, 2016-2025.**

|  | **IRR (95% CI)** | ***p*-value** |
| --- | --- | --- |
| **Intensity (per 100,000 population)** | **1.00035 [1.00018, 1.00052]** | **<0.001** |
| Linear time trend (t) | 0.98961 [0.98175, 0.99753] | 0.010 |
| Quadratic time trend (t²) | 1.00013 [0.99993, 1.00032] | 0.198 |
| Quarter (ref = Q1) |  |  |
| Q2 | 1.07035 [1.00121, 1.14425] | 0.046 |
| Q3 | 1.04279 [0.94168, 1.15476] | 0.421 |
| Q4 | 1.06269 [0.98538, 1.14607] | 0.115 |
| COVID-19 period indicator | 0.82293 [0.59014, 1.14755] | 0.251 |
| Constant | 0.00025 [0.00023, 0.00027] | <0.001 |

Estimates are from a Poisson regression with a population offset. Screening intensity is defined as the number of persons screened per 100,000 population per quarter. The model includes linear and quadratic secular time trends, quarterly seasonality indicators, and a COVID-19 disruption indicator. Robust standard errors were used.

**Fig E: National interrupted time-series analysis of tuberculosis notifications, Viet Nam, 2016–2025.**

Quarterly notifications of all forms of tuberculosis are shown in dark blue and the model-based counterfactual in orange. The model is a Poisson interrupted time-series regression with a population offset, linear and quadratic secular time trends, quarterly seasonality indicators, and a COVID-19 disruption indicator. During the intervention period the counterfactual trajectory is stratified by scenarios of actual (dashed) and zero screening intensity. Vertical dashed lines denote program phase boundaries.

Quarterly notifcations of all forms of TB

|  | National | Fitted | Counterfactual |
| --- | --- | --- | --- |
| 2016 1 | 18632 |  | 18886.4 |
| 2016 2 | 20046 |  | 20077.3 |
| 2016 3 | 19651 |  | 19432.1 |
| 2016 4 | 20346 |  | 19677.6 |
| 2017 1 | 17402 |  | 18401.9 |
| 2017 2 | 19505 |  | 19581.9 |
| 2017 3 | 19505 |  | 18971.4 |
| 2017 4 | 18336 |  | 19230.3 |
| 2018 1 | 16906 |  | 18019.7 |
| 2018 2 | 18761 |  | 19198.2 |
| 2018 3 | 19255 |  | 18622.1 |
| 2018 4 | 19104 |  | 18898.9 |
| 2019 1 | 17656 |  | 17709 |
| 2019 2 | 19525 |  | 18885.3 |
| 2019 3 | 20604 |  | 18336.2 |
| 2019 4 | 19025 | 18626.7 | 18626.7 |
| 2020 1 | 17802 | 17484.1 | 17468.6 |
| 2020 2 | 16804 | 18733.5 | 18646.4 |
| 2020 3 | 19417 | 18438.6 | 18121.2 |
| 2020 4 | 20349 | 18856.7 | 18425.5 |
| 2021 1 | 18692 | 14454.6 | 14184.7 |
| 2021 2 | 16974 | 15340.5 | 15144.8 |
| 2021 3 | 8952 | 14823 | 14721.9 |
| 2021 4 | 15030 | 18697.1 | 18194.5 |
| 2022 1 | 15321 | 17691.9 | 17126 |
| 2022 2 | 20387 | 19088.6 | 18311.6 |
| 2022 3 | 20086 | 18958.9 | 17826 |
| 2022 4 | 20045 | 19416.8 | 18156.1 |
| 2023 1 | 18364 | 18109.5 | 17031.9 |
| 2023 2 | 19252 | 20036 | 18219.2 |
| 2023 3 | 20001 | 20187.9 | 17744.1 |
| 2023 4 | 20376 | 20449.1 | 18081 |
| 2024 1 | 17128 | 17055.5 | 17055.5 |
| 2024 2 | 18120 |  | 18271.3 |
| 2024 3 | 18120 |  | 17820.9 |
| 2024 4 | 19428 |  | 18185.8 |
| 2025 1 | 16976 |  | 17066.4 |
| 2025 2 | 18124 |  | 18285.5 |

## B.5: Micro-costing results by primary and sub-categories.

**Fig F: Breakdown of total program costs by primary and sub-categories.**

The pie-in-pie chart shows the distribution of total program costs (US$ 5,877,868) by major cost category (inner ring) and corresponding sub-categories (outer ring). Inner segments represent procurement and capital expenditure, activity costs, human resources, supervision, and administrative and one-time meeting costs, while outer segments provide a detailed breakdown of expenditures within each category; values are shown as absolute costs and percentages of total program expenditure.

## B.6: Intermediate outcome analysis: PPM notifications

To examine whether the intervention increased notifications within the PPM channel itself, we repeated the primary analyses using PPM-reported TB notifications (PPM notifications) as the primary outcome. This outcome represents an intermediate step between provider-level screening and diagnostic yield and total national TB notifications, and is more closely aligned with the intervention mechanism.

In mirroring the primary analysis, we described mean quarterly PPM notifications in the areas implementing PPM Model 5 and traditional PPM activities, and estimated group-time average treatment effects using an event-study specification, a Poisson regression model with high-dimensional fixed effects, and a national interrupted time-series model. The comparison group was restricted to provinces operating traditional PPM models (models 1–4) to ensure comparability of reporting pathways and to avoid structural differences in baseline PPM notification volumes. All models used the same intervention timing, aggregation level, and treatment-period restriction as in the primary analysis.

Event-study estimates showed evidence of gradual divergence in PPM notifications between PPM Model 5 and traditional PPM provinces over longer pre-intervention windows, reflected in rejection of the joint parallel trends test. However, no abrupt changes were observed in the periods immediately preceding implementation, and post-intervention estimates indicated an immediate and sustained increase in PPM-reported notifications. Results from fixed-effects Poisson and interrupted time-series models were directionally consistent. These analyses are presented as robustness checks and were not prespecified as primary outcomes.

**Fig G: Quarterly aggregate public-private-mix-related tuberculosis notifications in intervention and control areas, 2016–2025.**

The figure shows quarterly national tuberculosis notifications reported through PPM channels, stratified by provinces implementing PPM Model 5 and provinces operating traditional PPM models, from 2016 to 2025. Vertical dashed lines indicate the start of national PPM Model 5 rollout and the transition to the post-rollout period. Counts represent aggregated quarterly notifications and are shown for descriptive comparison only.

Quarterly national PPM notifications

|  | PPM Model 5 | Traditional PPM |
| --- | --- | --- |
| 2016 1 | 1401 | 360 |
| 2016 2 | 1429 | 397 |
| 2016 3 | 1686 | 379 |
| 2016 4 | 1270 | 391 |
| 2017 1 | 1591 | 525 |
| 2017 2 | 1599 | 568 |
| 2017 3 | 1883 | 571 |
| 2017 4 | 1512 | 536 |
| 2018 1 | 1218 | 550 |
| 2018 2 | 1204 | 589 |
| 2018 3 | 1131 | 619 |
| 2018 4 | 1286 | 803 |
| 2019 1 | 260 | 501 |
| 2019 2 | 190 | 565 |
| 2019 3 | 2305 | 735 |
| 2019 4 | 1405 | 491 |
| 2020 1 | 1721 | 709 |
| 2020 2 | 1306 | 800 |
| 2020 3 | 1556 | 909 |
| 2020 4 | 1824 | 1294 |
| 2021 1 | 3517 | 782 |
| 2021 2 | 2637 | 801 |
| 2021 3 | 2149 | 696 |
| 2021 4 | 2000 | 788 |
| 2022 1 | 3697 | 560 |
| 2022 2 | 4864 | 905 |
| 2022 3 | 3942 | 889 |
| 2022 4 | 4086 | 851 |
| 2023 1 | 4872 | 1783 |
| 2023 2 | 5417 | 1747 |
| 2023 3 | 6185 | 1557 |
| 2023 4 | 4388 | 1404 |
| 2024 1 | 2424 | 1375 |
| 2024 2 | 2397 | 1258 |
| 2024 3 | 2082 | 1406 |
| 2024 4 | 1991 | 1499 |
| 2025 1 | 1952 | 1077 |
| 2025 2 | 2048 | 1324 |

**Table M: Results of parallel-trend assumption joint tests by pre-treatment time window.**

|  | **df** | **χ²** | ***p*-value** |
| --- | --- | --- | --- |
| 8 quarters (t−8 to t−2) | 7 | 54.21 | **<0.0001** |
| 6 quarters (t−6 to t−2) | 5 | 44.76 | **<0.0001** |
| 4 quarters (t−4 to t−2) | 3 | 5.78 | **0.1230** |

Parallel-trend assumption was assessed using joint Wald tests of pre-intervention event-time coefficients from the event-study specification underlying the main difference-in-differences analysis. Each row reports the χ² test of the null hypothesis that all included pre-intervention coefficients are jointly equal to zero for the indicated pre-intervention window, with degrees of freedom corresponding to the number of leads tested. Results illustrate sensitivity of the joint test to the length of the pre-intervention window.

**Fig H: Event-study estimates of the effect of the intervention on public-private-mix tuberculosis notifications.**

The figure shows the difference-in-differences event-study coefficients comparing provinces implementing PPM Model 5 with provinces operating traditional PPM models (models 1–4), using PPM notifications as the outcome and restricting the sample to the treatment period. Estimates are expressed relative to the quarter immediately preceding intervention launch. Points indicate coefficients and bars denote 95% confidence intervals. Although a joint test rejected parallel trends over longer pre-intervention windows, no abrupt pre-intervention changes were observed, and post-intervention estimates indicate a sustained increase in PPM-reported notifications.

**Table N: Average treatment effect of the PPM Model 5 intervention on public-private-mix-related tuberculosis notifications.**

|  | **ATT (95% CI)** | ***p*-value** | **Treated province-quarters** |
| --- | --- | --- | --- |
| PPM notifications per province-quarter | 332.1 [137.5, 526.7] | 0.001 | 112 |

ATT denotes the average treatment effect on the treated, estimated using a group-time average treatment effects model and restricting the sample to the treatment period. Effects are expressed as PPM-reported TB notifications per treated province–quarter relative to the quarter immediately preceding intervention launch. 95% confidence intervals are shown.

**Table O: Dose-response association between intensity and public-private mix-related tuberculosis notifications.**

|  | **IRR (95% CI)** | ***p*-value** |
| --- | --- | --- |
| Intensity (per 100,000 population) | 1.00070 [1.00034, 1.00105] | <0.001 |
| Post-implementation residual indicator | 0.64657 [0.35024, 1.19360] | 0.163 |

Estimates are from a Poisson regression model with province and quarter fixed effects and a population offset. Screening intensity is defined as the number of persons screened per 100,000 population per quarter. Results are presented as incidence rate ratios with 95% confidence intervals; standard errors are clustered at the province level.

**Table P: National interrupted time-series analysis of public-private-mix-related tuberculosis notifications, 2016–2025.**

|  | **IRR (95% CI)** | ***p*-value** |
| --- | --- | --- |
| **Screening intensity (per 100,000 population)** | **1.00242 [1.00190, 1.00294]** | **<0.001** |
| Linear time trend (t) | 1.02204 [0.99386, 1.05102] | 0.127 |
| Quadratic time trend (t²) | 0.99992 [0.99921, 1.00064] | 0.834 |
| Quarter (ref = Q1) |  |  |
| Q2 | 0.96375 [0.77128, 1.20426] | 0.745 |
| Q3 | 0.93434 [0.77040, 1.13318] | 0.490 |
| Q4 | 0.83083 [0.67932, 1.01615] | 0.071 |
| COVID-19 period indicator | 1.16759 [0.93692, 1.45506] | 0.168 |
| Constant | 0.00002 [0.00001, 0.00003] | <0.001 |

Estimates are from a Poisson regression with a population offset. Screening intensity is defined as the number of persons screened per 100,000 population per quarter. The model includes linear and quadratic secular time trends, quarterly seasonality indicators, and a COVID-19 disruption indicator. Robust standard errors were used.

**Fig I: National interrupted time-series analysis of PPM-reported tuberculosis notifications, 2016–2023.**

The figure shows quarterly national PPM-reported tuberculosis notifications and fitted values from a Poisson interrupted time-series model with a population offset. The model includes linear and quadratic secular time trends, quarterly seasonality indicators, and a COVID-19 disruption indicator.

Quarterly national TB notifications

|  | National | Counterfactual | Fitted |
| --- | --- | --- | --- |
| 2016 1 | 1761 | 1745.58 |  |
| 2016 2 | 1826 | 1724.62 |  |
| 2016 3 | 2065 | 1713.76 |  |
| 2016 4 | 1661 | 1561.73 |  |
| 2017 1 | 2116 | 1925.83 |  |
| 2017 2 | 2167 | 1901.52 |  |
| 2017 3 | 2454 | 1888.38 |  |
| 2017 4 | 2048 | 1719.79 |  |
| 2018 1 | 1768 | 2121.84 |  |
| 2018 2 | 1793 | 2094.25 |  |
| 2018 3 | 1750 | 2078.97 |  |
| 2018 4 | 2089 | 1892.62 |  |
| 2019 1 | 761 | 2330.91 |  |
| 2019 2 | 755 | 2299.06 |  |
| 2019 3 | 3040 | 2280.75 |  |
| 2019 4 | 1896 | 2074.93 | 2074.93 |
| 2020 1 | 2430 | 2553.51 | 2570.62 |
| 2020 2 | 2106 | 2516.89 | 2606.71 |
| 2020 3 | 2465 | 2495.14 | 2843.29 |
| 2020 4 | 3118 | 2268.42 | 2699.58 |
| 2021 1 | 4299 | 3243.92 | 3738.26 |
| 2021 2 | 3438 | 3192.57 | 3516.45 |
| 2021 3 | 2845 | 3160.21 | 3327.28 |
| 2021 4 | 2788 | 2456.97 | 3016.43 |
| 2022 1 | 4257 | 3027.76 | 3866.29 |
| 2022 2 | 5769 | 2979.94 | 4072.99 |
| 2022 3 | 4831 | 2949.85 | 4687.97 |
| 2022 4 | 4937 | 2677.87 | 4435.6 |
| 2023 1 | 6655 | 3278.92 | 5201.34 |
| 2023 2 | 7164 | 3223.02 | 6587.48 |
| 2023 3 | 7742 | 3186.41 | 8408.29 |
| 2023 4 | 5792 | 2888.94 | 7289.72 |
| 2024 1 | 3799 | 3552.67 | 3552.67 |
| 2024 2 | 3655 | 3491.54 |  |
| 2024 3 | 3488 | 3451.32 |  |
| 2024 4 | 3490 | 3128.61 |  |
| 2025 1 | 3029 | 3821.66 |  |
| 2025 2 | 3372 | 3750.37 |  |

# Section C Supplementary Protocol

**Supplementary Protocol**

**PPM Model 5 – An intermediary-facilitated public–private mix intervention for tuberculosis in Viet Nam**

*Implementation period: 2020–2023*

## C.0: About this protocol

This Supplementary Protocol describes the procedures used to operationalize PPM Model 5, an intermediary-facilitated PPM intervention for TB deployed across 15 provinces of Viet Nam between 2020 and 2023. It is intended as a companion to the manuscript and provides the level of operational detail required to support replication, independent appraisal of the intervention, and interpretation of the dose–response and cost analyses reported in the main text.

The document has been prepared from the working Standard Operating Procedures used during implementation by the intermediary agency, Friends for International TB Relief (FIT), in partnership with the NTP and provincial TB programs. It has been rewritten in descriptive form, reorganized by intervention pathway, and edited for an external audience. Procedural content reflects the standard operating procedures as implemented during the evaluation period; principal adaptations made in response to local context or COVID-19 disruptions are noted where relevant. Indicative incentive amounts denominated in VNĐ are stated at the values applied during the implementation period; consolidated cost categories are reported in US Dollars in the main manuscript alongside applicable exchange rates.

The Protocol is organized into seven parts. Part I describes Strategy 1, the referral-based diagnostic pathway implemented through engaged non-NTP healthcare providers. Parts II and III describe two adjacent screening modalities, i.e., screening at non-NTP hospitals and occupational health screening, that operated alongside Strategy 1 with adapted entry-point procedures. Part IV describes Strategy 2, the structured reporting and notification of persons treated for TB in the private sector. Part V describes data systems and quality assurance arrangements common to all strategies. Part VI describes staffing and governance. Part VII summarizes key adaptations during the implementation period.

The protocol primarily describes activities related to provider engagement and case finding up until treatment initiation and notification as originally designed. Ancillary activities layered on top of the core intervention such as activities related to patient support and follow-up during treatment, or operational research activities that were not categorically evaluated in the manuscript have been excluded from this protocol.

## C.1: Strategy 1 – Diagnostic referral through engaged non-NTP providers

### 1. Overview

Strategy 1 engaged non-NTP healthcare providers, i.e., pharmacies, private practices, multi-clinics, hospitals outside the NTP, and occupational health screening providers, etc., as the entry point of a referral-based pathway. Engaged providers conducted standardized verbal symptom assessment of presenting clients, i.e., health-seeking individuals, referred eligible individuals to a subsidized CXR at a contracted radiology facility, and supported molecular diagnostic confirmation through Xpert MTB/RIF (Ultra) testing. Persons with bacteriologically or clinically confirmed TB were linked to NTP-affiliated facilities for treatment initiation and notification.

The intermediary agency, FIT, was responsible for identifying, contracting, training, supervising, and incentivizing participating providers; for coordinating CXR and laboratory services; for ensuring data capture in the digital screening platform, the Access to Care Information System (ACIS); and for supporting linkage to treatment. The NTP supplied diagnostic consumables and equipment, retained ownership of treatment initiation and notification through the Vietnam Tuberculosis Information Management Electronic System (VITIMES), and provided programmatic oversight.

The pathway is summarized in five sequential domains, each addressed in the sections that follow: (i) provider identification and engagement; (ii) symptom assessment and referral; (iii) CXR screening and bacteriological confirmation; (iv) treatment initiation, notification, and follow-up; and (v) result return and provider communication. Cross-cutting data systems and quality assurance arrangements are described in Part V.

### 2. Provider identification and engagement

*2.1 Initiation in implementation provinces*

Implementation in each province was preceded by a structured initiation phase. FIT prepared a detailed implementation plan with provincial activity targets, met with provincial-level health authorities to present the intervention and obtain administrative permission, and held a consensus-building meeting at which an implementation plan was developed and submitted by FIT to the province specifying targets and activities. FIT then prepared and printed referral vouchers and advocacy, communication, and social mobilization materials, verified that ACIS was functional in the new area, and trained field staff on procedures, targets, and reporting deliverables before recruitment of providers commenced.

*2.2 Mapping and recruitment of healthcare providers*

Provincial Departments of Health and District Health Bureaus within District People’s Committees supplied comprehensive lists of private clinics, pharmacies, hospitals, and laboratories operating in each implementation district. These lists formed the master registry of eligible providers, supplemented over time by referrals from already-engaged providers and by active outreach by FIT field officers. Providers were eligible for engagement if they (i) operated in an implementation province, (ii) saw clients with respiratory or TB-suggestive symptoms with sufficient frequency, (iii) were able to commit to participation for a minimum of one year, and (iv) for radiology facilities, possessed a digital or analogue X-ray machine with the capability of printing X-ray images and a designated area suitable for sputum handling. Providers whose specialty made TB-related encounters unlikely (for example, gynecology and oncology practices) were deprioritized. Providers with an established record of community engagement, and women-led practices, were actively prioritized in line with the intervention's equity objectives.

Recruitment was conducted by FIT field officers (PPM Officers) through scheduled visits at which the intervention was introduced, provider concerns were elicited and addressed, and the terms and benefits of participation were presented. District Health Bureaus organized periodic provider meetings that served as additional fora for introduction. Providers who did not commit at the first visit were classified as requiring follow-up and revisited within three to five days; the outcome of each interaction was documented in FIT's customer relationship management system.

*2.3 Collaboration Agreements*

Providers electing to participate signed a Collaboration Agreement (CA) with FIT. Distinct templates were used for the principal provider categories, i.e., clinics without on-site radiography, radiology facilities, pharmacies, hospitals operating outside the NTP with on-site radiography (with and without management-fee support), Xpert-capable laboratories, and occupational health screening companies, to reflect differences in the scope of services provided and the corresponding incentive structures. Each agreement specified its effective period, the activities to be undertaken, the supporting materials and consumables to be supplied by FIT, and the terms of incentive payment. Two countersigned copies were executed; one was retained by the provider and one by FIT. Following execution, providers received their initial supply of referral vouchers, sputum specimen containers, patient information materials, and the list of contracted radiology and laboratory facilities to which clients could be referred.

*2.4 Provider tracking and supervisory contact*

All engaged providers were registered in SPECTRUM, a bespoke customer relationship management system maintained by FIT. SPECTRUM held provider-level information including facility type, contact details, the responsible PPM Officer, the executed CA, and the full record of subsequent interactions. Supervisory visits were classified by purpose (recruitment, training, routine maintenance, problem resolution, surveys, or special events) and by mode (in-person, telephone, or electronic). PPM Officers were required to log each interaction in SPECTRUM; weekly performance reports drew exclusively on logged interactions, and providers without a recorded interaction within three weeks were flagged for follow-up. Interaction records linked back to referral data captured in ACIS, allowing FIT to relate engagement activity to referral output at the provider level and to identify under-performing providers for additional support.

### 3. Symptom assessment and referral

*3.1 Verbal symptom assessment*

Each client presenting at an engaged provider was offered a standardized verbal symptom assessment using the ACIS application installed on the provider's mobile device, or, where electronic screening was not feasible, on a structured paper referral voucher containing equivalent fields. The assessment captured the presence of any one of a defined set of TB-suggestive symptoms (cough, unexpected weight loss, hemoptysis, chest pain, dyspnea fever, or night sweats) and selected risk factors. Eligibility for referral was deliberately broader than the conventional criterion of cough exceeding two weeks: the presence of any one symptom triggered referral, on the rationale that a substantial proportion of persons subsequently diagnosed with TB do not report prolonged cough as their primary complaint.

Each engaged provider was issued a unique ACIS user account, allocated centrally by FIT's monitoring and evaluation team, which linked all subsequent referrals back to the issuing provider for performance monitoring and incentive calculation. PPM Officers supported providers to install the ACIS application, demonstrated its use, and reviewed at least one test entry before live use. Test entries were tagged with a reserved voucher identifier to allow FIT's monitoring and evaluation team to remove them prior to analysis.

*3.2 Issuance of the referral voucher*

Clients eligible after symptom assessment received a referral voucher entitling them to a subsidized chest radiograph at any contracted radiology facility, together with a transportation stipend (CXR subsidy: VNĐ 65,400; transport stipend: VNĐ 50,000). The subsidy was set to cover the standard CXR fee at most contracted facilities; clients attending facilities charging in excess of the standard fee were responsible for the difference, and this was disclosed at the point of referral. Vouchers were single-use, were redeemable only at contracted facilities, were non-transferable, and could not be exchanged for cash. Each voucher carried a unique identifier that linked the referral, in ACIS, to the issuing provider, the screened individual, and any subsequent CXR and laboratory results.

*3.3 Referrals issued without electronic screening*

Where ACIS was temporarily unavailable, or where a provider did not adopt the platform but generated substantive referral volume, paper-only referral was permitted. The voucher contained a parallel set of mandatory fields capturing the data otherwise entered into ACIS, including the provider's name, signature or facility stamp, the client's name and date of referral, and the symptom assessment responses. These fields were subsequently digitized by FIT's data team and uploaded to ACIS. Paper-only referral was monitored as an exception pathway, with the expectation that providers using it routinely would be supported to transition to electronic screening.

### 4. CXR screening and bacteriological confirmation

*4.1 Diagnostic algorithm*

Clients presenting at a contracted radiology facility with a valid voucher received a digital chest radiograph; the standard radiographic report and image were provided to the client according to the facility's usual practice. Radiographs were read and classified by the radiology facility, and the result was recorded on the back of the voucher together with the date of imaging and the facility's signature or stamp. Vouchers without complete data on the radiology side were not eligible for reimbursement, and PPM Officers reconciled missing fields with the facility before transport.

All clients with a TB-suggestive abnormal radiograph were eligible for molecular testing using Xpert MTB/RIF (Ultra), reflecting the NTP's "Double-X" algorithm. In a small proportion of cases, clients with normal or absent radiographs but elevated risk on other grounds (for example, HIV co-infection, documented exposure to active drug-resistant TB disease) were also referred for testing. Where Xpert testing were unavailable, whether due to lack of equipment or the temporary stockout of Xpert cartridges, sputum smear microscopy was used in its place and the client was tracked for confirmatory Xpert testing once supply was restored. Clients who declined CXR but met the NTP's standard criteria were eligible for sputum smear microscopy as an alternative initial test.

Persons with positive Xpert results without rifampicin resistance were referred for first-line treatment initiation. Persons with rifampicin-resistant results were referred to provincial-level lung hospitals for second-line evaluation and treatment in accordance with the NTP's programmatic management of drug-resistant TB guidance. Persons with TB-suggestive radiographs but negative bacteriologic test results were treated with broad-spectrum antibiotics and reviewed after two to four weeks; those with persistent radiographic abnormalities were referred to a clinical review panel, which determined whether to initiate treatment on clinical grounds in accordance with NTP guidelines. A subsidized follow-up radiograph was provided for this clinical diagnosis pathway.

*4.2 Sputum collection*

Sputum collection was performed either at the radiology facility or at the client's home, with the choice driven by clinical and logistical considerations. For on-site collection, healthcare workers explained the purpose of the sample, demonstrated the technique using a standardized patient information poster or pamphlet, and supervised collection in a designated, ventilated space. Sample adequacy was assessed before the client departed; inadequate samples (predominantly saliva, or insufficient volume) prompted a repeat attempt, with adjuncts including back-percussion or aerosolized saline where required and available (provided by the study to the extent requested and possible). For home collection, clients were issued a labelled specimen tube and instructed in early-morning collection technique, including oral hygiene preparation, and either returned the sample to the radiology facility or surrendered it to a PPM Officer. All sputum tubes were labelled with the client's identifier prior to collection. A standard incentive of VNĐ 10,000 was payable for sputum collection, accruing to the radiology facility for on-site collection or to the PPM Officer for off-site collection.

*4.3 Sputum transport*

Specimens were transported to contracted Xpert laboratories in three-layer packaging (one paper layer and two plastic layers) consistent with national infectious-substance handling guidance, accompanied by a sputum transport manifest listing the number of tubes, individual tube identifiers, demographic details, and date of dispatch. Specimens were transported upright, with appropriate hazard labelling, and arrived at the laboratory within 48 hours of collection. Each transfer was recorded in ACIS prior to arrival at the laboratory; specimens not registered against a transport manifest were not eligible to trigger sputum-collection incentive payments. On laboratory receipt, sample condition was verified, the date and time of receipt were countersigned by laboratory staff, and the manifest was retained. Transport incentives were tiered by distance to the receiving laboratory (VNĐ 30,000 for journeys under 20 km; VNĐ 70,000 for journeys of 20 km or more); shipments handled by contracted specimen transport services were billed at city-specific agreed rates.

*4.4 Laboratory testing and result return*

Xpert testing was performed within 48 hours of receipt at the assigned laboratory. All samples referred under the intervention were marked accordingly in the laboratory register to allow weekly reconciliation against FIT records. Samples from clients with TB-suggestive radiographs were tested directly on Xpert. For samples with a positive sputum smear, a confirmatory Xpert test was performed; in cases of smear-positive, Xpert-negative discordance with a normal or absent radiograph, a repeat Xpert test was performed, and the consolidated result was used for clinical decision-making.

Test results were returned to PPM Officers from the laboratory as a signed paper report countersigned by the laboratory manager, and were entered into ACIS against the client's record. Each laboratory provided a monthly listing of all tests performed under the intervention to FIT's monitoring and evaluation team, which reconciled the listing against ACIS records to confirm completeness.

### 5. Result return and provider communication

Where the test result was positive for drug-sensitive TB, the PPM Officer notified the referring provider by telephone, logged the call in SPECTRUM, and returned a signed paper report bearing the patient's information, the requesting clinician's signature, and the laboratory's authentication. The paper result served as documentation for treatment initiation. Where the result was rifampicin-resistant, the PPM Officer immediately notified the referring provider with instructions to refer the client to the public sector for programmatic management of drug-resistant TB.

In some instances, the referring clinician requested that the PPM Officer contact the client directly using the telephone number recorded on the referral voucher, asking the client to return to the clinic for re-examination and result collection. Such calls were placed only after the clinic had been informed and were logged in SPECTRUM. Where the client could not be reached on the first attempt, at least two further attempts were made. Ultimate responsibility for communicating results to the patient rested with the referring provider, as specified in the CA. For positive results originating from referrals by pharmacies, the PPM Officer contacted both the pharmacist and the client and supported linkage to an appropriate diagnostic or treatment facility, with the pharmacist's consent.

### 6. Treatment initiation, notification, and follow-up

Persons with confirmed TB were counselled on treatment options and supported to initiate treatment at an NTP-affiliated facility. ACIS recorded the date and place of treatment initiation, the regimen prescribed, and the expected duration. For clients initiating treatment in the public sector, treatment-outcome data were extracted from VITIMES through the client's unique ACIS identifier. For clients electing private-sector treatment, PPM Officers contacted the treating provider monthly to update treatment status; FIT remained accountable for capturing and reporting final outcomes to the PPM Manager, who consolidated outcomes for the managed districts at the end of the six-month treatment course.

All bacteriologically confirmed cases were notified to the NTP. Drug-resistant cases were referred to the designated provincial lung hospital for specialist management, and the PPM Officer continued follow-up at the District TB Unit or commune health station. Treatment outcomes were classified per Ministry of Health guidance (cured, completed, failed, lost to follow-up, transferred out, died). PPM Officers also prompted treating providers to initiate household contact investigation for confirmed cases, in accordance with NTP guidance.

## C.2: Screening at non-NTP hospitals

### 7. Scope and entry points

In parallel with Strategy 1's primary referral pathway through ambulatory non-NTP providers, FIT operated a structured screening modality at hospital departments outside the NTP whose case mix included a substantial proportion of clients presenting with respiratory complaints. Engaged departments included respiratory disease clinics; infectious and tropical disease centers; ear, nose and throat departments; and pediatric departments at hospitals and clinics in the implementation areas. The aim was to capture symptomatic clients at a clinical entry point at which TB might otherwise not be considered, and to channel them into the same diagnostic algorithm used in Strategy 1.

Screening at participating hospitals was undertaken by departmental healthcare workers nominated by hospital leadership, with operational support from FIT PPM Officers and PPM Managers responsible for the intervention area. Hospitals participated under a CA specifying the screening departments, the nominated screening staff, the supporting materials and consumables to be provided by FIT, the procedures for sputum handling and transport, and the terms of incentive payment.

### 8. Screening procedures

All clients presenting to engaged departments were eligible for symptom screening, with priority given to those with respiratory complaints. Healthcare workers conducted symptom assessment using the ACIS application, applying the same symptom criteria as in Strategy 1 (cough, unexpected weight loss, hemoptysis, chest pain, dyspnea fever, or night sweats). Screening staff were responsible for ensuring that responses were complete and accurate. Each screening event recorded in ACIS attracted an incentive of VNĐ 10,000 payable to the screening healthcare worker.

Clients meeting symptom criteria were referred to the hospital's chest X-ray facility. Voucher-handover and reimbursement arrangements were established by each hospital in accordance with the CA; in most participating hospitals, vouchers were handed by screening staff to chest X-ray department staff at the end of each working day. Clients with normal radiographs were counselled to return for re-examination after one to two months. Clients with TB-suggestive abnormal radiographs were referred to Xpert testing or, where clinically indicated and at the treating clinician’s choice in consultation with the patient, to alternative tests such as smear or culture. The cost of Xpert testing was met from the project budget; clients electing alternative tests not covered by the project bore the cost out-of-pocket or through health insurance reimbursement, and were counselled by the treating clinician about the implications of this choice. The treating clinician was instructed to recommend Xpert as the first-line confirmatory test, however, hospital standard operating procedures and diagnostic algorithms were respected.

### 9. Sputum collection and transport

Sputum collection at engaged hospitals followed the same standardized technique described in Strategy 1, with on-site collection at the chest X-ray facility where feasible, and labelled-tube home collection for clients unable to produce a sample on-site, with the sample retrieved on the following day. Hospital healthcare workers notified FIT PPM Officers of completed collections at the end of each working day; collected samples were transported to the contracted Xpert laboratory by 16:30 the same day, in three-layer packaging consistent with national infectious-substance handling guidance.

In major cities, rapid shipping providers were engaged for batch collection and transport from large-volume facilities to Xpert laboratories. These providers were contractually engaged and their staff sufficiently trained in sample handling and infection control. Recruitment, coordination, and payments of these providers were managed by FIT and participating healthcare facilities. The NTP’s National Reference Laboratory staff provided initial and refresher trainings to providers, as needed.

### 10. Diagnostic interpretation and treatment linkage

Clients with positive bacteriology by Xpert, sputum smear, or mycobacterial culture were initiated on TB treatment. Clients with Xpert-positive, rifampicin-sensitive results were initiated on first-line treatment, and the treating healthcare worker counselled the client on whether treatment was best initiated at the District TB Unit or at a provincial lung hospital. Clients with Xpert-positive, rifampicin-resistant results were managed in accordance with the NTP's programmatic management of drug-resistant TB guidance. Clients with TB-suggestive radiographs but negative bacteriology were treated with broad-spectrum antibiotics and re-evaluated after two to four weeks; persistent radiographic abnormalities prompted referral to a clinical diagnostic board, with treatment initiated on clinical grounds where indicated. A subsidized follow-up radiograph was provided for this pathway.

## C.3: Occupational health screening

### 11. Scope and engagement

Occupational health screening was operated as a third entry-point modality, targeting employees of companies with on-site health departments in the implementation areas or businesses providing occupational health screening services to companies complying with statutory requirements on workforce wellbeing. The aim was to capture symptomatic individuals through routine workplace healthcare encounters and to integrate TB screening into existing occupational health activities. Engagement was led by FIT PPM Officers, who approached company health departments and health screening providers to introduce the intervention's purpose, activities, and benefits to workers, the service provider business models, and company health staff. Companies electing to participate signed a CA with FIT specifying the screening modality (see below), the supporting materials and consumables to be provided, and the terms of incentive payment.

### 12. Screening modalities

Two complementary modalities were used, reflecting differences in the routine occupational health practice of participating companies.

### *12.1 Modality A: Symptom-driven referral*

Under Modality A, healthcare staff at engaged companies followed the standard Strategy 1 symptom-screening and referral procedure. Where an employee reported any of the index symptoms (cough, unexpected weight loss, hemoptysis, chest pain, dyspnea fever, or night sweats), the healthcare worker completed a referral voucher entitling the employee to a subsidized CXR at the nearest contracted radiology facility. Each completed referral with a CXR result recorded in ACIS attracted an incentive of VNĐ 20,000 payable to the company healthcare worker. Clients with normal radiographs were counselled to return for re-examination after one to two months, and clients with TB-suggestive radiographs entered the standard Xpert pathway.

*12.2 Modality B: Annual occupational health check integration*

Under Modality B, screening was integrated into the company's annual or service provider’s periodic occupational health check. Where a chest radiograph was already part of the company's standard health check, performed either by an external X-ray provider or by the company's own facility, FIT coordinators worked with company/provider healthcare staff to identify employees with TB-suggestive abnormal radiographs from the routine check. These employees were issued a labelled sputum tube and instructed in early-morning home collection. Samples were returned to the company health department on the following working day and collected by FIT PPM Officers for transport to the contracted Xpert laboratory. Each abnormal radiograph recorded in ACIS attracted an incentive of VNĐ 10,000, and standard sputum-collection incentives were applied. Where alternative tests were preferred over Xpert by the employee, the alternative tests were paid out-of-pocket; the cost of Xpert was met from the project budget.

### 13. Sputum handling and treatment linkage

Sputum collection, packaging, and transport followed the procedures described in Strategy 1, with on-site collection at the contracted radiology facility where the employee attended, or labelled-tube home collection with retrieval through the company health department on the following working day. Diagnostic interpretation and treatment-linkage rules were identical to Strategy 1: rifampicin-sensitive disease was initiated on first-line treatment, with the company healthcare worker counselling the employee on whether treatment was best initiated at the District TB Unit or at a provincial lung hospital; rifampicin-resistant disease was managed in accordance with the NTP's programmatic management of drug-resistant TB guidance; and persistent TB-suggestive radiographs with negative bacteriology entered the broad-spectrum antibiotic re-evaluation pathway with subsidized follow-up imaging.

## C.4: Strategy 2 – Notification of privately treated TB

### 14. Scope and rationale

Strategy 2 addressed the underreporting of TB cases treated entirely within the private sector, which had been identified in Viet Nam as a substantial component of the national treatment coverage gap. Engaged providers were asked to disclose to FIT the full set of clients they were privately treating for TB, irrespective of the form of disease (pulmonary or extrapulmonary), treatment history (new or retreatment), drug-resistance status (drug-sensitive, drug-resistant, or latent infection), or whether the provider's diagnostic and treatment practice followed NTP procedures. Strategy 2 thereby created a verified channel through which privately delivered TB care could be brought into the national notification system, while simultaneously creating the operational basis for FIT to support quality assurance, treatment adherence, and outcome reporting for the private cohort.

### 15. Reporting procedures

PPM Officers visited participating private providers weekly to collect treatment data, using a structured web-based reporting tool. Providers were asked to disclose, for each privately treated client, a defined set of variables meeting the NTP’s minimum reporting standard, covering identity (name, age, sex, contact details, address), clinical presentation (form of TB, justification for treatment), and treatment (start date, regimen, expected duration). Providers could choose to enter data directly into the web tool, or to provide it on paper or electronic spreadsheet for entry by the PPM Officer. Adjunct clinical data, e.g., sputum results, prior radiography, etc., were recorded where available, but identity and treatment fields constituted the minimum required disclosure.

Each week, FIT's monitoring and evaluation team produced a completeness report listing partial disclosures and missing fields, allowing PPM Officers to return to providers to complete records and to verify clients in line with the procedures described below. Confirmed treatment data, validated against duplicate records held in VITIMES and provincial sources, formed the basis for incentive payments to providers.

### 16. Verification of disclosed clients

Not all privately disclosed clients were eligible for notification to VITIMES. To meet the NTP's case definition, a client had to be either bacteriologically confirmed or clinically diagnosed in line with the NTP's clinical algorithm, and had to be a previously unnotified case. FIT therefore implemented a structured verification cascade prior to notification, with three components.

*16.1 Bacteriological verification through Xpert testing*

For each newly disclosed client (defined as having been on treatment for less than two weeks), FIT's monitoring and evaluation team produced a verification list. PPM Officers contacted the client using the contact details supplied by the provider to arrange sputum collection. Where the provider preferred not to have FIT contact clients directly, sputum was collected at the provider's premises and transported by the PPM Officer for testing. In a small number of cases, providers retained complete responsibility for collection, with FIT supporting transport only. Direct Xpert testing of clients on private treatment was time-limited (less than one month from treatment start) to minimize the risk of bacteriological negativity attributable to treatment exposure rather than absence of disease; where the provider suspected TB in a client not yet on treatment, that client was instead routed through the standard CXR-led Strategy 1 pathway.

*16.2 Telephone verification*

All clients reported as initiating private TB treatment were contacted by a PPM Officer during the first month of treatment. The telephone contact had four purposes: confirming the client's existence as a real individual, identifying clinical or logistical issues warranting the treating provider's attention, reinforcing treatment adherence and the importance of completion, and offering participation in operational research surveys. Calls additionally allowed verification of selected reported data, e.g., comparing reported regimen against the medication the client described receiving. Calls were logged centrally.

FIT's monitoring and evaluation team monitored the proportion of disclosed clients reachable by telephone at the provider level. Clients with missing contact numbers, incorrect numbers, or unreachable status after three contact attempts were aggregated to provider-level indicators, which were reviewed by FIT management to identify providers requiring additional support, additional supervision, or, in rare cases, suspension from the verification pathway. Where a provider declined to permit FIT direct contact with their clients, the local PPM Manager visited the provider to discuss the importance of independent verification before notification, and the outcome was logged in SPECTRUM.

As an alternative to direct phone verification, PPM Officers used Zalo, a widespread chat application in Viet Nam for indirect verification. Specifically, patient phone numbers were used to search for Zalo profiles, which commonly included names, province and district of residence. Based on this information, an indirect verification could be completed and marked as such for the privately treated individual.

*16.3 Cross-system reconciliation*

Disclosed client identifiers were searched against VITIMES and against district paper treatment registers (and provincial lung hospital registers, if applicable) to identify clients already notified through other channels. Where a match was found and the prior notification was not consistent with disease relapse, the client was recorded as already notified, the treatment information was retained in the project database, and the client was excluded from FIT-led notification to avoid double-counting.

### 17. Notification through VITIMES

Notification of private-sector clients to VITIMES was performed once per quarter, in the final week of the quarter, through FIT's private-sector basic management unit (BMU) account, or through the provincial TB program’s BMU account as per mutual agreement. Only verified clients, i.e., those who had had a documented in-person encounter with FIT staff, typically for sputum collection, or had been confirmed by telephone, and who were not previously notified, were eligible. Clients meeting the NTP's bacteriological case definition were notified without further review. Clients who had been clinically diagnosed by a process that did not fully align with NTP guidance were referred for review by FIT and, where appropriate, the NTP's PPM team prior to notification.

FIT exercised caution in selecting clients for notification, recognizing that notification triggered an obligation to report treatment outcomes and that adverse outcomes in the privately treated cohort would be aggregated into national program indicators. All disclosed treatments were retained in project databases for project monitoring and donor reporting; only the verified subset was notified. Bulk upload of verified records to VITIMES was operationalized in collaboration with the NTP's VITIMES team in preference to individual record creation.

### 18. Patient monitoring and treatment support

FIT's monitoring and evaluation team produced regular lists of privately treated clients due for follow-up activities, including monthly smear monitoring, regimen transition, and end-of-treatment outcome capture. PPM Officers used these lists to discuss treatment progress with the provider and to update the reporting tool. Where a client ceased visiting the provider before the expected end of treatment, the PPM Officer attempted telephone contact to counsel the client on returning to treatment; where telephone contact failed, a home visit was conducted to support re-engagement. FIT aimed to contribute to successful case management, but the private provider retained responsibility for ensuring treatment success for the cohort of clients reported as initiating private treatment, and particularly for those formally notified through VITIMES, with treatment success rates monitored alongside notification volumes as core program indicators.

## C.5: Data systems and quality assurance

### 19. Data systems

Three principal information systems supported the intervention. ACIS, a digital platform managed jointly with the NTP, captured symptom assessment, CXR referral, sputum testing, and treatment-initiation data at the individual level, including a unique client identifier used to link records across the cascade. SPECTRUM, a customer relationship management system maintained by FIT, captured provider-level data including facility profiles, executed CAs, the full record of supervisory and recruitment interactions, and provider-level incentive eligibility. VITIMES, the NTP's routine surveillance system, was the system of record for notified TB cases and treatment outcomes; private-sector notifications under Strategy 2 were uploaded through FIT's BMU account, and treatment outcomes for ACIS-identified Strategy 1 clients in public-sector treatment were extracted from VITIMES through the ACIS identifier.

User accounts on each system were administered by the relevant data team, with role-based access reflecting the user's responsibilities. Each engaged provider received a unique ACIS user identifier issued centrally; multi-clinics and PPM hospitals were registered at facility level with nested accounts for individual practitioners where applicable, ensuring that referrals and notifications could be attributed at the appropriate level.

### 20. Voucher reconciliation and digitization

Vouchers were collected from contracted radiology facilities on a weekly cycle, allowing same-week digitization. At collection, PPM Officers verified completeness of the radiology entries; incomplete vouchers were reconciled with the facility before transport. Vouchers were grouped by collecting facility and collection date, counted, recorded against an electronic voucher-tracking sheet, and digitized by a dedicated data entry coordinator using web-based forms in ONA. Data entry was completed within two days of vouchers arriving at the FIT office. The monitoring and evaluation team reviewed digitized entries against the source vouchers via spot-check, and validated entries were uploaded to ACIS in batch. Source vouchers were stored in chronological order in labelled containers in locked cabinets, allowing retrieval by collection date and facility.

### 21. Performance-based incentive structure

Incentives were structured to reward complete, verifiable contributions across the referral and notification pathways rather than recruitment alone. Indicative payment categories included symptom assessment recorded in ACIS, completed CXR referral with results documented, sputum collection at engaged facilities, sputum transport (banded by distance to laboratory), and successful linkage to treatment for Strategy 1; and disclosure of privately treated clients with verified, non-duplicated records for Strategy 2. Indicative incentive amounts denominated in VNĐ are stated within the relevant procedural sections; consolidated incentive expenditure is reported in the manuscript's micro-costing analysis.

Reimbursements to participating providers were settled monthly against physical (e.g., vouchers) and electronic records reconciled by the field team and signed off by the provider, with payment within four weeks of the following month. Incentive eligibility was contingent on data being recorded in ACIS or, for paper-only referrals, on full reconciliation against ACIS; activities not captured in ACIS or SPECTRUM were not eligible for payment. FIT retained discretion to suspend incentive eligibility where verification monitoring raised concerns, pending review by FIT management.

FIT’s finance and accounting department managed payments to individual providers in accordance to the payee information provided in the CA. Facilities provided official tax receipts (i.e., Value Added Tax), if available, for verification of payment receipt. For providers unable or unwilling to furnish a tax receipt, FIT automatically withheld 10% contractor income tax, which was reported and paid to tax authorities quarterly for all eligible providers. At year-end, FIT shared income-related reports to the tax authorities on behalf of all providers.

### 22. Reporting cadence

Field activity, recruitment progress, referral volume, and screening output were reported weekly. SPECTRUM interactions were the basis of weekly field-activity reports; interactions logged after the Thursday 23:59 cut-off were excluded from that week's report. Bacteriological yield, treatment initiation, and notification activity were reviewed monthly. Cohort treatment outcomes were finalized at the conclusion of each treatment course (six months for drug-sensitive disease; longer for drug-resistant disease) and incorporated into quarterly performance reviews with the NTP. Data quality reviews were conducted on a monthly cycle by the monitoring and evaluation team, and findings were fed back to field teams and to providers as part of routine supervision.

## C.6: Staffing and governance

### 23. Staffing structure

Implementation staff were recruited, trained, managed, and remunerated by FIT, with each staff member reporting to a designated line manager. Three teams supported implementation: a program team comprising area budget owners, PPM Managers, and PPM Officers; a monitoring, evaluation, and research team comprising a research director, monitoring and evaluation managers, and monitoring and evaluation officers; and an administration and finance team comprising a finance manager, administration and finance officers, and an administrative officer.

### 24. Role of the PPM Manager

The PPM Manager held the principal operational responsibility for the intervention within an assigned geography. The role encompassed relationship management with engaged public and private providers, including troubleshooting and resolving issues raised by providers, continuously refining engagement strategies, and supervising the distribution of provider incentives. The PPM Manager directly recruited and supervised three to four PPM Officers, set the operating norms of the team, mentored Officers in relationship management, and reviewed SPECTRUM entries to provide regular feedback on output and quality of work.

The PPM Manager additionally supervised field implementation across the full provider mix, i.e., clinics, pharmacies, hospitals outside the NTP, occupational health screening companies, and X-ray facilities, with the operational objectives of maximizing referrals of presumptive TB cases, ensuring that referred cases received appropriate diagnostic testing, supervising data collection from public-sector facilities, coordinating registration of TB cases identified in the private sector, supervising patient enrolment, and enhancing case finding in the private sector. The role included monitoring and evaluation responsibilities, e.g., laboratory error-rate monitoring, verification of community-collected referral and notification data, collaboration with the monitoring and evaluation team to resolve data issues, feedback on ACIS PPM modules, coordination of refresher training in electronic data tools, and capacity building of PPM Officers in data collection, and routine reporting outputs including weekly progress presentations and quarterly narrative reports for donors. PPM Managers also supported procurement of project supplies and other operational tasks as required.

Area budget owners acted as supervisors of PPM Managers with similar field responsibilities for high-priority providers. In addition, budget owners fulfilled project management, finance and accounting and progress reporting to NTP and funders.

### 25. Role of the PPM Officer

The PPM Officer was the principal field-level role, with responsibilities organized into five domains.

First, network development and management: building and maintaining relationships with private providers and clinics, expanding the engaged network across all provider categories, updating SPECTRUM with completed activities and provider concerns, troubleshooting at the provider level while escalating issues to the PPM Manager, and ensuring that engaged clinics were stocked with project promotional materials.

Second, facilitation of TB testing: tracking screening data through ACIS PPM modules, stimulating referral activity at lower-performing clinics and sustaining engagement at higher-performing clinics, facilitating engagement and referral through targeted social network outreach to private-sector pharmacists, supporting the PPM Manager in establishing screening and referral from hospitals outside the NTP's network, coordinating with occupational health screening companies on abnormal CXR follow-up, collecting CXR vouchers and results from radiology facilities, entering CXR data into ACIS, following Ministry of Health guidance on sputum collection and transport, transporting sputum samples to public-sector laboratories, and informing testing results to clients and their providers.

Third, support to private-sector notification: collecting notification data from providers treating TB privately under Strategy 2, collecting confirmatory sputum samples from privately treated clients and delivering them to public-sector laboratories, entering disclosed data and laboratory results into the relevant electronic systems, and supporting verification of disclosed data in collaboration with the monitoring and evaluation team.

Fourth, patient support: assisting clients in registering at the District TB Unit where treatment was initiated in the public sector, providing educational materials to clients and their providers to improve disease understanding, and linking clients to appropriate counselling resources where questions or concerns arose. Privately treated patients reported by the treating provider as facing particular economic hardship were eligible for cash transfers categorized into vitamin and nutrition support. PPM Officer facilitated the documentation and approval process for these cash transfers.

Fifth, support to operational research: supporting clients to register for relevant research protocols, coordinating with the monitoring, evaluation, and research team in introducing research protocols to providers and securing their participation, and conducting structured surveys and feedback collection. PPM Officers additionally submitted weekly reports to the PPM Manager, participated in weekly progress meetings, supported quarterly networking events for providers, and supported other aspects of project implementation as required.

### 26. Reporting structure

Within each implementation province, PPM Officers reported to a designated PPM Manager. PPM Managers in turn reported to the area budget owner, with technical line accountability to the relevant central FIT functions for monitoring and evaluation, finance, and program management. The monitoring and evaluation team operated as a horizontal function across implementation areas, with direct lines of communication to PPM Managers and PPM Officers for data quality issues and to FIT senior management for aggregated reporting.

## C.7: Adaptations during the implementation period

### 27. COVID-19 adaptations

Between Q1 and Q3 2021, peak COVID-19 disruptions in Viet Nam constrained in-person provider engagement and facility-based clinical activity in many implementation provinces. FIT temporarily replaced in-person provider visits with remote engagement (telephone and video) where required by public health restrictions, and expanded home-based sputum collection relative to facility-based collection. Voucher reconciliation cycles were preserved on a weekly cadence where possible, with adjustments to courier arrangements to accommodate movement restrictions. Verification calls under Strategy 2 continued without interruption, given that the modality was already remote. These adaptations are reflected in the dose–response analyses through the implementation-intensity exposure variable, which captures changes in the volume of recorded screening activity over the implementation period.

### 28. Expansion of provider categories and intervention components over time

The intervention's scope of engaged provider categories as well as intervention components expanded over the implementation period as additional CA templates were developed, and additional activities, particularly in relation to patient support, were added. These were included if they comprised effect modifiers of TB notifications and excluded otherwise.

The most substantive included additions concerned occupational health screening providers and hospitals outside the NTP with on-site radiography (with and without management-fee support). The procedures for symptom assessment, CXR screening, and laboratory testing remained constant across these expansions, while upstream engagement, voucher-handover, and incentive arrangements were tailored to provider type. Where modality-specific procedures differ from the standard Strategy 1 pathway, those differences are described in Parts II and III.

### 29. Operational refinements

A number of incremental procedural refinements were introduced during implementation in response to programmatic learning. These included the introduction of bulk upload of verified Strategy 2 records to VITIMES in preference to individual record creation; the standardization of the verification cascade described in Section 16; the formalization of a follow-up subsidized radiograph for clients in the broad-spectrum antibiotic re-evaluation pathway; and the progressive integration of provider-level performance feedback into supervisory cycles. None of these refinements altered the core diagnostic algorithm, the eligibility criteria for incentives, or the criteria for notification.
