# Supplementary material for: Evaluation of the tuberculosis notification impact from an intermediary-facilitated public–private mix intervention in Viet Nam: A population-level quasi-experimental study
Source: PLoS Med. 2026 Sep 23;23(9):e1005144. doi: 10.1371/journal.pmed.1005144 (PMC13600524; doi:10.1371/journal.pmed.1005144)
Supplement: Supplementary file 2 [file pmed.1005144.s002.docx]

STROBE checklist

*The STROBE checklist is reproduced from von Elm E, Altman DG, Egger M, Pocock SJ, Gøtzsche PC, Vandenbroucke JP; STROBE Initiative. The Strengthening the Reporting of Observational Studies in Epidemiology (STROBE) statement: guidelines for reporting observational studies. PLoS Med. 2007;4(10):e296. https://doi.org/10.1371/journal.pmed.0040296. Licensed under CC BY 4.0 (https://creativecommons.org/licenses/by/4.0/). Study-specific reporting locations have been added to the original checklist.*

|  | Item No | Recommendation | Section & paragraph # |  |
| --- | --- | --- | --- | --- |
| **Title and abstract** | 1 | (*a*) Indicate the study’s design with a commonly used term in the title or the abstract | - Abstract: 1 | |
|  |  | (*b*) Provide in the abstract an informative and balanced summary of what was done and what was found | - Abstract: 1 | |
| Introduction | | |  | |
| Background/rationale | 2 | Explain the scientific background and rationale for the investigation being reported | - Background: 4 | |
| Objectives | 3 | State specific objectives, including any prespecified hypotheses | - Methods, Study aims and design: 1 | |
| Methods | | |  | |
| Study design | 4 | Present key elements of study design early in the paper | - Methods, Study aims and design: 1 | |
| Setting | 5 | Describe the setting, locations, and relevant dates, including periods of recruitment, exposure, follow-up, and data collection | - Methods, Study aims and design: 2 Appendix: S1-10 | |
| Participants | 6 | (*a*) Give the eligibility criteria, and the sources and methods of selection of participants. Describe methods of follow-up | - Methods, Intervention: 1 - Appendix: S1-1 | |
|  |  | (*b*) For matched studies, give matching criteria and number of exposed and unexposed | - N/A | |
| Variables | 7 | Clearly define all outcomes, exposures, predictors, potential confounders, and effect modifiers. Give diagnostic criteria, if applicable | - Methods, Outcomes: 1 - Methods, Statistical analyses: 2–5 | |
| Data sources/ measurement | 8* | For each variable of interest, give sources of data and details of methods of assessment (measurement). Describe comparability of assessment methods if there is more than one group | - Methods, Intervention: 4  Appendix: S1-3 | |
| Bias | 9 | Describe any efforts to address potential sources of bias | - Methods, Statistical analyses: 2–5  Appendix: S1-3 to S1-7 | |
| Study size | 10 | Explain how the study size was arrived at | - Methods, Statistical analyses: 1 | |
| Quantitative variables | 11 | Explain how quantitative variables were handled in the analyses. If applicable, describe which groupings were chosen and why | - Methods, Statistical analyses: 1–5  Appendix: S1-3 to S1-8 | |
| Statistical methods | 12 | (*a*) Describe all statistical methods, including those used to control for confounding | - Methods, Statistical analyses: 1–5 Appendix: S1-3 to S1-8 | |
|  |  | (*b*) Describe any methods used to examine subgroups and interactions | - Methods, Statistical analyses: 1–5 Appendix: S1-3 to S1-8, S2-6 | |
|  |  | (*c*) Explain how missing data were addressed | - Appendix: S2-2 | |
|  |  | (*d*) If applicable, explain how loss to follow-up was addressed | - Appendix: S1-2, S3 | |
|  |  | (*e*) Describe any sensitivity analyses | - Appendix: S1-5 to S1-7, S2-6 | |
| Results | | |  | |
| Participants | 13* | (a) Report numbers of individuals at each stage of study—eg numbers potentially eligible, examined for eligibility, confirmed eligible, included in the study, completing follow-up, and analysed | - Results, Provider recruitment and participation: 1 - Appendix: S2-2 | |
|  |  | (b) Give reasons for non-participation at each stage | - Results, Provider recruitment and participation: 1 Appendix: S2-2 | |
|  |  | (c) Consider use of a flow diagram | - Appendix: S2-2 | |
| Descriptive data | 14* | (a) Give characteristics of study participants (eg demographic, clinical, social) and information on exposures and potential confounders | - Appendix: S2-1–S2-2 | |
|  |  | (b) Indicate number of participants with missing data for each variable of interest | - Appendix: S2-1–S2-2 | |
|  |  | (c) Summarise follow-up time (eg, average and total amount) | - Methods, Study aims and design: 3 | |
| Outcome data | 15* | Report numbers of outcome events or summary measures over time | - Results, Provider recruitment and participation: 1 - Results, TB care cascade by strategy: 1 | |
| Main results | 16 | (*a*) Give unadjusted estimates and, if applicable, confounder-adjusted estimates and their precision (eg, 95% confidence interval). Make clear which confounders were adjusted for and why they were included | - Results, Effect on TB notifications: 1 - Results, Dose-response between intensity and TB notifications: 1 - Results, Trend in national TB notifications: 1 | |
|  |  | (*b*) Report category boundaries when continuous variables were categorized | - Appendix: S2-2 | |
|  |  | (*c*) If relevant, consider translating estimates of relative risk into absolute risk for a meaningful time period | - Results, Effect on TB notifications: 1 - Results, Dose-response between intensity and TB notifications: 1 - Results, Trend in national TB notifications: 1 | |
| Other analyses | 17 | Report other analyses done—eg analyses of subgroups and interactions, and sensitivity analyses | - Results, Intermediate outcome: PPM notifications: 1 - Results, Marginal costs per additional TB notification: 1 Appendix: S1-5 to S1-7, S2-6 | |
| Discussion | | |  | |
| Key results | 18 | Summarise key results with reference to study objectives | - Discussion: 1 | |
| Limitations | 19 | Discuss limitations of the study, taking into account sources of potential bias or imprecision. Discuss both direction and magnitude of any potential bias | - Discussion: 11 Appendix: S1-5 to S1-7 | |
| Interpretation | 20 | Give a cautious overall interpretation of results considering objectives, limitations, multiplicity of analyses, results from similar studies, and other relevant evidence | - Discussion: 2–10 | |
| Generalisability | 21 | Discuss the generalisability (external validity) of the study results | - Discussion: 5, 10–11 | |
| Other information | | |  | |
| Funding | 22 | Give the source of funding and the role of the funders for the present study and, if applicable, for the original study on which the present article is based | - Declarations. Funding information: 1 | |

*Give information separately for exposed and unexposed groups.
