# Supplementary material for: Evaluation of the tuberculosis notification impact from an intermediary-facilitated public–private mix intervention in Viet Nam: A population-level quasi-experimental study
Source: PLoS Med. 2026 Sep 23;23(9):e1005144. doi: 10.1371/journal.pmed.1005144 (PMC13600524; doi:10.1371/journal.pmed.1005144)
Supplement: Supplementary file 3 [file pmed.1005144.s003.docx]

********************************************************************************

* S1 Code

*

* Population-level tuberculosis notification impact from an intermediary-

* facilitated public-private mix intervention in Viet Nam: a heterogeneity-

* robust difference-in-differences analysis of national surveillance data

*

* Corresponding author : Luan Nguyen Quang Vo (luan.vo@ki.se)

* Version / date	: 1.0

*

*-------------------------------------------------------------------------------

* PURPOSE

*-------------------------------------------------------------------------------

* This file reproduces the main analyses reported in the manuscript for sake of

* simplicity. It contains only the specifications underlying the results

* presented in the main text; sensitivity and robustness analyses reported in

* the supplementary appendix are not included, but can be derived from the

* provided code by altering model specifications accordingly.

*

* Three estimands are produced:

*	(1) Average treatment effect on the treated (ATT) and event-study dynamics for

*	quarterly province-level TB notifications, estimated with a Poisson

*	extended two-way fixed-effects (ETWFE) difference-in-differences estimator

*	(Wooldridge 2023), implemented in -jwdid- with never-treated provinces as

*	the comparison group (manuscript Table 2, Figure 3).

*	(2) Dose-response association between screening intensity and TB notifications,

*	estimated by Poisson pseudo-maximum likelihood with high-dimensional fixed

*	effects (-ppmlhdfe-), including a post-implementation persistence/fade-out

*	term (manuscript Table 2).

*	(3) National interrupted time-series (ITS) analysis of quarterly national TB

*	notifications, with model-based counterfactual predictions under zero

*	screening intensity (appendix Figure S1, Table S2.4).

*

*-------------------------------------------------------------------------------

* SOFTWARE AND DEPENDENCIES

*-------------------------------------------------------------------------------

* Stata 17 (StataCorp, College Station, TX, USA).

*

* Required user-written packages (SSC unless noted):

*	jwdid	Wooldridge (2023) ETWFE difference-in-differences estimator

*	ppmlhdfe Poisson pseudo-maximum likelihood with high-dimensional fixed effects

*	reghdfe	dependency of the above

*	ftools	dependency of the above

*

*	ssc install jwdid, replace

*	ssc install ppmlhdfe, replace

*	ssc install reghdfe, replace

*	ssc install ftools, replace

*

*-------------------------------------------------------------------------------

* INPUT DATA

*-------------------------------------------------------------------------------

* A balanced province-quarter panel covering Q1/2016-Q2/2025 (38 quarters), with

* one record per province per quarter. The file assumes the analysis dataset is

* already loaded in memory; insert the -use- statement below as appropriate.

*

* Expected variables:

*	Province_id	numeric province identifier (panel unit)

*	Province_EN	province name (string)

*	Region	geographic region (string)

*	Project	study arm / project assignment (string)

*	Year, Quarter	calendar year and quarter, used to construct the time index

*	tb_cases	all forms of TB notifications (primary outcome)

*	screened	persons receiving verbal symptom assessment

*	Population	province population (offset denominator)

*	treat_intensity verbal screens per 100,000 population per quarter

*	PPM, PPM5_type	PPM status indicators (descriptive; not used in the models)

*

* Data are derived from project-specific systems and the national TB surveillance

* system (VITIMES) and are not publicly available; see the data availability

* statement in the manuscript.

*

*-------------------------------------------------------------------------------

* OUTPUT FILES (written to the current working directory)

*-------------------------------------------------------------------------------

*	jwdid_event_full_treatment_period.xlsx	event-study coefficients

*	jwdid_event_full_treatment_period.png	event-study plot (Figure 3)

*	nat_poisson.ster	stored national ITS estimates

*	nat_its_predictions.dta	national ITS fitted/counterfactual series

*	National_ITS_estimates.xlsx	national ITS series, Excel format

*

*-------------------------------------------------------------------------------

* STRUCTURE

*-------------------------------------------------------------------------------

*	1. Data preparation and sample restriction

*	2. Construction of treatment, cohort and intensity variables

*	3. Descriptive statistics

*	4. ETWFE difference-in-differences (ATT and event study)

*	5. Dose-response model (PPMLHDFE)

*	6. National interrupted time-series analysis

*

*-------------------------------------------------------------------------------

* NOTES ON DESIGN DECISIONS

*-------------------------------------------------------------------------------

* Non-absorbing treatment. The intervention was deployed on province-specific

* start dates and ceased in all provinces at the end of Q4/2023. The -jwdid-

* estimator assumes absorbing treatment, so the estimation sample in Section 4 is

* truncated at Q4/2023. Within that window treatment is absorbing, and the

* post-intervention period cannot contaminate the ATT through persistence or

* fade-out effects. The dose-response model in Section 5 uses the full panel and

* addresses persistence explicitly through a post-implementation indicator.

*

* Comparison group. Never-treated provinces are assigned cohort = 0 and are used

* as the sole comparison group via the -never- option, so that not-yet-treated

* provinces are excluded as controls.

*

* Ten provinces with confounding active case-finding and PPM activities are

* excluded from both the intervention and comparison areas.

********************************************************************************

version 17.0

* use "<path to analysis dataset>", clear

********************************************************************************

* 1. DATA PREPARATION AND SAMPLE RESTRICTION

********************************************************************************

* Encode string identifiers used for sample restriction

* Note: -encode- assigns numeric codes in alphabetical order of the string values,

* so the codes referenced below are specific to this dataset. Verify with

* -label list- before re-running on an updated extract.

encode Province_EN, gen(prov)

encode Project, gen(proj)

* Time index (Stata quarterly date)

gen date = yq(Year, Quarter)

format %tq date

* Restrict analysis sample

drop if prov == 40	// central TB hospitals; not a province

drop if proj == 3	// 10 provinces with confounding case-finding/PPM activities

* Population offset

gen ln_population = ln(Population)

label var treat_intensity "Verbal screens per 100,000 pop"

********************************************************************************

* 2. TREATMENT, COHORT AND INTENSITY VARIABLES

*

* Treatment begins on province-specific dates and ends in Q4/2023 for all

* intervention provinces (non-absorbing treatment).

********************************************************************************

* Ever-treated indicator (15 intervention provinces)

gen ever_treated = 0

replace ever_treated = 1 if inlist(Province_id, ///

1, 2, 51, 33, 56, 27, 32, 44, 45, 60, 12, 18, 34, 36, 64)

* Treatment cohort = quarter of intervention launch

* Seven cohorts; never-treated provinces take cohort = 0 as required by -jwdid-

gen cohort = .

replace cohort = quarterly("2020q1", "YQ") if inlist(Province_id, 1, 2, 51)

replace cohort = quarterly("2021q4", "YQ") if inlist(Province_id, 33, 56)

replace cohort = quarterly("2022q1", "YQ") if Province_id == 45

replace cohort = quarterly("2022q2", "YQ") if inlist(Province_id, 27, 32)

replace cohort = quarterly("2022q3", "YQ") if inlist(Province_id, 44, 60)

replace cohort = quarterly("2023q2", "YQ") if inlist(Province_id, 12, 36)

replace cohort = quarterly("2023q3", "YQ") if inlist(Province_id, 18, 34, 64)

replace cohort = 0 if ever_treated == 0

format cohort %tq

* Treatment end date (common to all intervention provinces)

gen treatment_end = quarterly("2023q4", "YQ") if ever_treated == 1

format treatment_end %tq

* Active treatment indicator: 1 during the implementation window only,

* 0 both before and after

gen treated = 0

replace treated = 1 if (date >= cohort & date <= treatment_end) & cohort != 0

* Post-intervention period (after cessation of all activities)

gen post_intervention = 0

replace post_intervention = 1 if (date > treatment_end) & ever_treated == 1

* COVID-19 disruption period (peak service interruption in Viet Nam)

gen covid_period = 0

replace covid_period = 1 if date >= quarterly("2021q1", "YQ") ///

& date <= quarterly("2021q3", "YQ")

* COVID-adjusted screening intensity: intensity set to zero during peak disruption

gen treat_intensity_adj = treat_intensity

replace treat_intensity_adj = 0 if covid_period == 1

label variable ever_treated "Ever received intervention"

label variable cohort "Treatment start quarter (actual)"

label variable treatment_end "Treatment end quarter (Q4 2023)"

label variable treated "Currently treated (active period only)"

label variable post_intervention "Post-intervention period (after Q4 2023)"

label variable covid_period "COVID interruption period"

label variable treat_intensity_adj "Treatment intensity (COVID-adjusted)"

********************************************************************************

* 3. DESCRIPTIVE STATISTICS

********************************************************************************

* Mean screening intensity during active implementation

* (reported in the manuscript as 513.6 screens per province-quarter)

summ treat_intensity if treated == 1, detail

* Province-quarters with non-zero intervention intensity

* (reported in the manuscript as 112 treated province-quarters)

gen exposed = (treat_intensity > 0) & !missing(treat_intensity)

count if exposed == 1

local E = r(N)

display "Total exposed province-quarters = `E'"

* Aggregate quarterly notifications in intervention and control areas,

* over the full period and restricted to the pre-intervention period

preserve

collapse (sum) tb_cases screened Population, by(date ever_treated)

by ever_treated, sort: summ tb_cases, detail

by ever_treated, sort: ci mean tb_cases, poisson

by ever_treated, sort: ci mean tb_cases if date < quarterly("2020q1", "YQ"), poisson

restore

********************************************************************************

* 4. ETWFE DIFFERENCE-IN-DIFFERENCES (ATT AND EVENT STUDY)

*

* Main specification: full control group, estimation sample truncated at Q4/2023

* so that treatment is absorbing within the estimation window.

* Manuscript Table 2 and Figure 3.

********************************************************************************

tempfile maindata

save `maindata', replace

keep if date <= quarterly("2023q4", "YQ")

xtset Province_id date

jwdid tb_cases, ///

ivar(Province_id) ///

tvar(date) ///

gvar(cohort) ///

never ///

exposure(Population) ///

method(poisson) ///

hettype(event)

* Mean outcome among comparison provinces in the estimation sample

summarize tb_cases if e(sample) & proj == 1

*-------------------------------------------------------------------------------

* Parallel-trends diagnostics

*

* The -testparm- calls below reference the event-time coefficient names posted by

* -jwdid-, so they must be run before any -estat ..., estore()- call that replaces

* the estimation results in memory. Coefficient names can be listed with:

*	matrix list e(b)

*-------------------------------------------------------------------------------

* Full pre-intervention period

estat event, pretrend

* 8 pre-intervention quarters (t-8 to t-2)

testparm ///

23.__evnt__#c.__tr__ ///

24.__evnt__#c.__tr__ ///

25.__evnt__#c.__tr__ ///

26.__evnt__#c.__tr__ ///

27.__evnt__#c.__tr__ ///

28.__evnt__#c.__tr__ ///

29.__evnt__#c.__tr__

* 6 pre-intervention quarters (t-6 to t-2) - pre-specified primary window

testparm ///

25.__evnt__#c.__tr__ ///

26.__evnt__#c.__tr__ ///

27.__evnt__#c.__tr__ ///

28.__evnt__#c.__tr__ ///

29.__evnt__#c.__tr__

* 4 pre-intervention quarters (t-4 to t-2)

testparm ///

27.__evnt__#c.__tr__ ///

28.__evnt__#c.__tr__ ///

29.__evnt__#c.__tr__

*-------------------------------------------------------------------------------

* Aggregated ATT and event-study estimates

*-------------------------------------------------------------------------------

* Overall ATT on the count scale (manuscript Table 1)

estat simple, estore(att_full_treat)

* Event-time estimates (manuscript Figure 3)

estat event, estore(event_full_treat)

matrix event_results = r(table)'

*-------------------------------------------------------------------------------

* Export and plot event-study estimates

*

* Note: event_time is recovered from the row order of the coefficient matrix; the

* constant below maps the first row to its event time and must be checked against

* the -estat event- output if the estimation window changes.

*-------------------------------------------------------------------------------

preserve

clear

svmat event_results, names(col)

gen event_time	= _n - 31

gen scenario	= "Full Controls - Treatment Period"

gen sample_period = "2016Q1-2023Q4"

order scenario sample_period event_time b se z pvalue ll ul

export excel using "jwdid_event_full_treatment_period.xlsx", ///

firstrow(variables) replace

display "Exported: jwdid_event_full_treatment_period.xlsx"

* Confidence bounds are taken directly from r(table) (ll, ul)

gen pre_treatment = (event_time < 0 & event_time != -1)

gen post_treatment = (event_time >= 0)

twoway (rspike ul ll event_time if pre_treatment, lcolor("68 114 196") lwidth(medthick)) ///

(rspike ul ll event_time if post_treatment, lcolor("112 173 71") lwidth(medthick)) ///

(scatter b event_time if pre_treatment, mcolor("68 114 196") msize(medium) msymbol(circle)) ///

(scatter b event_time if post_treatment, mcolor("112 173 71") msize(medium) msymbol(circle)), ///

yline(0, lpattern(dash) lcolor(black)) ///

xline(-0.5, lpattern(solid) lcolor(gray)) ///

xlabel(, angle(45)) ///

ylabel(, angle(horizontal)) ///

xtitle("Quarters relative to treatment start") ///

ytitle("ATT (predicted counts)") ///

title("Event Study: Full Controls") ///

subtitle("Treatment Period Only (2016q1-2023q4)") ///

legend(off) ///

graphregion(color(white))

graph export "jwdid_event_full_treatment_period.png", replace

restore

* Restore the full panel (including the post-intervention period)

use `maindata', clear

********************************************************************************

* 5. DOSE-RESPONSE MODEL (PPMLHDFE)

*

* Full panel including the post-intervention period, so that persistence or

* fade-out of treatment effects after cessation can be tested directly.

* Manuscript Table 2.

*

* covid_period is retained for transparency but is collinear with the calendar-

* quarter fixed effects and is therefore dropped by the estimator, as noted in

* the manuscript table footnote.

********************************************************************************

ppmlhdfe tb_cases treat_intensity_adj covid_period post_intervention, ///

absorb(Province_id date) offset(ln_population) ///

vce(cluster Province_id) eform

********************************************************************************

* 6. NATIONAL INTERRUPTED TIME-SERIES ANALYSIS

*

* Outcome : tb_cases, aggregated to national quarterly counts

* Exposure : national screening rate per 100,000 population, constructed from the

*	unadjusted screening counts; peak COVID-19 disruption is handled by a

*	separate indicator rather than by zeroing intensity

* Offset	: log of national population

* Trend	: linear and quadratic secular terms, quarterly seasonality indicators

*

* Appendix Figure S1; Table S2.4.

********************************************************************************

preserve

* Aggregate province-quarters to national quarters

collapse (sum) tb_cases screened Population, by(date)

* National offset (regenerated: dropped by -collapse-)

gen double ln_population = ln(Population)

* Secular time trend

sort date

gen int	t = _n

gen double t2 = t^2

* Seasonality

gen byte qtr = quarter(dofq(date))

* COVID-19 disruption indicator

gen byte covid = inrange(date, quarterly("2021q1","YQ"), quarterly("2021q3","YQ"))

* National screening intensity per 100,000 population

gen double treat_intensity = (screened / Population) * 100000

label var treat_intensity "National screens per 100,000 pop"

* Poisson ITS model

poisson tb_cases c.treat_intensity c.t c.t2 i.qtr covid, ///

offset(ln_population) vce(robust)

estimates store nat_poisson

estimates save nat_poisson, replace

* Fitted values under observed screening intensity

predict double mu_hat, n

* Counterfactual fitted values under zero screening intensity

tempvar treat_store

gen double `treat_store' = treat_intensity

replace treat_intensity = 0

predict double mu_cf0, n

replace treat_intensity = `treat_store'

drop `treat_store'

* Observed, fitted and counterfactual series

sort date

twoway ///

(line tb_cases date) ///

(line mu_hat	date) ///

(line mu_cf0	date, lpattern(dash)), ///

legend(order(1 "Observed" 2 "Fitted" 3 "Counterfactual: intensity=0") cols(1)) ///

ytitle("TB notifications") xtitle("Quarter")

* Save the series underlying the figure

keep date tb_cases mu_hat mu_cf0 treat_intensity ln_population t t2 qtr covid

save nat_its_predictions.dta, replace

summ tb_cases mu_hat mu_cf0

export excel using "National_ITS_estimates.xlsx", ///

firstrow(variables) replace

* Results on the incidence rate ratio scale

estimates replay nat_poisson, irr

restore

********************************************************************************

* END OF FILE

********************************************************************************
