# Supplementary material for: Evaluation of the tuberculosis notification impact from an intermediary-facilitated public–private mix intervention in Viet Nam: A population-level quasi-experimental study
Source: PLoS Med. 2026 Sep 23;23(9):e1005144. doi: 10.1371/journal.pmed.1005144 (PMC13600524; doi:10.1371/journal.pmed.1005144)
Supplement: Supplementary file 4 [file pmed.1005144.s006.docx]

21 July 2026

Editorial Office of PLoS Medicine

Dear Dr. Alison Farrell,

Thank you very much for your consideration of the manuscript entitled “Population-level tuberculosis notification impact from an intermediary-facilitated public-private mix intervention in Viet Nam: a heterogeneity-robust difference-in-differences analysis of national surveillance data” (PMEDICINE-D-26-01810R1).

Further, we sincerely thank you and the peer reviewers for the valuable and constructive comments and believe that by addressing the points raised we have been able to improve the quality and readability of the manuscript. Below is a point-by-point response (*italics*). Partial revisions within full sentences were underlined. Wherever appropriate, we have provided line numbers of the edits. These numbers correspond to those in the revised version showing all tracked changes.

**Editor’s comments**

We ask you to provide additional context and rationale for the study in a final sentence of the Background section of the Abstract, add a clear explanation of the intervention in the Methods and Findings section of the Abstract (e.g. for a general reader), add further discussion of the potential implications of the results in the Discussion.

- *Thank you for the opportunity to revise the Abstract for better clarity and relevance. We fully concur with your assessment and have made substantial changes to the abstract in the hopes that this addresses current gaps and improves readability for the general audience.*
- *We have restructured the Background section to provide more context and rationale for the study. Specifically, we have added context around PPM and intermediary agencies and concluded the Background section with a summary of the rationale for the study in lines 44-50: “PPM strategies are promoted to improve TB notification, particularly in high-burden settings with fragmented care systems, and intermediary agencies are recognized as effective mechanisms to operationalize PPM by bridging NTP’s and out-of-network healthcare providers. However, population-level evidence is rare, as most evaluations have been limited to pilot studies, small geographic areas, or uncontrolled designs. This study documents the population-level impact of PPM through the use of heterogeneity-robust econometric methods.”*
- *To add a clear explanation of the intervention, we have removed lines 50-54 from the Background section and provided further detail in the Methods and Findings section as suggested. Furthermore, we have included the study design and other required parameters for observational study abstracts (e.g., study design, population and setting, etc).*
- *As such, the opening segment of the Methods and Findings section in lines 56-62 now reads: “This was a non-randomized, quasi-experimental evaluation of secondary data from a large-scale intermediary-facilitated PPM intervention conducted between 2020 and 2023 in 15 provinces of Viet Nam, covering 40.6 million people equivalent to 40% of the population. The intervention engaged 3,154 non-NTP providers in TB care, linking them to the NTP through systematic screening, referral, and reporting mechanisms. The model emphasized active case finding, provider incentives, patient support and integrated data systems to raise notifications as the primary outcome.”*
- *Similar to the above, we have added further discussion of the potential implications of the results in lines 80-87: “This study furnishes robust, policy-relevant evidence that intermediary-facilitated PPM implemented at scale in Viet Nam produced significant, dose-responsive increases in PPM and TB notifications at provincial and national levels. These findings promote intermediary agency models as an effective mechanism to operationalize PPM at scale and to generate public health benefits in closing TB treatment coverage gaps. In an era of constrained global health financing, leveraging existing provider networks through intermediary-facilitated PPM offers an affordable and scalable strategy to sustain momentum towards ending TB.*

**Reviewer 1 comments**

This is an excellent, timely, and policy-relevant manuscript that will provide an important contribution to the TB and public-private mix literature. The study addresses a major evidence gap: whether engagement of private and other non-NTP providers can produce gains in TB notification at a national level, in contrast to the multiple available pilot-studies/ data. The scale of the intervention is particularly impressive, covering approximately 40% of Viet Nam's population, with a large comparison area. The methodological approach is strong and appropriate for the non-randomized design. I found the concordance between total TB notification effects and PPM notification effects especially persuasive for attribution. I recommend publication. I have a few minor points, which serve more as suggestions for clarification.

*Thank you very much for your review and endorsement of the manuscript, and for the thoughtful suggestions on how to improve its quality. They were most welcome.*

1. First, because intervention provinces were purposively selected high-burden areas, the authors could further discuss the risk of residual confounding from political, administrative, or programmatic factors that may have influenced both selection and notification trends.
   - *Thank you very much for this sensible suggestion. We have added the following description to the limitations in the discussion in lines 509-514: “For example, the project specifically targeted provinces with progressive health authorities keen to reduce their TB burden, who leveraged their political influence to promote non-NTP provider engagement under Viet Nam’s unitary political environment. Moreover, urban areas tend to offer better healthcare infrastructure, which attracts greater health-seeking compared to rural and remote provinces. These structural differences likely led to an overestimation of the intervention’s treatment effect and notification trends.”*
   - *Given a thematic similarity, we have also moved the final limitation with slight wording changes to this section in lines 515-518: “Similarly, while the intermediate analysis using PPM notifications strengthens the causal interpretation, it also means that scaling the model to settings with structurally different private-sector notification pathways may not produce equivalent effects on total TB notifications.”*
2. Second, the authors could better distinguish the relative contributions of increased screening/referral versus private treatment reporting.
   - *This was also a very astute observation, which we have tried to include in the results and discussion.*
   - *In the results, we have modified the text in lines 333-334 as follows: “Under Strategy 2 (Table S11 of S1 Appendix), 134 providers reported 12,839 private TB treatments, of whom 95.3% (12,231/12,839) passed the quality control process for official notification by the NTP, representing 30.0% (12,231/40,733) of total treatment initiations across both strategies. About 73.3% (9,414/12,231) of privately treated persons were reported to have successfully completed treatment.”*
   - *In the discussion, we have added the following text in lines 422-425: “Remarkably, less than 1% of providers in the master registry contributed about three-tenths of the total TB notification yield, suggesting that a refined intervention and targeted provider engagement could generate a large impact with modest efforts through the intermediary.”*
3. Finally, the cost analysis would be strengthened by discussing implications for routine implementation, including health-system and patient costs not captured by the funder perspective. The strategy of intermediary agency as a crucial phase for TB interventions within public-private mix systems is clear. But a brief reflection in the discussion on the sustainability of this strategy versus other intermediary systems solutions (such as health insurance) will add to health systems strengthening lens. Routine program scale-up processes, costs saved to both programs as well as to patients, could be mentioned as areas for further research to strengthen the current evidence of engaging the private sector.
   - *To meet this request, we have added the following text to lines 480-486: “However, the scale-up faced early implementation barriers. First, insurance coverage for instrumental funder-related costs such as the lack of provider incentives and patient cash transfers included in statutory reimbursement packages. Second, provincial TB programs lacked the necessary human resources to sustain the model’s intensive provider engagement model. Critically, this effort was disrupted by the country’s 2025 administrative reform. Thus, critical evidence gaps on the model’s replicability, scalability and sustainability under routine program operations remain.”*
   - *We have also added the suggestion on further research topics to lines 475-477: “Nevertheless, routine program scale-up processes as well as costs saved to programs and patients through a full health economic analysis comprise areas for further research to strengthen the current evidence of engaging the private sector.”*
4. Overall, this is a high-quality manuscript with substantial relevance for current national TB planning (including Global Fund GC8), particularly in a period of constrained TB financing. It provides rare and much-needed population-level evidence that intermediary-supported PPM strategies can make a concrete and cost-effective contribution to TB notification. The discussion is a great reflection on the findings while also highlighting outstanding gaps in private sector engagement for contact tracing and treatment support.
   - *Once again, thank you so much for the positive reception and endorsement of the work and manuscript.*

**Reviewer 2 comments**

This is an interesting manuscript on a topic of substantial public health relevance. It estimates the real-world impact of an intervention designed to strengthen links between private providers and the National Tuberculosis (TB) Program of Vietnam, with respect to increases in TB notifications. It also estimates the marginal cost per additional notification. It suggests that the intervention had a measurable impact on TB notifications to national public health authorities, by supporting suitable screening, diagnostic testing and reporting. This is an important message. That said, I have several comments about the manuscript in its current form.

*Thank you very much for your thorough review of the manuscript. We are also grateful for the thought-provoking comments and hope that we have addressed them adequately.*

1. Throughout the manuscript (methods, results, discussion) there is considerable emphasis on the statistical analyses used to detect/triangulate an increase in TB case notifications in provinces where the intervention was implemented, relative to what would otherwise have been expected over the same time period—and to distinguish this from increases potentially due to other factors. While methodologic rigor is of course essential, I perceive that some of this leads to the main text reading in a way that will be overly technical for many readers. Some of this detail could be further relegated to the appendix, to make space in the main text for necessary description and reflection around more public health- and program-oriented issues. The technical methods and detailed results should support policy, health services and public health; they should not become the main message, in my view.
   - *Your concern about the manuscript’s relevance for readers focused on policy, health service and public health is well received. As such, we have adjusted the methods and discussion for a greater focus on the aforementioned real-world issues.*
   - *We expanded the intervention description as outlined in the next comment as well as with content from section S1.2 of S1 Appendix in lines 221-223: “These activities were designed to result in improved detection and diagnostic quality; stronger engagement and active participation; higher quality of treatment and care; and greater transparency into private sector practices and notifications.”*
   - *Concordant with reviewer #1, we also expanded the discussion on barriers to scale-up and sustainability (comment #3) and replaced the limitation related to the Difference-in-Differences framework, and specifically on the Stable Unit Treatment Value Assumption, with a design limitation related to the purposive selection of the intervention provinces (reviewer #1, comment #1).*
   - *We initially opted to provide substantive information on the statistical methods to allow scrutiny of the claim that the intervention generated a population-level impact. We still believe in the critical importance of assuaging any methodological doubt, and as such kindly ask to accept our request to retain the remaining analytical detail in the manuscript.*
2. A clear example is the lack of clarity around the intervention itself in the main manuscript—most notably who the target population consisted of, and what screening algorithm was used. It is stated, without further explanation, that "health-seeking individuals" who presented to participating providers were eligible for assessment. In reading the appendix several times, I gleaned that this assessment involved the standard 4-symptom screen, plus (as far as I can tell) chest X-rays (CXRs) for all those seen, regardless of the presence or absence of symptoms. But this is never clearly stated. If readers wish to judge the generalizability or scalability of the intervention reported here, this type of information is essential. It's also essential that the authors clearly spell out how these screening and assessment practices differed (or not) from those in the "control" settings, and from those in public sector facilities in both intervention and control provinces.
   - *We acknowledge your concern and have expanded the description of intervention design substantially in the hope that this improves the manuscript’s balance between operational and analytical focus. Specifically, we have replaced the previous 2-sentence description of the provider activities with the following text in lines 206-220: “Under Strategy 1, persons seeking care at participating healthcare providers were clinically assessed using the WHO 4-symptom screen and referred for CXR screening on-site or at another radiography site. Persons with TB-related abnormalities on CXR received confirmatory molecular testing on Xpert MTB/RIF (Ultra) at an NTP-supported laboratory; those diagnosed with TB were linked to the NTP for any necessary reflex diagnostics and treatment initiation. Under Strategy 2, providers submitted quarterly demographic and clinical data of treatment initiations and outcomes to the intermediary agency. Providers collected sputum from persons with bacteriologically-unconfirmed TB for molecular testing, if within two weeks of treatment initiation, and referred persons with rifampicin-resistant TB to the NTP. Agency staff managed quality control and entry of validated records into the NTP’s digital TB register, collected and transported samples for testing, communicated Xpert result to providers and supported treatment follow up. The latter included tracing treatment interruptions and facilitating transfer to the NTP, if cost was the reason for discontinuation. The intervention provided financial support to recommended disadvantaged patients and paid stipends to providers for each reported treatment initiation and outcome.”*
3. Similarly, there is little information about the process or factors that ultimately led some providers to participate, and others not to.  Over half of those eligible were excluded as they were "unreached/not approached" [figure S4].  What does this mean?  Why did only 26% of providers with a signed contract send at least one person for a CXR?  And do the authors have any information, beyond place of practice, that allows them (and readers) to look for differences between eligible providers who did vs. did not participate, including those reached who declined participation?  This relates directly to potential biases and to generalizability—topics that are not really addressed as potential limitations or concerns in the discussion section.  Instead, the only limitations specifically addressed are of a technical/statistical nature.
   - *Given the large volume of providers and scale of the intervention, details on the gaps in the provider engagement cascade beyond the information provided in Figure S4 were unfortunately unavailable. As per your suggestion, we have added text to highlight this limitation in lines 520-527, which replaced the prior second analytical limitation related to the ETWFE results: “…the provider engagement cascade exhibited steeps drops between the number of providers in the master registries and those actively participating and positively contributing to TB notifications. Similarly, we observed substantial deviations in the distribution of provider types in the master registries, signing participation contracts, actively participating and contributing to case detections by typology. However, given the large volume of providers and scale of the intervention, provider engagement barriers and root causes were unavailable and represent key areas for future implementation research.”*
4. Continuing with the same theme, what do these results tell us about potential scalability? In other words, the authors make a convincing argument that the intervention had a measurable impact on TB notifications to the national program. But this impact appears relatively modest, in absolute terms, as does the proportion of participating private providers. Based on their data and experience, does this intervention appear scalable, to reach more providers, and more people with possible TB? How likely is a similar intervention to work in other settings? Along those lines, it would be helpful to readers to better describe what exactly the intervention consisted of, and who did what; the description on page 9 is very broad: "The intermediary identified, contracted, and supported private providers through training, supervision, and access to diagnostic services, while facilitating systematic screening, referral, and reporting of presumptive TB cases."
   - *The critique about the insufficient intervention detail is well-noted and has been expanded substantially as part of the revision associated with comment #2. Similarly, the limitation of the suboptimal provider participation has been acknowledged and addressed as part of comment #3.*
   - *We are similarly keen to further understand the posed implementation science-related questions, particularly on context-dependent scalability. Based on this comment and similar feedback from reviewer #1, we have added additional detail around barriers to scalability and replicability in the context of the NTP’s effort to institutionalize the model in lines 480-484.*
5. The cost analysis requires some revision and reframing. The conceptual difference/differing definitions between the provincial and national estimates for marginal cost per additional notification are not explained, making the substantial difference between the provincial estimate ($166) and the national estimate ($458) very difficult to understand. In my own view, the additional cost per additional notification should be the same, if all the input costs have been suitably assigned and prorated. In addition, the perspective of the cost analysis is somewhat problematic. It focuses exclusively on the cost of services and training provided by or purchased by the intermediary. This might be relevant to third-party funders, but the intervention brings other costs that are directly relevant to the health system, and that would need to be considered by local/national decision makers contemplating adoption of such an intervention. Referrals by private providers to the public system for CXRs and molecular diagnostics, for example, are a desired outcome but they imply additional costs to the public system which are not captured by the current cost analysis, and again are directly relevant to questions of implementation and scalability. These additional costs to the health care system should be estimated, or at minimum discussed. And the authors should consider that the national program itself, rather than third party donor agencies, may in future take responsibility for costs and implementation of such an intervention.
   - *Thank you for the opportunity to clarify the mechanics and rationale around this analysis.*
   - *With regards to the mechanics, we have added text to the methods to arrive at the provincial and national marginal costs estimates in lines 295-298: “…calculated marginal costs per additional notification at provincial and national levels. At the provincial level, total project costs were divided by the product of the ATT times 112 province-quarters of intervention. At the national level, total project costs were divided by the product of additional TB notifications per year times four years of intervention.”*
   - *With regards to the rationale, we agree that a full health economic analysis assuming a societal perspective as described would be most preferable, and such an analysis is planned as a future study of the intervention, given that it is out of scope for this impact evaluation.*
   - *As such, the micro-costing analysis included in this manuscript does not seek to assess the health economics of the intervention in the national context but rather aims to offer a benchmark with similar interventions in the global context. This was done by assuming the funder-perspective and comparing it to the cost benchmarks provided by Rahman et al.[1]*
   - *Nevertheless, a sentence to suggest that the intervention warrants a full health economic analysis was added in lines 475-477: “Nevertheless, routine program scale-up processes as well as costs saved to programs and patients through a full health economic analysis comprise areas for further research to strengthen the current evidence of engaging the private sector.”*
6. The discussion section seems longer than necessary and, in my view, should focus more clearly on implications and limitations of this work, beyond technical issues related to the statistical analysis. As mentioned previously, the authors need to explicitly consider potential bias in the cascade of participating providers (and those who referred patients for CXRs), as well as questions of scalability, generalizability, and potential budget impact.
   - *In agreement with this observation and prior comments, two of the three analytical limitations have been replaced by operational limitations related to the purposive selection of the intervention provinces and the lack of detail on the root causes for the drops in the engagement cascade.*
7. Page 6, line 105: "legal escrow" seems an unusual term. Consider rephrasing.
   - *The term has been rephrased as “an intermediary.”*

**Reviewer 3 comments**

The paper uses a difference in differences approach to assess the casual effect of PPM intervention on TB notifications using data from Viet Nam. It also applies an interrupted time series approach as a secondary analysis. Overall, the statistical methods are appropriate, and are generally well reported and presented. There is a good discussion of the assumptions and acknowledgement of the study's limitations. I have the following comments around the models used, and how these reflect the intervention made in practice.

*Per the comments from the other reviewers, some of the statistical content and particularly the second and third limitation of the modeling were replaced with operational limitations and moved to Section S1.4 in S1 Appendix instead.*

1. Line 162 notes dynamic changes in intervention intensity. Could these dynamic changes be elaborated? How was this incorporated into the modelling? It might be useful to provide the difference in differences modelling equation, perhaps part of Section S1.4, in order to illustrate whether, and how, this changing intensity was accounted for.
   - *Thank you for this comment as we agree that the definition of intensity (“Intensity was modeled as a continuous exposure of the population-adjusted rate of persons undergoing verbal symptom assessment per 100,000 population per quarter”), which does not appear until the statistical analysis section, should be included here for improved readability. As such, we have moved this definition up to lines 184-185.*
   - *An equation of the ETWFE model was included in Section S1.4.[2,3] The model is written as follows:*

$$log(\frac{\mu_{it}}{{Pop}_{it}})=\xi_{i}+\xi_{t}+\sum_{g} \sum_{e\neq-1} \vartheta_{ge}D_{ig}E_{it}^{e}$$

*where*

- *Y_it_ = number of TB notifications in province i during quarter t;*
- *μ_it_ = E(Y_it_) expected number of TB notifications;*
- *Pop_it_ = province population (exposure offset term);*
- *ξ_i_ = province fixed effects accounting for time-invariant provincial characteristics;*
- *ξ_t_ = quarter fixed effects capturing national temporal shocks common to all provinces (including secular trends and COVID-19-related influences);*
- *exp(θ_ge_) = event-time-specific average treatment effect on the treated (ATT);*
- *D_ig_ = indicator (dummy) that province i belongs to treatment cohort g;*
- $E_{it}^{e}$ *= indicator for event time e(quarters relative to the reference period of the intervention launch at e=-1) with e = t – G_i_, where G_i_ denotes the intervention (cohort) quarter for province i.*
  - *However, the ETWFE framework specified event-based heterogeneity but not intensity. Instead, intensity was included in the Poisson Pseudo Maximum Likelihood (PPMLHDFE) models that estimated the dose-response effect, for which an equation was included in Section S1.6 as well.[4] The model is written as follows:*

$$\log\left( \frac{\mu_{it}}{{Pop}_{it}} \right)=\xi_{i}+\xi_{t}+\beta_{1}I_{it}+\beta_{2}C_{t}+\beta_{3}P_{t}$$

*where*

- *μ_it_ = E(Y_it_) expected number of TB notifications, where Y_it_ = number of notified TB cases in province i during quarter t and Y_it_ ~ Poisson(μ_it_);*
- *Pop_it_ = province population (exposure offset term);*
- *ξ_i_ = province fixed effects accounting for time-invariant provincial characteristics;*
- *ξ_t_ = quarter fixed effects capturing national temporal shocks common to all provinces;*
- *I_it_ = intervention intensity (verbal assessments);*
- *C_t_ = COVID-19 period indicator (function of time only);*
- *P_t_ = post-intervention indicator (function of time only).*

2. Similarly, for the interrupted time series model, how was the intervention period modelled - was the intervention included as a single time point, or was the dynamic intervention period accounted for? It might again be useful to provide the modelling equation in Section S1.7 to show how this was implemented.
   - *We thank the reviewer for this suggestion. The intervention was not modelled as a single interruption or binary step function. Instead, because implementation intensity varied substantially over time, the interrupted time series (ITS) incorporated a continuous quarterly measure of national screening intensity (screenings per 100,000 population) as a time-varying exposure. This approach accounted for the dynamic nature of the intervention throughout implementation rather than assuming an immediate and constant level change after a single intervention launch date.*
   - *We have thus added the following explanation to S1 Appendix, Section S1.7: “Unlike conventional segmented ITS analyses that model intervention exposure using binary level and slope-change indicators, the present analysis represented implementation as a continuous time-varying intervention intensity. Quarterly screening volume (screenings per 100,000 population) served as a proxy for implementation intensity, allowing the model to capture the gradual scale-up, varying intensity, and subsequent decline of program activities over time. This specification was considered more appropriate than a binary intervention indicator because program implementation was neither instantaneous nor constant across the study period.”*
   - *An equation of the ITS model was included in Section S1.7 as requested.[5–7] The model is written as follows:*

$$\log\left( \frac{\mu_{t}}{{Pop}_{it}} \right)=\beta_{0}+\beta_{1}I_{t}+\beta_{2}t+\beta_{3}t^{2}+\sum_{q=2}^{4} \delta_{q}Q_{qt}+\gamma C_{t}$$

*where*

- *Y_it_ = number of TB notifications nationally during quarter t;*
- *μ_t_ = E(Y_t_) expected number of national TB notifications, where Y_t_ = number of national TB notifications during quarter t and Y_t_ ~ Poisson(μ_t_);*
- *Pop_t_ = national population (offset);*
- *I_t_ = national screening intensity (screenings per 100,000 population);*
- *t, t^2^ = continuous & quadratic quarterly time trend;*
- *Q_qt_ = quarter-of-year indicators (Q2–Q4; Q1 reference);*
- *C_t_ = COVID-19 disruption indicator (2021/Q1–2021/Q3);*
- *β_0_ = baseline log notification rate;*
- *β_1_ = effect of screening intensity;*
- *β_2_, β_3_ = secular trends;*
- *δ_q_ = seasonal effects;*
- *γ = COVID-19 effect.*

3. Some more detail on the selection of provinces for intervention would be beneficial. In Line 145 it is mentioned they were selected as high-burden settings, but this isn't explained. This would help to give context to the intervention being analysed, and for evaluation of the potential for residual confounding.
   - *Other reviewers have provided similar feedback. Hence, additional detail on the type of residual confounding and consequences on the outcomes from the purposive selection was included in lines 508-513.*

**Technical requests**

1. Please send a completed 'Competing Interests' statement, including any COIs declared by your co-authors. If you have no competing interests to declare, please state "The authors have declared that no competing interests exist". Otherwise please declare all competing interests beginning with the statement "I have read the journal's policy and the authors of this manuscript have the following competing interests:"
   - *We have included a competing interest statement in the manuscript and have added the text above to that statement (also in lines 583-584 and 588 of the manuscript). All co-authors have indicated their COI through the submission platform.*
2. Please provide separate figure files in .tif or .eps format. For more information about figure files please see our guidelines.
   - *We have uploaded TIF-format images that were processed using the NAAS tool for all three figures.*
3. Please note that the contact for accessing your data cannot be an author on the manuscript. Please therefore replace the current contact information with a third party contact, such as an institutional data access committee.
   - *We have updated the contact information in the revised manuscript for the data custodians, i.e., the National TB Control Programme, and removed all mentions of the corresponding author as contact for accessing the data in lines 605-607.*
4. Some material included in your submission may be copyrighted. According to PLOS’s copyright policy, authors who use figures or other material (e.g., graphics, clipart, maps) from another author or copyright holder must demonstrate or obtain permission to publish this material under the Creative Commons Attribution 4.0 International (CC BY 4.0) License used by PLOS journals. Please closely review the details of PLOS’s copyright requirements here: PLOS Licenses and Copyright. If you need to request permissions from a copyright holder, you may use PLOS's Copyright Content Permission form.

Potential Copyright Issues:

i) Figure [1]: please (a) provide a direct link to the base layer of the map (i.e., the country or region border shape) and ensure this is also included in the figure legend; and (b) provide a link to the terms of use / license information for the base layer image or shapefile. We cannot publish proprietary or copyrighted maps (e.g. Google Maps, Mapquest) and the terms of use for your map base layer must be compatible with our CC BY 4.0 license.

- *a) The map was created using QGIS software, with source shape files coming from GADM. The relevant link is here: https://gadm.org/download_country.html*

*Figure 1 was uploaded with the link to the shapefile included in the figure legend.*

- *b) The link to the license information for the shapefile is here:*

*https://gadm.org/license.html*

*It explicitly says the following: "The data are freely available for academic use and other non-commercial use. Redistribution or commercial use is not allowed without prior permission. Using the data to create maps for publishing of academic research articles is allowed. Thus you can use the maps you made with GADM data for figures in articles published by PLoS, Springer Nature, Elsevier, MDPI, etc. You are allowed (but not required) to publish these articles (and the maps they contain) under an open license such as CC-BY as is the case with PLoS journals and may be the case with other open access articles."*

ii) [Figure 2]: Please confirm whether you drew the images / clip-art within the figure panels by hand. If you did not draw the images, please provide (a) a link to the source of the images or icons and their license / terms of use; or (b) written permission from the copyright holder to publish the images or icons under our CC BY 4.0 license.

- *A revised version of Figure 2 has been uploaded, in which all clip-art has been removed.*

**General editorial requests**

Note: not all will apply to your paper, but please check each item carefully.

*We have reviewed the editorial requests. While we have included all requests as suggested in your communication, those not applicable are preceded with an “N/A” for ease of tracking. All comments for specific types of observational studies as well as those for non-applicable study types such as modeling and health economics / cost-effectiveness studies were omitted from this letter. In addition, we have made minor editorial changes for greater clarity (line 317) and more person-centered language (lines 198; 351).*

1. We ask every co-author listed on the manuscript to fill in a contributing author statement, making sure to declare all competing interests.
   - *N/A – this was done as part of the original submission.*
2. Please upload any figures associated with your paper as individual TIF or EPS files with 300dpi resolution at resubmission.
   - *The figures were processed through the PLOS's NAAS tool and reuploaded.*
3. For data residing with a third party, authors are required to provide instructions with contact information (web or email address) for obtaining the data. Please note that a study author cannot be the contact person for the data.
   - *As stated in the Technical requests section above, the data availability statement in the manuscript was updated in lines 605-607 to include the contact information for the National TB Program for submission of data requests.*
4. We expect all researchers with submissions to PLOS in which author-generated code underpins the findings in the manuscript to make all author-generated code available without restrictions upon publication of the work. In cases where code is central to the manuscript, we require the code to be made available as a condition of publication. Authors are responsible for ensuring that the code is reusable and well documented. Please make any custom code available, either as part of your data deposition or as a supplementary file. Please add a sentence to your data availability statement regarding any code used in the study, e.g. "The code used in the analysis is available from Github [URL] and archived in Zenodo [DOI link]" Please review our guidelines at https://journals.plos.org/plosmedicine/s/materials-software-and-code-sharing and ensure that your code is shared in a way that follows best practice and facilitates reproducibility and reuse. Because Github depositions can be readily changed or deleted, we encourage you to make a permanent DOI'd copy (e.g. in Zenodo) and provide the URL.
   - *The Stata code for the main analyses has been uploaded as a supplementary file.*
   - *A corresponding statement was added to the Data availability section in line 612: “The code used in the analysis is included in S1 Code.”*
5. Please separate the funding statement from the competing interests statement.
   - *N/A – this was done as part of the original submission.*
6. Please provide a URL for each funder.
   - *The funder URL’s were added as requested.*
7. Please include a disclosure pertaining to any use of AI in assembling the manuscript or analyzing or generating the data.
   - *An AI Disclosure statement was added to the Acknowledgements of the manuscript’s declarations section (lines 622-626): “Anthropic's Claude (version 4; Opus) and OpenAI’s ChatGPT (GPT-4) were used to improve the readability and language of the manuscript and to assist with debugging and annotating analysis code, which was verified by the authors. The tool was not used to design the study, analyze or interpret data, or draw scientific conclusions. We reviewed all output and take full responsibility for the content of this manuscript.”*
8. Abstract: Please structure your abstract using the PLOS Medicine headings (Background, Methods and Findings, Conclusions). Please combine the Methods and Findings sections into one section.
   - *We have adjusted the first abstract section to say Background instead of Introduction.*
9. At this stage, we ask that you include a short, non-technical Author Summary of your research to make findings accessible to a wide audience that includes both scientists and non-scientists. The Author Summary should immediately follow the Abstract in your revised manuscript. This text is subject to editorial change and should be distinct from the scientific abstract. Ideally each sub-heading should contain 2-3 single sentence, concise bullet points containing the most salient points from your study.
   - *N/A – this was done as part of the original submission.*
10. Please add bullet points to your Author Summary.
    - *The author summary has been edited to consist of single-sentence bullet points.*
11. In the final bullet point of 'What Do These Findings Mean?', please include the main limitations of the study in non-technical language. Authors should also consider how the interpretation of the study’s findings may be affected by the study limitations.
    - *A bullet on the main limitation and its potential impact on the study findings was included.*
12. Please express the main results with 95% CIs as well as p values. When reporting p values please report as p<0.001 and where higher as the exact p value p=0.002, for example. Throughout, suggest reporting statistical information as follows to improve clarity for the reader "22% (95% CI [13%,28%]; p</=)". Please be sure to define all numerical values at first use.
    - *We have reviewed all mentions of 95% CI and added p-values (lines 396-397) or harmonized their formatting (line 266). One exception is line 376, where the p-value is the same as in the immediately preceding sentence (line 374) to avoid redundancy.*
13. Please include page numbers and line numbers in the manuscript file. Use continuous line numbers (do not restart the numbering on each page).
    - *N/A – this was done as part of the original submission.*
14. Please cite the reference numbers in square brackets. Citations should precede punctuation.
    - *N/A – this was done as part of the original submission.*
15. Please provide titles and legends for all figures and tables (including those in Supporting Information files).
    - *N/A – this was done as part of the original submission.*
16. Please define all abbreviations used in each figure/table (including those in Supporting Information files).
    - *N/A – this was done as part of the original submission.*
17. Please consider avoiding the use of red and green in order to make your figure more accessible to those with color blindness.
    - *N/A – this was done as part of the original submission.*
18. Please ensure that the figures do not involve copyrighted material (e.g. Fig.1, 2).
    - *This was addressed in the Technical requests section above.*
19. Please note that supplementary material will be posted as supplied by the authors. Therefore, please amend it according to the relevant comments outlined here.
    - *This was addressed in the Reviewer feedback sections above.*
20. Please cite your Supporting Information as outlined here.
    - *N/A – this was done as part of the original submission.*
21. PLOS uses the numbered citation (citation-sequence) method and first six authors, et al.
    - *N/A – this was done as part of the original submission.*
22. Please ensure that journal name abbreviations match those found in the National Center for Biotechnology Information (NCBI) databases (http://www.ncbi.nlm.nih.gov/nlmcatalog/journals), and are appropriately formatted and capitalised.
    - *N/A – No journal name abbreviations were used in this manuscript.*
23. Where website addresses are cited, please include the complete URL and specify the date of access (e.g. [accessed: 12/06/2023]).
    - *N/A – No website addresses were cited in this manuscript.*
24. Abstract: Please include the study design, population and setting, number of participants, years during which the study took place (enrollment and follow up), length of follow up, and main outcome measures.
    - *This was addressed in the Editor’s comments section above.*
25. Please ensure that the study is reported according to the STROBE (or appropriate STOBE extension) guideline (available from: https://www.equator-network.org/reporting-guidelines/strobe) and include the completed STROBE (or STROBE extension) checklist as Supporting Information. Please add the following statement, or similar, to the Methods: "This study is reported as per the Strengthening the Reporting of Observational Studies in Epidemiology (STROBE) guideline (S1 Checklist)." When completing the checklist, please use section and paragraph numbers, rather than page numbers.
    - *N/A – this was done as part of the original submission.*
26. For all observational studies, in the manuscript text, please indicate: (1) the specific hypotheses you intended to test, (2) the analytical methods by which you planned to test them, (3) the analyses you actually performed, and (4) when reported analyses differ from those that were planned, transparent explanations for differences that affect the reliability of the study's results. If a reported analysis was performed based on an interesting but unanticipated pattern in the data, please be clear that the analysis was data driven.
    - *N/A – this was done as part of the original submission.*
27. Please state in the Methods section whether the study had a prospective protocol or analysis plan.
    - *We did not develop a prospective analysis plan for the study. A corresponding comment was added to the Statistical Analysis section (lines 247-248).*

Thank you once again for your review and assessment of our manuscript and please do not hesitate to contact us if there are any additional requests.

Sincerely and on behalf of the study team,

Luan Vo

Friends for International TB Relief

[luan.vo@tbhelp.org](mailto:luan.vo@tbhelp.org)

**References**

1.	Rahman MdT, Wells WA, Ramis O, Kamineni V V, Bakker MI, Matiku S, et al. Engaging private providers to enhance tuberculosis detection and notification: evidence from TB REACH-Supported projects. BMC Public Health. 2025;25: 665. doi:10.1186/s12889-025-21806-4

2.	Wooldridge JM. Simple approaches to nonlinear difference-in-differences with panel data. Econometrics Journal. 2023;26: C31–C66. doi:10.1093/ectj/utad016

3.	Rios-Avila F. jwdid: Flexible Estimation of staggered DID designs. 2025.

4.	Correia S, Guimarães P, Zylkin TZ. Fast Poisson estimation with high-dimensional fixed effects. Stata Journal. 2020;20: 95–115. doi:10.1177/1536867X20909691

5.	Bernal JL, Cummins S, Gasparrini A. Interrupted time series regression for the evaluation of public health interventions : a tutorial. International Journal of Epidemiology. 2017;46: 348–355. doi:10.1093/ije/dyw098

6.	Wagner AK, Soumerai SB, Zhang F, Ross-Degnan D. Segmented regression analysis of interrupted time series studies in medication use research. J Clin Pharm Ther. 2002;27: 299–309. Available: http://www.ncbi.nlm.nih.gov/pubmed/12174032

7.	Bernal JL, Cummins S, Gasparrini A. The use of controls in interrupted time series studies of public health interventions. Int J Epidemiol. 2018;47: 2082–2093. doi:10.1093/ije/dyy135
