# Supplementary material for: Evaluation of the tuberculosis notification impact from an intermediary-facilitated public–private mix intervention in Viet Nam: A population-level quasi-experimental study
Source: PLoS Med. 2026 Sep 23;23(9):e1005144. doi: 10.1371/journal.pmed.1005144 (PMC13600524; doi:10.1371/journal.pmed.1005144)
Supplement: Supplementary file 5 [file pmed.1005144.s007.docx]

25 August 2026

Editorial Office of PLoS Medicine

Dear Dr. Alison Farrell,

Thank you very much for your continued support of the manuscript entitled “Population-level tuberculosis notification impact from an intermediary-facilitated public-private mix intervention in Viet Nam: a heterogeneity-robust difference-in-differences analysis of national surveillance data” (PMEDICINE-D-26-01810R1).

Further, we sincerely thank peer reviewers 1 and 3 for their positive response to the changes, and we thank the editors and the peer reviewers for the additional constructive feedback. We hope that by addressing these additional points the manuscript has made further progress towards publication. As before, below is a point-by-point response (*italics*). Partial revisions within full sentences were underlined. Wherever appropriate, we have provided line numbers of the edits. These numbers correspond to those in the revised version showing all tracked changes.

**Editor’s requests**

1. Line 46: revise ‘NTP’s’ to ‘NTPs’
   - *The change has been made as requested.*
2. Line 49-50: please revise “through the use of heterogeneity-robust econometric methods.” to be accessible to a general reader. Why is this aspect important in the Background of the Abstract?
   - *We have adjusted the sentence to minimize technical jargon and describe the importance and rationale for the analytical choice to say (line 50-53): “This study documents the population-level association of a PPM intervention with TB notifications through the use of unbiased analytical methods to account for heterogeneity from the intervention’s staggered deployment and dynamic intensity.”*
3. Line 51: Revise to present tense, i.e. ‘We performed a non-randomized, quasi-…”
   - *We have adjusted lines 54-55 to use the present tense to state: “We present a non-randomized, quasi-experimental evaluation of a large-scale intermediary-facilitated PPM intervention…”*
4. Line 58: Please clarify the time frame in relation to the intervention.
   - *We have indicated the time frame of the intervention in lines 55-56 (“…a large-scale intermediary-facilitated PPM intervention conducted between 2020 and 2023 in 15 provinces of Viet Nam…) and believe that moving this time frame to line 58 or stating it again may be perceived as less coherent or redundant, respectively, so that we would like to suggest to keep the current location and mention of the intervention time frame.*
5. Line 76: please use associative rather than causal language.
   - *We have adjusted the sentence in line 81 to say: “This study furnishes robust, policy-relevant evidence that intermediary-facilitated PPM implemented at scale in Viet Nam was associated with significant, dose-responsive increases in PPM and TB notifications at provincial and national levels.”*
6. Lines 104-105: Please revise phrasing of the last sentence in the Author summary to be accessible to a general reader.
   - *We have adjusted the final bullet in lines 108-112 to state: “The main limitation was the non-randomized selection of the intervention provinces, which may have overstated the number of additional notifications from the intervention. This was mitigated through conservative selection of the control area and use of multiple methods and outcome variables to triangulate the results.”*
7. Line 112: please clarify what is meant by ‘provider-induced’
   - *The intention was to state that half of all unreached persons with TB were caused by the lack of reporting by healthcare providers. Lines 118-120 have been revised to say: “Unreached persons with TB can be bifurcated into undiagnosed and unreported groups, with the latter consisting of privately treated by not reported persons with TB*, highlighting the critical role of unengaged healthcare providers.*”*
8. Please confirm that your title complies with PLOS Medicine's style. Your title must be nondeclarative and not a question. It should begin with main concept if possible. "Effect of" should be used only if causality can be inferred, i.e., for an RCT. Please place the study design ("A randomized controlled trial," "A retrospective study," "A modelling study," etc.) in the subtitle (ie, after a colon).
   - *We have revised the title to state the study design explicitly as: "Evaluation of the tuberculosis notification impact from an intermediary-facilitated public-private mix intervention in Viet Nam: a population-level quasi-experimental study."*
   - *We have adjusted the language to state “was associated with” in various locations such as line 81 of the Abstract, line 101 of the Author summary, lines 411-414 as well as lines 542-543 of the Discussion.*
   - *We would, however, respectfully ask the Editor to reconsider the accompanying suggestion that causal language be reserved for randomized trials. Difference-in-differences is a quasi-experimental design whose explicit purpose is to identify causal effects and specifically the average treatment effect on the treated (ATT), in settings where “randomization is infeasible or unethical,” as is the case for a nationally deployed public–private mix intervention and similar policy-level interventions [1,2].*
   - *Causal identification here is grounded in the stated and testable assumptions (parallel trends, no anticipation, and stable treatment value), and we have made the credibility of these assumptions the center of our analysis through event-study pre-trend diagnostics, restricted-control and alternative-COVID robustness checks, and independent triangulation against national interrupted time-series estimates.*
   - *Our estimator was intentionally chosen to yield unbiased causal effects under staggered rollout and treatment-effect heterogeneity, conditions under which conventional two-way fixed-effects estimates are known to be biased [3–6].*
   - *Hernán has argued that substituting associational euphemisms for an explicitly causal objective does not improve inference and can obscure the scientific question a study was designed to answer [7], which reflects our intent to ensure the use of appropriate causal language based on the validity of doing in the previous bullet.*
   - *We also note a previously published a methodologically analogous difference-in-differences evaluation of a national PPM programme published by your journal [8].*
   - *We therefore hope to be able to retain the causal framing in the revised title and text, while ensuring throughout that our interpretation is explicitly conditioned on the identifying assumptions, and that we do not claim equivalence to a randomized trial.*
9. Please ensure that your title is accessible to a general reader.
   - *In addition to the change in the study design above, we have changed the title to align with the prior PLoS Medicine publications [8,9] to state: “Evaluation of the tuberculosis notification impact from an intermediary-facilitated public-private mix intervention in Viet Nam: a population-level quasi-experimental study.”*
10. Please confirm that your abstract complies with our requirements, including format (three sections: Background, Methods and Findings, and Conclusions) and providing all the information relevant to this study type <https://journals.plos.org/plosmedicine/s/submission-guidelines#loc-abstract>. Abstracts may not exceed 500 words.
    - *We confirm that the abstract includes only the three permitted sections with a word count of 453. Nevertheless, we have capitalized the “F” in Findings as per this feedback.*
11. Please ensure that the Introduction ends with a clear description of the study question or hypothesis.
    - *As per the prior editorial request, the last sentence of the Background was edited to state the objective through more accessible language: “This study evaluates the population-level impact of PPM through the use of unbiased analytical methods to account for heterogeneity from the intervention’s staggered deployment and dynamic intensity.”*
12. Please ensure that all abbreviations are defined at first use throughout the text.
    - *We have inserted a definition for “confidence interval” in line 69 to mirror the definition provided in line 350.*
13. Please confirm that all numbers presented in the abstract are present and identical to numbers presented in the main manuscript text.
    - *We confirm that all numbers presented in the abstract are present and identical to numbers presented in the main manuscript.*
14. Please include a statement regarding use or non-use of AI tools and technologies at the end of the Methods. Please specify the name(s) and version(s) of any tool(s) used and a description of their use, including how the AI-assisted or generated content was evaluated by authors, and a clear statement of which aspects of the study, article contents, data, or supporting files were affected or generated by AI tool usage. Please also include a statement specifying who retains responsibility for the final manuscript. See our guidelines for more information: <https://journals.plos.org/plosmedicine/s/ethical-publishing-practice#loc-artificial-intelligence-tools-and-technologies>.
    - *We have added a declaration about the use of AI tools in lines 307-312 of the Methods: “Anthropic's Claude (version 4; Opus) and OpenAI’s ChatGPT (GPT-4) were used to improve the readability and language of the manuscript and to assist with debugging and annotating analysis code, which was verified by the authors. The tool was not used to design the study, analyze or interpret data, or draw scientific conclusions. We reviewed all output and take full responsibility for the content of this manuscript.”*
15. Please remove the 'conclusions' subheading from the discussion. Please also remove any other subheadings from the discussion.
    - *We have removed the Conclusions subheading in line 538 and confirm that the discussion includes no other subheadings.*
16. Statistical reporting: Please revise throughout the manuscript, including tables and figures.

- Please report statistical information as follows to improve clarity for the reader "22% (95% CI [13,28]; p</=)".
- Please separate upper and lower bounds with commas instead of hyphens as the latter can be confused with reporting of negative values.
- Please repeat statistical definitions (HR, CI etc.) for each set of parentheses.
  - *We confirm that all statistical values have been reported in the format provided with upper and lower bounds separated by a comma and statistical definitions defined on first use and abbreviations repeated for each set of parentheses thereafter.*

17. In the abstract, please include the important dependent variables that are adjusted for in the analyses.
    - *For the primary analysis, we included a description of the adjustment parameters in lines 63-64: “The analysis adjusted for province and calendar-quarter fixed effects, and for COVID-19 disruptions.”*
18. It appears that one or more study authors is affiliated with one or more of the agencies that funded the study. Thus, the statement “The funders had no role in study design, data collection and analysis, decision to publish, or preparation of the manuscript” does not apply. Please revise the Financial Disclosure accordingly, as in "[Author name] is [author's role] at [funding agency]. The funders had no other role in study design…..”
    - *A corresponding text with all the relevant authors and their roles at their respective funding agencies has been added to lines 571-575 as follows: “JC is Team lead and TG is Technical Officer at the Stop TB Partnership. MAY and JT are Senior Advisors, Tuberculosis, at The Global Fund to fight AIDS, TB and Malaria (The Global Fund). MHP was Project Management Specialist, HIV and TB, at the United States Agency for International Development (USAID). The funding agencies had no other role in the study design…”*
19. All authors must declare their relevant competing interests per the PLOS policy, which can be seen here: https://journals.plos.org/plosmedicine/s/competing-interests For authors with ties to industry, please indicate whether any of the interests has a financial stake in the results of the current study.
    - *Competing interests of the authors were declared in lines 579-594. We hope that these declarations suffice.*
20. PLOS defines the “minimal data set” to consist of the data set used to reach the conclusions drawn in the manuscript with related metadata and methods, and any additional data required to replicate the reported study findings in their entirety. Authors do not need to submit their entire data set, or the raw data collected during an investigation. Please submit the following data:

- The values behind the means, standard deviations and other measures reported;
- The values used to build graphs;
- The points extracted from images for analysis.
  - *Data are property of the National TB Control Program as part of the Government of Viet Nam, and can be accessed in coordination through the provided contact information in the Data availability section, and in compliance with applicable personal data protection directives and laws as outlined in the Data availability section in lines 596-608.*

21. Figures cannot be reproduced from other sources that are not CC-BY. Please provide a different figure for Fig. 2 if the license to use is not CC-BY.
    - *Fig. 2 has been created entirely from scratch and solely for this publication. It does not contain any material that is subject to any copyrights.*
22. Please provide titles and legends for all figures and tables, including those in Supporting Information files. Please define all acronyms used in each figure or table in its corresponding legend.
    - *We confirm that all figures and tables in the main manuscript and supporting information files are companied by titles and legends, while we have made relevant updates to include all acronyms used in the respective figures and tables, as needed.*
23. Figure 3: Where data points are discrete, please ensure that they are depicted in the figures as discrete data and not as a continuous line.
    - *The line has been removed as requested.*
24. Please confirm that the appropriate usage rights apply to the use of the maps in Figure 1 and elsewhere. Please see our guidelines for map images: <https://journals.plos.org/plosmedicine/s/figures#loc-maps>
    - *This is confirmed and has been clarified with the technical team.*
25. Please ensure that the study is reported according to the STROBE guideline, and include the completed STROBE checklist as Supporting Information. Please add the following statement, or similar, to the Methods: "This study is reported as per the Strengthening the Reporting of Observational Studies in Epidemiology (STROBE) guideline (S1 Checklist)."

- The STROBE guideline can be found here: <http://www.equator-network.org/reporting-guidelines/strobe/>
- When completing the checklist, please use section and paragraph numbers, rather than page numbers.
  - *The reference to the STROBE checklist was adjusted in lines 305-307 with the exact wording as requested above.*
  - *The checklist has been adjusted to state the relevant section and subsection by names and the specific paragraph numbers.*

26. Did your study have a prospective protocol or analysis plan? Please state this (either way) early in the Methods section.
a) If a prospective analysis plan (from your funding proposal, IRB or other ethics committee submission, study protocol, or other planning document written before analyzing the data) was used in designing the study, please include the relevant prospectively written document with your revised manuscript as a Supporting Information file to be published alongside your study, and cite it in the Methods section. A legend for this file should be included at the end of your manuscript.
b) If no such document exists, please make sure that the Methods section transparently describes when analyses were planned, and when/why any data-driven changes to analyses took place.
c) In either case, changes in the analysis-- including those made in response to peer review comments-- should be identified as such in the Methods section of the paper, with rationale.
   - *We stated at the beginning of the methods section that there was no prospective analysis plan. To meet your request, we have added a description of the initially intended analyses and their evolution to the final set of analyses in lines 246 to 253. The full opening paragraph now reads: “No a priori sample size was calculated for the intervention or prospective analysis plan developed for the study. Based on the study objectives, initial analysis strategies included traditional two-way fixed-effect modeling and pragmatic approaches to measuring the effects of interventions in quasi-experimental designs [10–12]. However, the staggered deployment and variable intensity of the intervention necessitated the application of recently established, unbiased analytical methods for policy evaluations. While these novel methods enhance internal validity of the causal relationships, they rely on the validity of key assumptions. Study procedures were expanded to include testing of these assumptions as further described below.”*
27. Your study is observational and therefore causality cannot be inferred. Please remove language that implies causality and refer to associations instead.
    - *As described under question 8, there is a scientific basis and precedence for the validity pf causality inferences based on observational studies, when randomization is unpractical or unethical, under adherence to analytical assumptions and triangulation of results.*
    - *We therefore respectfully request to retain the language as presented as it represents the foundational scientific method underlying the study findings and the manuscript.*
28. For all observational studies, in the manuscript text, please indicate: (1) the specific hypotheses you intended to test, (2) the analytical methods by which you planned to test them, (3) the analyses you actually performed, and (4) when reported analyses differ from those that were planned, transparent explanations for differences that affect the reliability of the study's results. If a reported analysis was performed based on an interesting but unanticipated pattern in the data, please be clear that the analysis was data-driven.
    1) *We have attached to clarify the specific hypotheses by clarifying the overall and specific study objectives in lines 155 to 157 as follows: “We conducted a non-randomized, quasi-experimental evaluation to assess the effect on TB notifications of a population-level PPM intervention implemented through an intermediary agency in Viet Nam. The specific study objectives were…”*
    2) *Based on feedback #26, we have expanded the description of the initial analysis strategy and the rationale of the subsequent change in analytical methods in the opening paragraph of the Statistical analyses section as highlighted above.*
    3) *The actual analyses have been described in subsequent paragraphs of the abovementioned section.*
    4) *Similarly, a sentence was added to highlight the changes in study procedures that aimed to enhance validity of the results.*

**Reviewer 2 comments**

Thank you for the careful revisions to your manuscript, which I think have strengthened it considerably. I do have a few remaining comments/suggestions.

1. The description of the intervention is substantially improved. I did have a specific question about the protocol used for active case-finding. The revised manuscript explains that screened persons with potential TB-related abnormalities on CXR were referred for molecular testing. If I have understood correctly, all screened persons underwent CXR as well as the WHO questionnaire. How were persons with positive symptom screens but "negative" CXRs handled? If they were not further investigated, this would imply that the CXR alone would have been sufficient for decision making.
   - *Thank you for this additional point, which we indeed did not address in the description of the intervention. We have added a sentence to cover the identified eventuality of symptom-positive persons with a normal CXR in lines 207-208: “Symptomatic persons without CXR abnormalities were eligible for conventional diagnostic tests such as smear microscopy and culture.”*
2. In terms of epidemiologic impact and scalability of the intervention, it would be useful to state more clearly the estimated overall percentage increase in TB notifications thought attributable to the intervention, in those provinces where it was undertaken. The increase in notifications per province-quarter is not easy to interpret in this sense. I divided the 3,206 additional notifications per year nationwide (presumably limited to the intervention provinces; line 361) by the 44,096 baseline notifications from the intervention provinces in 2019 to estimate an average 7% increase in annual notifications, recognizing that this of course does not account for changes in population size over the intervening period and may have other imperfections. But this type of simple metric to capture the overall population impact of the intervention, when applied at the scale described in the manuscript, would be very useful to readers. Of course, it is there implicitly and I was able to make this simple calculation, but I think making it explicit would further strengthen the manuscript. Another way to do this could be to add to Table 2 the mean "background" notifications per province-quarter, in the non-intervention provinces and/or in the intervention provinces during the quarters before the intervention was rolled out in each.
   - *Thank you for this keen observation. As suggested, we have adjusted Table 2 to include the mean baseline TB and PPM notifications per province-quarter in the intervention provinces for the six quarters prior to roll-out.*
3. What do the P-values for the marginal costs per additional notification refer to (lines 382-383? To me they seem unclear and unnecessary. The relevant information is captured by the point estimates and 95% confidence intervals.
   - *The addition of the p-values, which were simply transferred from the TB notification analysis, was an editorial choice made for completeness sake, but we fully concur with your assessment and have removed them as suggested.*
4. Lines 461-463: The authors assume that a full economic analysis would demonstrate cost savings to programs as well as to patients. But the intervention (appropriately) increases use of health care resources to test more people with possible TB, and to diagnose and treat more people with TB disease accordingly. So one would predict that the intervention is in fact associated with higher costs to health payers (e.g. the TB program), but lower costs to patients by avoiding lost productivity related to severe illness and death. Please reconsider/rephrase accordingly.
   - *We have adjusted the phrasing in lines 484-485 to now state: “Nevertheless, routine program scale-up processes as well as costs additionally incurred and potentially saved to programs and patients through a full health economic analysis…”*

**Technical requests**

1) [ Figure 1]: Thank you for stating "The map was created using QGIS software, with source shape files coming from GADM. The relevant link is here: https://gadm.org/download_country.html "

Please ensure the map source details are included in the figure legend.

NOTE: Currently the figure legend (Line no 733 to 741) does not include the map source details. Please check and amend the changes accordingly

- *We have added the abovementioned sentence to the map legend in lines 776-778 as requested.*

Thank you once again for your positive assessment of the manuscript and please do not hesitate to contact us if there are any additional requests.

Sincerely and on behalf of the study team,

Luan Vo

Friends for International TB Relief

[luan.vo@tbhelp.org](mailto:luan.vo@tbhelp.org)

**References**

1.	Dimick JB, Ryan AM. Methods for Evaluating Changes in Health Care Policy. JAMA. 2014;312: 2401. doi:10.1001/jama.2014.16153

2.	Wing C, Simon K, Bello-Gomez RA. Designing Difference in Difference Studies: Best Practices for Public Health Policy Research Keywords. Annual Review of Public Health Annu Rev Public Health. 2026;27: 29. doi:10.1146/annurev-publhealth

3.	Sun L, Abraham S. Estimating dynamic treatment effects in event studies with heterogeneous treatment effects. J Econom. 2021;225: 175–199. doi:10.1016/j.jeconom.2020.09.006

4.	de Chaisemartin C, D’Haultfœuille X. Two-way fixed effects estimators with heterogeneous treatment effects. 2020. doi:10.1257/aer.20181169

5.	Callaway B, Sant’Anna PHC. Difference-in-Differences with multiple time periods. J Econom. 2021;225: 200–230. doi:10.1016/j.jeconom.2020.12.001

6.	Wooldridge JM. Simple approaches to nonlinear difference-in-differences with panel data. Econom J. 2023;26: C31–C66. doi:10.1093/ectj/utad016

7.	Hernán MA. The C-Word: Scientific Euphemisms Do Not Improve Causal Inference From Observational Data. Am J Public Health. 2018;108: 616–619. doi:10.2105/AJPH.2018.304337

8.	Yu S, Sohn H, Kim HY, Kim H, Oh KH, Kim HJ, et al. Evaluating the impact of the nationwide public-private mix (PPM) program for tuberculosis under National Health Insurance in South Korea: A difference in differences analysis. PLoS Med. 2021;18: 1–17. doi:10.1371/journal.pmed.1003717

9.	Rummo PE, Echenique JA, Wu E, Mijanovich T, Desai SM, Bragg MA, et al. Impact of sugary drink taxes on beverage calories purchased in a national fast food restaurant chain: A quasi-experimental study. PLoS Med. 2026;23: e1004642. doi:10.1371/journal.pmed.1004642

10.	Handley MA, Lyles C, McCulloch C, Cattamanchi A. Selecting and Improving Quasi-Experimental Designs in Effectiveness and Implementation Research. SSRN. 2018. doi:10.1146/annurev-publhealth-040617-014128

11.	Kontopantelis E, Doran T, Springate DA, Buchan I, Reeves D. Regression based quasi-experimental approach when randomisation is not an option: Interrupted time series analysis. BMJ (Online). 2015;350: 1–4. doi:10.1136/bmj.h2750

12.	Blok L, Creswell J, Stevens R, Brouwer M, Ramis O, Weil O, et al. A pragmatic approach to measuring monitoring and evaluating interventions for improved tuberculosis case detection. Int Health. 2014;6: 181–188. doi:10.1093/inthealth/ihu055
