# Supplementary material for: Evaluation of the tuberculosis notification impact from an intermediary-facilitated public–private mix intervention in Viet Nam: A population-level quasi-experimental study
Source: PLoS Med. 2026 Sep 23;23(9):e1005144. doi: 10.1371/journal.pmed.1005144 (PMC13600524; doi:10.1371/journal.pmed.1005144)
Supplement: Peer review history

# Editor report: Decision Letter 0

**DOI:** 10.1371/journal.pmed.1005144.r001

**Authors:** Alison Farrell

2 Jun 2026

Dear Dr Vo,

Thank you for submitting your manuscript entitled "Population-level tuberculosis notification impact from an intermediary-facilitated public-private mix intervention in Viet Nam: a heterogeneity-robust difference-in-differences analysis of national surveillance data" for consideration by PLOS Medicine.

Your manuscript has now been evaluated by the PLOS Medicine editorial staff, and I am writing to let you know that we would like to send your submission out for external peer review.

However, before we can send your manuscript to reviewers, we need you to complete your submission by providing the metadata that is required for full assessment. To this end, please login to Editorial Manager where you will find the paper in the 'Submissions Needing Revisions' folder on your homepage. Please click 'Revise Submission' from the Action Links and complete all additional questions in the submission questionnaire.

For clinical studies, please upload a copy of your trial study protocol in English as a supporting information file. The study protocol should be the version submitted for approval to the institutional review board or ethics committee, should include any amendments to the study protocol, as well as the date of their approval by the institutional review or ethics committee. Please also detail any deviations from the study protocol in the Methods section of your manuscript. The editors will consider the protocol and study conduct prior to a final decision for external review.

Please re-submit your manuscript within two working days, i.e. by Jun 04 2026 11:59PM.

Login to Editorial Manager here: https://www.editorialmanager.com/pmedicine

Once your full submission is complete, your paper will undergo a series of checks in preparation for peer review. Once your manuscript has passed all checks it will be sent out for review.

Feel free to email us at plosmedicine@plos.org if you have any queries relating to your submission.

Kind regards,

Alison Farrell, Ph.D.

Senior Editor

PLOS Medicine

# Peer review: Decision Letter 1

**DOI:** 10.1371/journal.pmed.1005144.r002

**Authors:** Alison Farrell

7 Jul 2026

Dear Dr Vo,

Many thanks for submitting your manuscript "Population-level tuberculosis notification impact from an intermediary-facilitated public-private mix intervention in Viet Nam: a heterogeneity-robust difference-in-differences analysis of national surveillance data" (PMEDICINE-D-26-01810R1) to PLOS Medicine. The paper has been reviewed by subject experts and a statistician; their comments are included below and can also be accessed here: [LINK]

As you will see, the reviewers find the topic interesting and timely, but have noted numerous issues that must be addressed to strengthen the manuscript and potential impact of the study. After discussing the paper with the editorial team and an academic editor with relevant expertise, I'm pleased to invite you to revise the paper in response to all the reviewers' comments. We plan to send the revised paper to some or all of the original reviewers, and we cannot provide any guarantees at this stage regarding publication.

Please further note that we ask you to provide additional context and rationale for the study in a final sentence of the Background section of the Abstract, add a clear explanation of the intervention in the Methods and Findings section of the Abstract (e.g. for a general reader), add further discussion of the potential implications of the results in the Discussion.

In addition to these revisions, you may need to complete some formatting changes, which you will receive in a follow up email. A member of our team will be in touch with a set of requests shortly. If you do not receive a separate email within a few days, please assume that checks have been completed, and no additional changes are required.

When you upload your revision, please include a point-by-point response that addresses all of the reviewer and editorial points, indicating the changes made in the manuscript and either an excerpt of the revised text or the location (eg: page and line number) where each change can be found. Please also be sure to check the general editorial comments at the end of this letter and include these in your point-by-point response. When you resubmit your paper, please include a clean version of the paper as the main article file and a version with changes tracked as a marked-up manuscript. It may also be helpful to check the guidelines for revised papers at http://journals.plos.org/plosmedicine/s/revising-your-manuscript for any that apply to your paper.

We ask that you submit your revision by Jul 28 2026 11:59PM. However, if this deadline is not feasible, please contact me by email, and we can discuss a suitable alternative.

Don't hesitate to contact me directly with any questions (afarrell@plos.org).

Best regards,

Alison

Alison Farrell, Ph.D.

Senior Editor

PLOS Medicine

afarrell@plos.org

-----------------------------------------------------------

Comments from the reviewers:

Reviewer #1: This is an excellent, timely, and policy-relevant manuscript that will provide an important contribution to the TB and public-private mix literature. The study addresses a major evidence gap: whether engagement of private and other non-NTP providers can produce gains in TB notification at a national level, in contrast to the multiple available pilot-studies/ data. The scale of the intervention is particularly impressive, covering approximately 40% of Viet Nam's population, with a large comparison area. The methodological approach is strong and appropriate for the non-randomized design. I found the concordance between total TB notification effects and PPM notification effects especially persuasive for attribution.

I recommend publication. I have a few minor points, which serve more as suggestions for clarification.

First, because intervention provinces were purposively selected high-burden areas, the authors could further discuss the risk of residual confounding from political, administrative, or programmatic factors that may have influenced both selection and notification trends.

Second, the authors could better distinguish the relative contributions of increased screening/referral versus private treatment reporting.

Finally, the cost analysis would be strengthened by discussing implications for routine implementation, including health-system and patient costs not captured by the funder perspective. The strategy of intermediary agency as a crucial phase for TB interventions within public-private mix systems is clear. But a brief reflection in the discussion on the sustainability of this strategy versus other intermediary systems solutions (such as health insurance) will add to health systems strengthening lens. Routine program scale-up processes, costs saved to both programs as well as to patients, could be mentioned as areas for further research to strengthen the current evidence of engaging the private sector.

Overall, this is a high-quality manuscript with substantial relevance for current national TB planning (including Global Fund GC8), particularly in a period of constrained TB financing. It provides rare and much-needed population-level evidence that intermediary-supported PPM strategies can make a concrete and cost-effective contribution to TB notification. The discussion is a great reflection on the findings while also highlighting outstanding gaps in private sector engagement for contact tracing and treatment support.

Reviewer #2: This is an interesting manuscript on a topic of substantial public health relevance. It estimates the real-world impact of an intervention designed to strengthen links between private providers and the National Tuberculosis (TB) Program of Vietnam, with respect to increases in TB notifications. It also estimates the marginal cost per additional notification. It suggests that the intervention had a measurable impact on TB notifications to national public health authorities, by supporting suitable screening, diagnostic testing and reporting. This is an important message. That said, I have several comments about the manuscript in its current form.

1.Throughout the manuscript (methods, results, discussion) there is considerable emphasis on the statistical analyses used to detect/triangulate an increase in TB case notifications in provinces where the intervention was implemented, relative to what would otherwise have been expected over the same time period—and to distinguish this from increases potentially due to other factors. While methodologic rigor is of course essential, I perceive that some of this leads to the main text reading in a way that will be overly technical for many readers. Some of this detail could be further relegated to the appendix, to make space in the main text for necessary description and reflection around more public health- and program-oriented issues. The technical methods and detailed results should support policy, health services and public health; they should not become the main message, in my view.

2.A clear example is the lack of clarity around the intervention itself in the main manuscript—most notably who the target population consisted of, and what screening algorithm was used. It is stated, without further explanation, that "health-seeking individuals" who presented to participating providers were eligible for assessment. In reading the appendix several times, I gleaned that this assessment involved the standard 4-symptom screen, plus (as far as I can tell) chest X-rays (CXRs) for all those seen, regardless of the presence or absence of symptoms. But this is never clearly stated. If readers wish to judge the generalizability or scalability of the intervention reported here, this type of information is essential. It's also essential that the authors clearly spell out how these screening and assessment practices differed (or not) from those in the "control" settings, and from those in public sector facilities in both intervention and control provinces.

3.Similarly, there is little information about the process or factors that ultimately led some providers to participate, and others not to. Over half of those eligible were excluded as they were "unreached/not approached" [figure S4]. What does this mean? Why did only 26% of providers with a signed contract send at least one person for a CXR? And do the authors have any information, beyond place of practice, that allows them (and readers) to look for differences between eligible providers who did vs. did not participate, including those reached who declined participation? This relates directly to potential biases and to generalizability—topics that are not really addressed as potential limitations or concerns in the discussion section. Instead, the only limitations specifically addressed are of a technical/statistical nature.

4.Continuing with the same theme, what do these results tell us about potential scalability? In other words, the authors make a convincing argument that the intervention had a measurable impact on TB notifications to the national program. But this impact appears relatively modest, in absolute terms, as does the proportion of participating private providers. Based on their data and experience, does this intervention appear scalable, to reach more providers, and more people with possible TB? How likely is a similar intervention to work in other settings? Along those lines, it would be helpful to readers to better describe what exactly the intervention consisted of, and who did what; the description on page 9 is very broad: "The intermediary identified, contracted, and supported private providers through training, supervision, and access to diagnostic services, while facilitating systematic screening, referral, and reporting of presumptive TB cases."

5.The cost analysis requires some revision and reframing. The conceptual difference/differing definitions between the provincial and national estimates for marginal cost per additional notification are not explained, making the substantial difference between the provincial estimate ($166) and the national estimate ($458) very difficult to understand. In my own view, the additional cost per additional notification should be the same, if all the input costs have been suitably assigned and prorated. In addition, the perspective of the cost analysis is somewhat problematic. It focuses exclusively on the cost of services and training provided by or purchased by the intermediary. This might be relevant to third-party funders, but the intervention brings other costs that are directly relevant to the health system, and that would need to be considered by local/national decision makers contemplating adoption of such an intervention. Referrals by private providers to the public system for CXRs and molecular diagnostics, for example, are a desired outcome but they imply additional costs to the public system which are not captured by the current cost analysis, and again are directly relevant to questions of implementation and scalability. These additional costs to the health care system should be estimated, or at minimum discussed. And the authors should consider that the national program itself, rather than third party donor agencies, may in future take responsibility for costs and implementation of such an intervention.

6.The discussion section seems longer than necessary and, in my view, should focus more clearly on implications and limitations of this work, beyond technical issues related to the statistical analysis. As mentioned previously, the authors need to explicitly consider potential bias in the cascade of participating providers (and those who referred patients for CXRs), as well as questions of scalability, generalizability, and potential budget impact.

Minor comment:

1.Page 6, line 105: "legal escrow" seems an unusual term. Consider rephrasing.

Reviewer #3: The paper uses a difference in differences approach to assess the casual effect of PPM intervention on TB notifications using data from Viet Nam. It also applies an interrupted time series approach as a secondary analysis. Overall, the statistical methods are appropriate, and are generally well reported and presented. There is a good discussion of the assumptions and acknowledgement of the study's limitations. I have the following comments around the models used, and how these reflect the intervention made in practice.

- Line 162 notes dynamic changes in intervention intensity. Could these dynamic changes be elaborated? How was this incorporated into the modelling? It might be useful to provide the difference in differences modelling equation, perhaps part of Section S1.4, in order to illustrate whether, and how, this changing intensity was accounted for.

- Similarly, for the interrupted time series model, how was the intervention period modelled - was the intervention included as a single time point, or was the dynamic intervention period accounted for? It might again be useful to provide the modelling equation in Section S1.7 to show how this was implemented.

- Some more detail on the selection of provinces for intervention would be beneficial. In Line 145 it is mentioned they were selected as high-burden settings, but this isn't explained. This would help to give context to the intervention being analysed, and for evaluation of the potential for residual confounding.

Any attachments provided with reviews can be seen via the following link: [LINK]

--------------------------------------------------------- ---

General editorial requests:

(Note: not all will apply to your paper, but please check each item carefully)

*We ask every co-author listed on the manuscript to fill in a contributing author statement, making sure to declare all competing interests. If any of the co-authors have not filled in the statement, we will remind them to do so when the paper is revised. If all statements are not completed in a timely fashion this could hold up the re-review process. If new competing interests are declared later in the revision process, this may also hold up the submission. Should there be a problem getting one of your co-authors to fill in a statement we will be in contact. Please do not add or remove authors without first discussing this with the handling editor. You can see our competing interests policy here: http://journals.plos.org/plosmedicine/s/competing-interests.

*Please upload any figures associated with your paper as individual TIF or EPS files with 300dpi resolution at resubmission; please read our figure guidelines for more information on our requirements: http://journals.plos.org/plosmedicine/s/figures. While revising your submission, we strongly recommend that you use PLOS's NAAS tool (https://ngplosjournals.pagemajik.ai/artanalysis) to test your figure files. NAAS can convert your figure files to the TIFF file type and meet basic requirements (such as print size, resolution), or provide you with a report on issues that do not meet our requirements and that NAAS cannot fix.

After uploading your figures to PLOS's NAAS tool - https://ngplosjournals.pagemajik.ai/artanalysis, NAAS will process the files provided and display the results in the "Uploaded Files" section of the page as the processing is complete.

If the uploaded figures meet our requirements (or NAAS is able to fix the files to meet our requirements), the figure will be marked as "fixed" above. If NAAS is unable to fix the files, a red "failed" label will appear above.

When NAAS has confirmed that the figure files meet our requirements, please download the file via the download option, and include these NAAS processed figure files when submitting your revised manuscript.

*Please ensure that the paper adheres to the PLOS Data Availability Policy (see http://journals.plos.org/plosmedicine/s/data-availability), which requires that all data underlying the study's findings be provided in a repository or as Supporting Information. For data residing with a third party, authors are required to provide instructions with contact information (web or email address) for obtaining the data. Please note that a study author cannot be the contact person for the data. PLOS journals do not allow statements supported by "data not shown" or "unpublished results." For such statements, authors must provide supporting data or cite public sources that include it.

*We expect all researchers with submissions to PLOS in which author-generated code underpins the findings in the manuscript to make all author-generated code available without restrictions upon publication of the work. In cases where code is central to the manuscript, we require the code to be made available as a condition of publication. Authors are responsible for ensuring that the code is reusable and well documented. Please make any custom code available, either as part of your data deposition or as a supplementary file. Please add a sentence to your data availability statement regarding any code used in the study, e.g. "The code used in the analysis is available from Github [URL] and archived in Zenodo [DOI link]" Please review our guidelines at https://journals.plos.org/plosmedicine/s/materials-software-and-code-sharing and ensure that your code is shared in a way that follows best practice and facilitates reproducibility and reuse. Because Github depositions can be readily changed or deleted, we encourage you to make a permanent DOI'd copy (e.g. in Zenodo) and provide the URL.

*Please separate the funding statement from the competing interests statement.

* Please provide a URL for each funder. Please also provide an email for a contact for access to the data. The contact may not be a study author.

* If new code was generated for these analyses, please deposit it in a public repository and provide a URL and DOI.

* Please include a disclosure pertaining to any use of AI in assembling the manuscript or analyzing or generating the data.

FORMATTING - GENERAL

*Abstract: Please structure your abstract using the PLOS Medicine headings (Background, Methods and Findings, Conclusions). Please combine the Methods and Findings sections into one section.

*At this stage, we ask that you include a short, non-technical Author Summary of your research to make findings accessible to a wide audience that includes both scientists and non-scientists. The Author Summary should immediately follow the Abstract in your revised manuscript. This text is subject to editorial change and should be distinct from the scientific abstract. Ideally each sub-heading should contain 2-3 single sentence, concise bullet points containing the most salient points from your study.

* Please add bullet points to your Author Summary.

* In the final bullet point of 'What Do These Findings Mean?', please include the main limitations of the study in non-technical language. Please see our author guidelines for more information: https://journals.plos.org/plosmedicine/s/revising-your-manuscript#loc-author-summary.

*Please express the main results with 95% CIs as well as p values. When reporting p values please report as p<0.001 and where higher as the exact p value p=0.002, for example. Throughout, suggest reporting statistical information as follows to improve clarity for the reader "22% (95% CI [13%,28%]; p</=)". Please be sure to define all numerical values at first use.

*Please include page numbers and line numbers in the manuscript file. Use continuous line numbers (do not restart the numbering on each page).

*Please cite the reference numbers in square brackets. Citations should precede punctuation.

FIGURES AND TABLES

*Please provide titles and legends for all figures and tables (including those in Supporting Information files).

*Please define all abbreviations used in each figure/table (including those in Supporting Information files).

*Please consider avoiding the use of red and green in order to make your figure more accessible to those with color blindness.

* Please ensure that the figures do not involve copyrighted material (e.g. Fig.1, 2).

SUPPLEMENTARY MATERIAL

*Please note that supplementary material will be posted as supplied by the authors. Therefore, please amend it according to the relevant comments outlined here.

*Please cite your Supporting Information as outlined here: https://journals.plos.org/plosmedicine/s/supporting-information

REFERENCES

*PLOS uses the numbered citation (citation-sequence) method and first six authors, et al.

*Please ensure that journal name abbreviations match those found in the National Center for Biotechnology Information (NCBI) databases (http://www.ncbi.nlm.nih.gov/nlmcatalog/journals), and are appropriately formatted and capitalised.

*Where website addresses are cited, please include the complete URL and specify the date of access (e.g. [accessed: 12/06/2023]).

*Please also see https://journals.plos.org/plosmedicine/s/submission-guidelines#loc-references for further details on reference formatting.

OBSERVATIONAL STUDIES

*Abstract: Please include the study design, population and setting, number of participants, years during which the study took place (enrollment and follow up), length of follow up, and main outcome measures.

*Please ensure that the study is reported according to the STROBE (or appropriate STOBE extension) guideline (available from: https://www.equator-network.org/reporting-guidelines/strobe) and include the completed STROBE (or STROBE extension) checklist as Supporting Information. Please add the following statement, or similar, to the Methods: "This study is reported as per the Strengthening the Reporting of Observational Studies in Epidemiology (STROBE) guideline (S1 Checklist)." When completing the checklist, please use section and paragraph numbers, rather than page numbers.

*[FOR POPULATION HEALTH/REGISTRY STUDIES] Please ensure that the study is reported according to the RECORD guideline (available from https://www.record-statement.org) and include the completed checklist as Supporting Information. Please add the following statement, or similar, to the Methods: "This study is reported as per the Reporting of Studies Conducted using Observational Routinely-Collected Data (RECORD) guideline (S1 Checklist)." When completing the checklist, please use section and paragraph numbers, rather than page numbers.

*[FOR POPULATION HEALTH ESTIMATES] Please ensure that the study is reported according to the GATHER statement (available from https://www.equator-network.org/reporting-guidelines/gather-statement) and include the completed checklist as Supporting Information. Please add the following statement, or similar, to the Methods: "This study is reported as per the Guidelines for Accurate and Transparent Health Estimates Reporting (GATHER) statement (S1 Checklist)." When completing the checklist, please use section and paragraph numbers, rather than page numbers.

*[FOR MEDIATION ANALYSES] We recommend that the study is reported according to the AGReMA statement (https://agrema-statement.org/#:~:text=AGReMA%20is%20an%20evidence%2D%20and,randomised%20trials%20and%20observational%20studies) and include the completed checklist as Supporting Information. Please add the following statement, or similar, to the Methods: "This study is reported as per the Guideline for Reporting Mediation Analyses (AGReMA) statement (S1 Checklist)." When completing the checklist, please use section and paragraph numbers, rather than page numbers.

*For all observational studies, in the manuscript text, please indicate: (1) the specific hypotheses you intended to test, (2) the analytical methods by which you planned to test them, (3) the analyses you actually performed, and (4) when reported analyses differ from those that were planned, transparent explanations for differences that affect the reliability of the study's results. If a reported analysis was performed based on an interesting but unanticipated pattern in the data, please be clear that the analysis was data driven.

*Please state in the Methods section whether the study had a prospective protocol or analysis plan. If a prospective analysis plan (from your funding proposal, IRB or other ethics committee submission, study protocol, or other planning document written before analyzing the data) was used in designing the study, please include the relevant document(s) with your revised manuscript as a Supporting Information file to be published alongside your study and cite it in the Methods section. A legend for this file should be included at the end of your manuscript. If no such document exists, please make sure that the Methods section transparently describes when analyses were planned, and when/why any data-driven changes to analyses took place. Changes in the analysis, including those made in response to peer review comments, should be identified as such in the Methods section of the paper, with rationale.

MODELLING STUDIES

The following list is derived from Geoffrey P Garnett, Simon Cousens, Timothy B Hallett, Richard Steketee, Neff Walker. Mathematical models in the evaluation of health programmes. (2011) Lancet DOI:10.1016/S0140-6736(10)61505-X:

*If pertinent, please provide a diagram that shows the model structure, including how the natural history of the disease is represented, the process and determinants of disease acquisition, and how the putative intervention could affect the system.

*Please provide a complete list of model parameters, including clear and precise descriptions of the meaning of each parameter, together with the values or ranges for each, with justification or the primary source cited and important caveats about the use of these values noted.

*Please provide a clear statement about how the model was fitted to the data, including goodness-of-fit measure, the numerical algorithm used, which parameter varied, constraints imposed on parameter values, and starting conditions.

*For uncertainty analyses, please state the sources of uncertainties quantified and not quantified [can include parameter, data, and model structure].

*Please provide sensitivity analyses to identify which parameter values are most important in the model. Uncertainty estimates seek to derive a range of credible results on the basis of an exploration of the range of reasonable parameter values. The choice of method should be presented and justified.

*Please discuss the scientific rationale for the choice of model structure and identify points where this choice could influence conclusions drawn. Please also describe the strength of the scientific basis underlying the key model assumptions.

*For studies that develop a prediction model or evaluate its performance, please ensure that the study is reported according to the TRIPOD statement (https://www.equator-network.org/reporting-guidelines/tripod-statement) and include the completed checklist as Supporting Information. Please add the following statement, or similar, to the Methods: "This study is reported as per the Transparent Reporting of a Multivariable Prediction Model for Individual Prognosis Or Diagnosis (TRIPOD) statement (S1 Checklist)." For studies using machine learning, please use the TRIPOD-AI checklist. When completing the checklist, please use section and paragraph numbers, rather than page numbers.

HEALTH ECONOMICS / COST-EFFECTIVENESS STUDIES

*Please ensure that the study is reported according to the CHEERS guideline (available from: https://www.equator-network.org/reporting-guidelines/cheers) and include the completed checklist as Supporting Information. Please add the following statement, or similar, to the Methods: "This study is reported as per the Strengthening the Consolidated Health Economic Evaluation Reporting Standards 2022 (CHEERS 2022) Statement (S1 Checklist)." When completing the checklist, please use section and paragraph numbers, rather than page numbers.

# Author comment: Author response to Decision Letter 2

**DOI:** 10.1371/journal.pmed.1005144.r003

22 Jul 2026

Attachment Submitted filename: Response letter_final.docx

# Peer review: Decision Letter 2

**DOI:** 10.1371/journal.pmed.1005144.r004

**Authors:** Alison Farrell

17 Aug 2026

Dear Dr. Vo,

Thank you very much for re-submitting your manuscript "Population-level tuberculosis notification impact from an intermediary-facilitated public-private mix intervention in Viet Nam: a heterogeneity-robust difference-in-differences analysis of national surveillance data" (PMEDICINE-D-26-01810R2) for review by PLOS Medicine.

I have discussed the paper with my colleagues and the academic editor and it was also seen again by three reviewers. I am pleased to say that provided the remaining editorial and production issues, as well as the additional clarifications requested by reviewer 2, are dealt with we are planning to accept the paper for publication in the journal.

The remaining issues that need to be addressed are listed at the end of this email. Any accompanying reviewer attachments can be seen via the link below. Please take these into account before resubmitting your manuscript and return a point-by-point response to all editorial and reviewer requests:

[LINK]

***Please note while forming your response, if your article is accepted, you may have the opportunity to make the peer review history publicly available. The record will include editor decision letters (with reviews) and your responses to reviewer comments. If eligible, we will contact you to opt in or out.***

In revising the manuscript for further consideration here, please ensure you address the specific points made by each reviewer and the editors. In your rebuttal letter you should indicate your response to the reviewers' and editors' comments and the changes you have made in the manuscript. Please submit a clean version of the paper as the main article file. A version with changes marked must also be uploaded as a marked up manuscript file.

Please also check the guidelines for revised papers at http://journals.plos.org/plosmedicine/s/revising-your-manuscript for any that apply to your paper. If you haven't already, we ask that you provide a short, non-technical Author Summary of your research to make findings accessible to a wide audience that includes both scientists and non-scientists. The Author Summary should immediately follow the Abstract in your revised manuscript. This text is subject to editorial change and should be distinct from the scientific abstract.

In addition to these revisions, you may need to complete some formatting changes, which you will receive in a follow up email. A member of our team will be in touch with a set of requests shortly. If you do not receive a separate email within a few days, please assume that checks have been completed, and no additional changes are required.

We expect to receive your revised manuscript within 1 week. Please email us (plosmedicine@plos.org) if you have any questions or concerns.

We ask every co-author listed on the manuscript to fill in a contributing author statement. If any of the co-authors have not filled in the statement, we will remind them to do so when the paper is revised. If all statements are not completed in a timely fashion this could hold up the re-review process. Should there be a problem getting one of your co-authors to fill in a statement we will be in contact. YOU MUST NOT ADD OR REMOVE AUTHORS UNLESS YOU HAVE ALERTED THE EDITOR HANDLING THE MANUSCRIPT TO THE CHANGE AND THEY SPECIFICALLY HAVE AGREED TO IT.

Please ensure that the paper adheres to the PLOS Data Availability Policy (see http://journals.plos.org/plosmedicine/s/data-availability), which requires that all data underlying the study's findings be provided in a repository or as Supporting Information. For data residing with a third party, authors are required to provide instructions with contact information for obtaining the data. PLOS journals do not allow statements supported by "data not shown" or "unpublished results." For such statements, authors must provide supporting data or cite public sources that include it.

To enhance the reproducibility of your results, we recommend that you deposit your laboratory protocols in protocols.io, where a protocol can be assigned its own identifier (DOI) such that it can be cited independently in the future. Additionally, PLOS ONE offers an option to publish peer-reviewed clinical study protocols. Read more information on sharing protocols at https://plos.org/protocols?utm_medium=editorial-email&utm_source=authorletters&utm_campaign=protocols

Please review your reference list to ensure that it is complete and correct. If you have cited papers that have been retracted, please include the rationale for doing so in the manuscript text, or remove these references and replace them with relevant current references. Any changes to the reference list should be mentioned in the rebuttal letter that accompanies your revised manuscript.

Please note, when your manuscript is accepted, an uncorrected proof of your manuscript will be published online ahead of the final version, unless you've already opted out via the online submission form. If, for any reason, you do not want an earlier version of your manuscript published online or are unsure if you have already indicated as such, please let the journal staff know immediately at plosmedicine@plos.org.

If you have any questions in the meantime, please contact me or the journal staff on plosmedicine@plos.org.

We look forward to receiving the revised manuscript by Sep 07 2026 11:59PM.

Sincerely,

Alison Farrell, Ph.D.

Senior Editor

PLOS Medicine

plosmedicine.org

------------------------------------------------------------

Requests from Editors:

Line 46: revise ‘NTP’s’ to ‘NTPs’

Line 49-50: please revise “through the use of heterogeneity-robust econometric methods.” to be accessible to a general reader. Why is this aspect important in the Background of the Abstract?

Line 51: Revise to present tense, i.e. ‘We performed a non-randomized, quasi-…”

Line 58: Please clarify the time frame in relation to the intervention.

Line 76: please use associative rather than causal language.

Lines 104-105: Please revise phrasing of the last sentence in the Author summary to be accessible to a general reader.

Line 112: please clarify what is meant by ‘provider-induced’

GENERAL EDITORIAL REQUESTS

* Please confirm that your title complies with PLOS Medicine's style. Your title must be nondeclarative and not a question. It should begin with main concept if possible. "Effect of" should be used only if causality can be inferred, i.e., for an RCT. Please place the study design ("A randomized controlled trial," "A retrospective study," "A modelling study," etc.) in the subtitle (ie, after a colon).

** Please ensure that your title is accessible to a general reader.

* Please confirm that your abstract complies with our requirements, including format (three sections: Background, Methods and Findings, and Conclusions) and providing all the information relevant to this study type https://journals.plos.org/plosmedicine/s/submission-guidelines#loc-abstract

Abstracts may not exceed 500 words.

* Please ensure that the Introduction ends with a clear description of the study question or hypothesis.

* Please ensure that all abbreviations are defined at first use throughout the text.

* Please confirm that all numbers presented in the abstract are present and identical to numbers presented in the main manuscript text.

GENERAL

* Please include a statement regarding use or non-use of AI tools and technologies at the end of the Methods. Please specify the name(s) and version(s) of any tool(s) used and a description of their use, including how the AI-assisted or generated content was evaluated by authors, and a clear statement of which aspects of the study, article contents, data, or supporting files were affected or generated by AI tool usage. Please also include a statement specifying who retains responsibility for the final manuscript. See our guidelines for more information: https://journals.plos.org/plosmedicine/s/ethical-publishing-practice#loc-artificial-intelligence-tools-and-technologies.

* Please remove the 'conclusions' subheading from the discussion. Please also remove any other subheadings from the discussion.

* Statistical reporting: Please revise throughout the manuscript, including tables and figures.

- Please report statistical information as follows to improve clarity for the reader "22% (95% CI [13,28]; p</=)".

- Please separate upper and lower bounds with commas instead of hyphens as the latter can be confused with reporting of negative values.

- Please repeat statistical definitions (HR, CI etc.) for each set of parentheses.

* In the abstract, please include the important dependent variables that are adjusted for in the analyses.

Funding statement:

* It appears that one or more study authors is affiliated with one or more of the agencies that funded the study. Thus, the statement “The funders had no role in study design, data collection and analysis, decision to publish, or preparation of the manuscript” does not apply. Please revise the Financial Disclosure accordingly, as in "[Author name] is [author's role] at [funding agency]. The funders had no other role in study design…..”

COMPETING INTERESTS STATEMENT

* All authors must declare their relevant competing interests per the PLOS policy, which can be seen here: https://journals.plos.org/plosmedicine/s/competing-interests For authors with ties to industry, please indicate whether any of the interests has a financial stake in the results of the current study.

DATA AVAILABILITY

* PLOS defines the “minimal data set” to consist of the data set used to reach the conclusions drawn in the manuscript with related metadata and methods, and any additional data required to replicate the reported study findings in their entirety. Authors do not need to submit their entire data set, or the raw data collected during an investigation. Please submit the following data:

The values behind the means, standard deviations and other measures reported;

The values used to build graphs;

The points extracted from images for analysis.

FIGURES

* Figures cannot be reproduced from other sources that are not CC-BY. Please provide a different figure for Fig. 2 if the license to use is not CC-BY.

* Please provide titles and legends for all figures and tables, including those in Supporting Information files. Please define all acronyms used in each figure or table in its corresponding legend.

* Figure 3: Where data points are discrete, please ensure that they are depicted in the figures as discrete data and not as a continuous line.

* Please confirm that the appropriate usage rights apply to the use of the maps in Figure 1 and elsewhere. Please see our guidelines for map images: https://journals.plos.org/plosmedicine/s/figures#loc-maps

OBSERVATIONAL, COHORT, CROSS-SECTIONAL, AND CASE CONTROL STUDIES

* Please ensure that the study is reported according to the STROBE guideline, and include the completed STROBE checklist as Supporting Information. Please add the following statement, or similar, to the Methods: "This study is reported as per the Strengthening the Reporting of Observational Studies in Epidemiology (STROBE) guideline (S1 Checklist)."

The STROBE guideline can be found here: http://www.equator-network.org/reporting-guidelines/strobe/

When completing the checklist, please use section and paragraph numbers, rather than page numbers.

* Did your study have a prospective protocol or analysis plan? Please state this (either way) early in the Methods section.

a) If a prospective analysis plan (from your funding proposal, IRB or other ethics committee submission, study protocol, or other planning document written before analyzing the data) was used in designing the study, please include the relevant prospectively written document with your revised manuscript as a Supporting Information file to be published alongside your study, and cite it in the Methods section. A legend for this file should be included at the end of your manuscript.

b) If no such document exists, please make sure that the Methods section transparently describes when analyses were planned, and when/why any data-driven changes to analyses took place.

c) In either case, changes in the analysis-- including those made in response to peer review comments-- should be identified as such in the Methods section of the paper, with rationale.

* Your study is observational and therefore causality cannot be inferred. Please remove language that implies causality and refer to associations instead.

* For all observational studies, in the manuscript text, please indicate: (1) the specific hypotheses you intended to test, (2) the analytical methods by which you planned to test them, (3) the analyses you actually performed, and (4) when reported analyses differ from those that were planned, transparent explanations for differences that affect the reliability of the study's results. If a reported analysis was performed based on an interesting but unanticipated pattern in the data, please be clear that the analysis was data-driven.

Comments from Reviewers:

Reviewer #1: The authors have responded thoughtfully and comprehensively to the reviewers' comments. My principal concerns regarding residual confounding, sustainability, and the broader health-system implications have been satisfactorily addressed. The intervention is now described much more clearly, and the discussion provides stronger policy relevance. I would recommend publication.

Reviewer #2: Thank you for the careful revisions to your manuscript, which I think have strengthened it considerably. I do have a few remaining comments/suggestions.

1.The description of the intervention is substantially improved. I did have a specific question about the protocol used for active case-finding. The revised manuscript explains that screened persons with potential TB-related abnormalities on CXR were referred for molecular testing. If I have understood correctly, all screened persons underwent CXR as well as the WHO questionnaire. How were persons with positive symptom screens but "negative" CXRs handled? If they were not further investigated, this would imply that the CXR alone would have been sufficient for decision making.

2.In terms of epidemiologic impact and scalability of the intervention, it would be useful to state more clearly the estimated overall percentage increase in TB notifications thought attributable to the intervention, in those provinces where it was undertaken. The increase in notifications per province-quarter is not easy to interpret in this sense. I divided the 3,206 additional notifications per year nationwide (presumably limited to the intervention provinces; line 361) by the 44,096 baseline notifications from the intervention provinces in 2019 to estimate an average 7% increase in annual notifications, recognizing that this of course does not account for changes in population size over the intervening period and may have other imperfections. But this type of simple metric to capture the overall population impact of the intervention, when applied at the scale described in the manuscript, would be very useful to readers. Of course, it is there implicitly and I was able to make this simple calculation, but I think making it explicit would further strengthen the manuscript. Another way to do this could be to add to Table 2 the mean "background" notifications per province-quarter, in the non-intervention provinces and/or in the intervention provinces during the quarters before the intervention was rolled out in each.

3.What do the P-values for the marginal costs per additional notification refer to (lines 382-383? To me they seem unclear and unnecessary. The relevant information is captured by the point estimates and 95% confidence intervals.

4.Lines 461-463: The authors assume that a full economic analysis would demonstrate cost savings to programs as well as to patients. But the intervention (appropriately) increases use of health care resources to test more people with possible TB, and to diagnose and treat more people with TB disease accordingly. So one would predict that the intervention is in fact associated with higher costs to health payers (e.g. the TB program), but lower costs to patients by avoiding lost productivity related to severe illness and death. Please reconsider/rephrase accordingly.

Reviewer #3: I thank the authors for their response to my previous comments, which have now been satisfactorily addressed.

Any attachments provided with reviews can be seen via the following link:

[LINK]

# Author comment: Author response to Decision Letter 3

**DOI:** 10.1371/journal.pmed.1005144.r005

26 Aug 2026

Attachment Submitted filename: Second response letter.docx

# Editor report: Decision Letter 3

**DOI:** 10.1371/journal.pmed.1005144.r006

**Authors:** Alison Farrell

27 Aug 2026

Dear Dr Vo,

On behalf of my colleagues and the Academic Editor, Shenglan Tang, we are pleased to inform you that we have agreed to publish your manuscript "Evaluation of the tuberculosis notification impact from an intermediary-facilitated public-private mix intervention in Viet Nam: a population-level quasi-experimental study" (PMEDICINE-D-26-01810R3) in PLOS Medicine.

Before your manuscript can be formally accepted you will need to complete some formatting changes, which you will receive in a follow up email. Please be aware that it may take several days for you to receive this email; during this time no action is required by you. Once you have received these formatting requests, please note that your manuscript will not be scheduled for publication until you have made the required changes.

*Please note the following point in your Abstract and correct if necessary in the proof:

line 63: should 'repeating' be 'repeated'?

The sentence on lines 483-485 may be missing a verb.

In the meantime, please log into Editorial Manager at http://www.editorialmanager.com/pmedicine/, click the "Update My Information" link at the top of the page, and update your user information to ensure an efficient production process.

PRESS

We frequently collaborate with press offices. If your institution or institutions have a press office, please notify them about your upcoming paper at this point, to enable them to help maximise its impact. If the press office is planning to promote your findings, we would be grateful if they could coordinate with medicinepress@plos.org. If you have not yet opted out of the early version process, we ask that you notify us immediately of any press plans so that we may do so on your behalf.

We also ask that you take this opportunity to read our Embargo Policy regarding the discussion, promotion and media coverage of work that is yet to be published by PLOS. As your manuscript is not yet published, it is bound by the conditions of our Embargo Policy. Please be aware that this policy is in place both to ensure that any press coverage of your article is fully substantiated and to provide a direct link between such coverage and the published work. For full details of our Embargo Policy, please visit http://www.plos.org/about/media-inquiries/embargo-policy/.

To enhance the reproducibility of your results, we recommend that you deposit your laboratory protocols in protocols.io, where a protocol can be assigned its own identifier (DOI) such that it can be cited independently in the future. Additionally, PLOS ONE offers an option to publish peer-reviewed clinical study protocols. Read more information on sharing protocols at https://plos.org/protocols?utm_medium=editorial-email&utm_source=authorletters&utm_campaign=protocols

Thank you again for submitting to PLOS Medicine. We look forward to publishing your paper.

Sincerely,

Alison Farrell, Ph.D.

Senior Editor

PLOS Medicine

afarrell@plos.org
